# Assessing and Interpreting the Within-Body Biogeography of Human Microbiome Diversity

**DOI:** 10.3389/fmicb.2018.01619

**Published:** 2018-08-07

**Authors:** Zhanshan (Sam) Ma, Lianwei Li, Wendy Li

**Affiliations:** ^1^Computational Biology and Medical Ecology Lab, State Key Laboratory of Genetic Resources and Evolution, Kunming Institute of Zoology, Chinese Academy of Sciences, Kunming, China; ^2^Center for Excellence in Animal Evolution and Genetics, Chinese Academy of Sciences, Kunming, China

**Keywords:** diversity area relationship (DAR), within-body microbiome biogeography, power law, scale invariance, self-similarity, diversity area relationship (DAR) profile, maximal accrual diversity (MAD) profile, pair-wise diversity overlap (PDO) profile

## Abstract

A human body hosts a relatively independent microbiome including five major regional biomes (i.e., airway, oral, gut, skin, and urogenital). Each of them may possess different regional characteristics with important implications to our health and diseases (i.e., so-termed microbiome associated diseases). Nevertheless, these regional microbiomes are connected with each other through diffusions and migrations. Here, we investigate the within-body (intra-individual) distribution feature of microbiome diversity via diversity area relationship (DAR) modeling, which, to the best of our knowledge, has not been systematically studied previously. We utilized the Hill numbers for measuring alpha and beta-diversities and built 1,200 within-body DAR models with to date the most comprehensive human microbiome datasets of 18 sites from the human microbiome project (HMP) cohort. We established the intra-DAR profile (*z-q* pattern: the diversity scaling parameter *z* of the power law (PL) at diversity order *q* = 0–3), intra-PDO (pair-wise diversity overlap) profile (*g*-*q*), and intra-MAD (maximal accrual diversity) profile (*D*_max_-*q*) for the within-body biogeography of the human microbiome. These profiles constitute the “*maps*” of the within-body biogeography, and offer important insights on the within-body distribution of the human microbiome. Furthermore, we investigated the heterogeneity among individuals in their biogeography parameters and found that there is not an “average Joe” that can represent majority of individuals in a cohort or population. For example, we found that most individuals in the HMP cohort have relatively lower maximal accrual diversity (MAD) or in the “long tail” of the so-termed power law distribution. In the meantime, there are a small number of individuals in the cohort who possess disproportionally higher MAD values. These findings may have important implications for personalized medicine of the human microbiome associated diseases in practice, besides their theoretical significance in microbiome research such as establishing the baseline for the conservation of human microbiome.

## Introduction

The diversity-area relationship (DAR) (Ma, 2017) is a natural extension of the traditional species area relationship (SAR). The latter is well regarded as one of the few laws in ecology and biogeography and have been extensively studied in macro-ecology (e.g., Preston, 1960; Connor and McCoy, 1979; Rosenzweig, 1995; Lomolino, 2000; He and Legendre, 2002; Tjørve, 2003, 2009; Drakare et al., 2006; Tjørve and Tjørve, 2008; Harte et al., 2009; He and Hubbell, 2011; Sizling et al., 2011; Ma et al., 2012; Storch et al., 2012; Triantis et al., 2012; Whittaker and Triantis, 2012; Helmus et al., 2014). The expansion of SAR to microbial ecology (e.g., Green et al., 2004; Horner-Devin et al., 2004; Bell et al., 2005; Noguez et al., 2005; Peay et al., 2007; van der Gast et al., 2008; Lyons et al., 2010; Jones et al., 2012; Oliver et al., 2012; Pop Ristova et al., 2014; Zinger et al., 2014) was made possible largely by the high-throughput DNA sequencing technology, although less powerful molecular technologies such as FISH (Langendijk et al., 1995) T-RFLP (Liu et al., 1997), which were widely used for characterizing microbial communities before the NGS (next generation sequencing) technologies became readily accessible, also played a role in early days.

The later development of SAR in microbial world was because majority of bacteria in nature are still uncultivable and consequently they are not detectable without resorting to the sequencing technology or other lesser powerful molecular marking technologies such as FISH and T-RFLP. It is the metagenomics technology, which can efficiently sequence the genomes of nearly all species in a microbial community sample, that made it possible for the US NIH and European Union to launch the human microbiome project (HMP) and MetaHIT (Metagenomics of the Human Intestinal) respectively a decade ago (Turnbaugh et al., 2007; Human Microbiome Project, 2012a,b; Lozupone et al., 2012; http://metahit.eu/). HMP and MetaHIT generated unprecedented opportunities and datasets to test some of the most important ecological theories and laws for the first time in the world of human microbiome, arguably the closest ecosystem to the humans.

In the most important application field of SAR—the biogeography, significant advances have been made in the study of microbial biogeography during the past decade. The existence of biogeographic patterns of microorganisms has been firmly established, and the research focus is shifting to identifying the mechanisms that shape the discovered patterns (see excellent perspectives and reviews by Martiny et al., 2006; also see Peay et al., 2007; Fierer, 2008; van der Gast et al., 2008; Costello et al., 2012; Hanson et al., 2012; van der Gast, 2013, 2015; Barberán et al., 2014; Helmus et al., 2014). For example, regarding the patterns, the traditional view that “*everything is everywhere, but, the environment selects*,” suggested by Bass-Becking (1934) has been revised as “*Some things are everywhere and some things are not. Sometimes the environment selects and sometimes it doesn't*.” by van der Gast (2013, 2015). For another example, regarding the mechanisms, Hanson et al. (2012) proposed that selection, drift, dispersal and mutation govern the formation and maintenance of the microbial biogeographic patterns on ecological and evolutionary scales that are hardly separable.

Among those studies that established the microbial biogeography theory, the distance-decay relationship and accompanying SAR/STR patterns have certainly played a critical role. Nevertheless, the study on the biogeography of human microbiome is lagging behind the general microbial biogeography, although several pioneering studies have been conducted (Costello et al., 2009, 2012; Nasidze et al., 2009; Stearns et al., 2011; Ma et al., 2012; Zhou et al., 2013; Barberán et al., 2014; O'Doherty et al., 2014; Oh et al., 2014; Whiteson et al., 2014; Dickson et al., 2015). To the best of our knowledge, the within-body (intra-individual) SAR or DAR of the human microbiome from biogeography perspective has not been addressed yet. The present study is aimed at filling the current gap.

Specifically, we apply the recently extended DAR (Ma, 2017) to approach the within-body distribution of microbiome diversity—one of the most important aspects of the human microbiome biogeography. Compared with the traditional SAR approach, our approach has the following three unique features: (i) We adopted the Hill numbers, which are considered as the most appropriate metrics currently available for measuring the alpha diversity and for partitioning the beta diversity (Jost, 2007; Ellison, 2010; Chao et al., 2012, 2014) to assess and interpret the scaling of diversity with areas. The traditional SAR only studies the scaling of species richness with areas. (ii) The adoption of the Hill numbers allows us to investigate both alpha- and beta-diversity scaling with a unified approach. (iii) We establish the *DAR profile*, i.e., *z-q* pattern, where *z* is the diversity scaling parameter of DAR power law model, which is similar to the scaling parameter (z) of the traditional SAR but not limited to the scaling of species richness, and *q* is the diversity (Hill numbers) order, the *PDO profile* (*g-q* pattern), i.e., the pair-wise diversity overlap (PDO or *g*) at different diversity order (*q*), and the *MAD* profile (*D*_*max*_*-q* pattern), i.e., the maximal accrual diversity (MAD or *D*_*max*_) at different diversity order (*q*). We utilized to date the most comprehensive human microbiome distribution dataset, which sampled 18 major human microbiome sites covering the five primary human microbiome habitats or locations (i.e., airway, oral, gut, skin, and urological) of 242 individuals (www.hmpdacc.org) to draw the “maps” of the within-body biogeography of the human microbiome by establishing the previously described three profiles.

We expect that the findings from the present study should possess three important implications. First, the study demonstrated, for the first time, the within-body or intra-body biogeography of human bacterial diversity can be quantitatively described with DAR models and we further obtained the baseline parameters of the intra-DAR models. The baseline parameters refer to the DAR models constructed with the microbiome samples from healthy human individuals. If the samples are sufficiently large, the range or distribution of which should be rather stable. Second, it is expected than significant changes in host environment, such as the occurrence of human microbiome-associated diseases or dysbiosis, may significantly influence the intra-DAR parameters. Therefore, by monitoring the change of intra-DAR parameter, impacted by disease, can be helpful for personalized diagnosis and treatment assessment. Third, the intra-DAR approach demonstrated in this paper can be applied to other ecosystems or environments, for examples, the altitudinal scaling of biodiversity of Mount Everest or the underwater diversity scaling of ocean microbiome. Hopefully, monitoring the changes of DAR parameters in those environments/ecosystem may shed light on the environmental or ecosystem changes.

## Materials and methods

### Microbial species abundance data of the human microbiome

We use the 16s-rRNA datasets (V1-V3 region) from the NIH HMP (www.hmpdacc.org), a cross-sectional study that sampled 18 body sites distributed over five locations of 242 individuals. One mission of the HMP was to, for the first time in the human history, to collect and establish the “baselines” of human microbial communities (microbiotas) as well as the microbial genes they carry (i.e., human metagenome). The OTU (operational taxonomic unit) table, equivalent to the species abundance data of a community in macro ecology, was calculated from the 16s rRNA sequence data with 97% similarity cutoff *via* the QIIME software pipeline (Caporaso et al., 2010). We use the terms “OTU table,” “HMP dataset,” and “HMP cohort” interchangeably hereafter. It is noted that, among 242 individuals, we selected 150 individuals who were sampled at least in 5 microbiome sites to investigate their within-body DAR for obvious reason. It is also noted that the usage of the term “*species*” in this article is loose to be consistent with the usage in classic SAR (species area relationship). Obviously, in the context of microbiome diversity, OTU is a more appropriate term.

### Computational procedures for DAR analysis

The following *definitions* and *procedures* are adopted to design, perform and interpret intra-DAR analysis for the HMP datasets. Detailed descriptions on some of the procedures are provided in the online Supplementary Information to save page space.

### Definitions of alpha and beta diversities

The Hill's numbers (Hill, 1973) for measuring biodiversity were reintroduced into ecology by Jost (2007) and Chao et al. (2012) in recent years, and are defined as:


(1) $${}^{q}D={\left(\sum_{i=1}^{S}p_{i}^{q}\right)}^{1/(1-q)}$$

where *S* is the number of species, *p*_i_ is the relative abundance of species *i, q* is the order number of diversity. The parameter *q* determines the sensitivity of the Hill numbers to the relative frequencies of species abundances. When *q* = 0, the species abundances do not count at all and ^0^*D* = *S*, i.e., species richness. When *q* = 1, ^1^*D* is equal to the *exponential* of Shannon entropy, and is interpreted as the number of typical or common species in the community. When *q* = 2, ^2^*D* is equal to the reciprocal of Simpson index. In general, ^*q*^*D* (diversity of order *q*) is equivalent to the diversity of a community with *x* = ^*q*^*D* equally abundant species.

The Hill numbers have also been utilized to define beta diversity. Some recent advances (e.g., Jost, 2007; Ellison, 2010; Chao et al., 2012; Gotelli and Chao, 2013) have suggested that, compared with existing diversity indexes, Hill numbers are the most appropriate measure for alpha diversity, and the multiplicatively partitioned Hill numbers provide a better beta-diversity measure than most existing methods for partitioning and measuring beta-diversity. Beta diversity can be defined as:


(2) $${}^{q}D_{\beta }{=}^{q}D_{\gamma }{/}^{q}D_{\alpha }.$$

where ${(}^{q}D_{\alpha })$ and ${(}^{q}D_{\gamma })$are alpha diversity and gamma diversity (equivalent to alpha diversity of the meta-community), both measured in the Hill numbers. Obviously, Equation (2) is multiplicatively partitioned beta diversity. This beta diversity ${(}^{q}D_{\beta })$ derived from the above partition takes the value of *1* if all communities are identical, the value of *N* (the number of communities) when all the communities are completely different from each other (i.e., no shared species).

The unit of Hill numbers is *species equivalents* when applied to alpha and gamma diversities. When Hill numbers are used for measuring beta diversity, the unit is instead the number of distinct communities. With Jost (2007) words, the multiplicatively partitioned beta diversity measures “*the effective number of completely distinct communities*.” The exact formulae, which consider the pooling of local communities to form meta-community, to compute gamma and beta diversities, are provided in the online Supplementary Information.

A series of the Hill numbers corresponding to different diversity order *q* was defined as *diversity profile* (Jost, 2007; Chao et al., 2012). Ma (2017) extended the concept “*profile*” to describe other concepts/measures derived from the Hill numbers including *DAR profile, MAD (maximal accrual diversity) profile* and *PDO* (*pair-wise diversity overlap*) *profile*, all of which can be estimated for the HMP datasets later in this study. The three profiles together constitute our best efforts to construct the “map” of within-body or intra-individual biogeography of the human microbiome diversity.

### Fitting the DAR models and quantifying the DAR profiles

Ma (2017) postulated that Hill numbers should follow the same or similar pattern of SAR since all Hill numbers are in units of species (referred to as the effective number of species or as species equivalents in the literature), and further tested and suggested two models traditionally used in SAR studies for the DAR analysis.

The first DAR model is the traditional power law (PL) model, i.e.,


(3) $${}^{q}D=cA^{z},$$

where ^*q*^*D* represents for diversity measured in the *q*-*th* order Hill numbers, *A* for *area*, z is termed the scaling parameter or slope of the power law (as further explained below), *c* is a parameter that is strongly influenced by the choice of the first unit of area to accrue in DAR modeling. Theoretically, parameter *c* of PL is the number of species equivalents of diversity in one unit of area (^*q*^*D* = *cA*^*z*^ = *c*when *A* = 1), but not per unit of area since the scaling is nonlinear.

The second DAR model Ma (2017) suggested is the power law with exponential cutoff (PLEC), which was originally introduced to the modeling of SAR by Plotkin et al. (2000) and Ulrich and Buszko (2003), respectively (also see Tjørve, 2009). It has the form:


(4) $${}^{q}D=cA^{z}\exp(dA),$$

where *d* is a third parameter that should usually be negative in DAR modeling. The PLEC model is essentially an extension to parameter *c* of the PL, rather than to *z*, i.e., *c*(*x*) = *c* exp(*dx*), respectively. Therefore, *z* in PLEC is assumed to be with the similar interpretation as in the basic PL model (Equation 3). The newly added item exp(*dA*) can cause the exponential decay, which eventually overwhelms the seemingly unlimited growth of PL at very large value of *A*.

The log-linear transformation below can transform the fitting of non-linear Equations (3,4) into simple linear regressions (Equations 5, 6):


(5) $$\begin{array}{rcl} \ln\;\left(D\right) & = & \ln\;\left(c\right)+z\ln\;\left(A\right) \end{array}$$

(6) $$\begin{array}{rcl} \ln\;\left(D\right) & = & \ln\;\left(c\right)+z\ln\;\left(A\right)+dA \end{array}$$

Similar to the interpretation of *z* in the traditional SAR, the *z* of PL-DAR equals the ratio of *diversity accrual rate* to *area increase rate*. When PL is fitted with the above log-transformed linear regression, *z* is the *slope* or *tangent* of the PL model. Nevertheless, if the PL is directly fitted with non-linear optimization such as Marquardt's algorithm or Simplex method (Ma, 1992), the slope or tangent of the PL is reliant on both *z* and *c*. This is an advantage of fitting the DAR models with the above log-linear transformation, besides being computationally simple.

To actually fit the above models to the HMP datasets, besides adopting the linear-transformation above, there are two remaining issues. One is the accrual of areas, i.e., the accrual of the 18 microbiome sites in the case of HMP datasets, and another is the accrual of the corresponding diversity (Hill numbers). We further describe the accrual schemes below to complete the computational procedures for constructing the DAR models for the within-body (intra-individual) biogeography.

As explained previously, the sequence (order) of accruing areas may strongly affect the estimation of parameter *c*. To remedy the random noise from arbitrarily setting the accrual order of areas, we (i) randomly permutate the orders of 18 human microbiome sites for within-body DAR modeling, (ii) randomly select 100 orders from the total permutations, (iii) fit the DAR models (PL & PLEC) to each of the 100 selected orders and obtain 100 set of DAR models, and (*iv*) take the average parameters of 100 times of re-sampling from the total permutations as the DAR model for an individual in the HMP cohort. Note that the 100 times of re-sampling should also help to deal with issues in diversity estimations such as discussed in Haegeman et al. (2013, 2014) and Chiu and Chao (2015).

The accrual of diversity is more complex than the accrual of species in traditional SAR since there may be more than one way to accrue diversity. Ma (2017) summarized three principles to uniquely define the accrual scheme for diversity accrual. The first principle is to use the Hill numbers, or what Jost (2007) called the *true* diversity; the second is to follow the essential idea of SAR, as captured by the word “accumulation” or “aggregate,” i.e., diversity (the Hill numbers or species equivalents) are accumulated for the accrued areas; the third is that the diversity scaling model should be useful for *predicting* diversity at different levels of areas accumulated. These three principles are the essential axioms to follow for extending the SAR to DAR. A detailed description on implementing the three principles for diversity accrual is provided in the section of “the scheme to accrue diversity” of the online Supplementary Information.

Inspired by the concept of *diversity profile* (Chao et al., 2012, 2014), Ma (2017) defined the relationship between DAR model parameter (*z*) of the traditional PL-DAR model and the diversity order (*q*), or *z-q* pattern (trend), as the *DAR profile*. We will quantify the DAR profile for the within-body DAR profiles of the human microbiome with the HMP datasets mentioned previously in the results and discussion section.

### Quantifying the PDO (pair-wise diversity overlap) profiles

Inspired by Tjørve and Tjørve's (2008) work on SAR based on the self-similarity principle, Ma (2017) derived the *pair-wise diversity overlap* (*g*) (PDO) of two bordering areas (A & 2A) of the same size as:


(7) $$\begin{array}{r} g=\left(2D_{A}-D_{2A}\right)/D_{A}=2-2^{z} \end{array}$$

where *z* is the scaling parameter of the PL-DAR model, *D*_*A*_ and *D*_2*A*_ are the diversity of two bordering areas respectively but they are not needed to estimate the PDO as indicated obviously in Equation (7). When *z* = 1, then *g* = 0, there is no overlap; and when *z* = 0, *g* = 1, there is total overlap. In reality, *g* should usually be between 0 and 1. The PDO is essentially the proportion of the new diversity in the second area of the pair of two bordering areas, and it is therefore also a similarity measure of a pair of bordering areas. Similar to previous DAR profile, the pair-wise diversity overlap (PDO) profile, i.e., *g*-*q* pattern (trend) or the PDO (*g*) at different diversity order (*q*) can be quantified for the within-body biogeography of the human microbiome diversity as shown in the results and discussion section. In this case, PDO profile can be harnessed to measure the similarity between two bordering sites in the microbiome diversity within a human body.

### Quantifying the MAD (maximal accrual diversity) profiles

Similar to the role of SAR model in the global biodiversity conservation, we expect the DAR models built in this study will find biomedical applications in studying the within-body baseline of the human microbiome diversity scaling.

Ma (2017) derived the maximal accrual diversity (MAD) based on the PLEC DAR model: that is, when

(8) $$\begin{array}{r} A_{\max}=-z/d\left(\text{z}>0,\text{d}<0\right) \end{array}$$

^*q*^*D* may have a maximum in the following form:


(9) $$D_{\max}=Max{(}^{q}D)=c{\left(-\frac{z}{d}\right)}^{{}^{z}}\exp(-z)=cA_{\max}^{z}\exp(-z)$$

Equations (8, 9) can be used to estimate the theoretical MAD of the human microbiome, whether it is alpha- or beta-diversity. Similar to the DAR profile and PDO profile introduced previously, the relationship between the *D*_*max*_ and diversity order (*q*), i.e., *D*_*max*_-*q* pattern (trend) is then termed the MAD profile, which will be quantified for the within-body biogeography of the human microbiome diversity later in this article.

Note that when *z* < 0 and *d* > 0, the extreme value is a local minimum rather than maximum. The principles and measures to deal with such complications are further discussed in the section of “Signs of DAR parameters” in the online Supplementary Information.

### Statistical distribution of DAR/PDO/MAD profile parameters

We analyzed the statistical distributions of the intra-DAR scaling parameters by fitting two contrastingly different statistical distributions: the *normal distribution* and *power-law distribution* (Clauset et al., 2009; Gotelli and Ellison, 2013). The former describes a largely symmetric distribution of the scaling parameters across individuals, and the latter describes an asymmetric (long-tail) probability distribution that has some unique properties not possessed by the normal distribution. This analysis was motivated to shed light on the nature of individual heterogeneity (personal difference) in microbiome.

Since the information on the normal distribution can be readily found in standard statistics textbook (e.g., Gotelli and Ellison, 2013), we only list some basic information about the power law distribution below. Power law distribution has a probability density function as follows:


(10) $$\begin{array}{r} p\left(x\right)=\frac{K-1}{x_{\min}}{\left(\frac{x}{x_{\min}}\right)}^{-K} \end{array}$$

where *x* is the random variable (i.e., the DAR/PDO/MAD profile parameters in this study), *x*_*min*_ is the minimum value of *x*, and *K* is the exponent of the power law distribution, which has rich information about heterogeneity of the distribution. A comprehensive discussion on the power law distribution, including its fitting to data, can be found in Clauset et al. (2009).

## Results and discussion

We investigate the within-body (intra-individual) biogeography of the human microbiome by fitting two selected DAR models (PL and PLEC) to each of the 150 individuals in the HMP cohort, for both alpha- and beta-diversity scaling respectively. We further established the intra-DAR profile (*z-q* pattern), the intra-PDO profile (*g-q* pattern), and the intra-MAD profile (*D*_*max*_-*q* pattern) again for both alpha- and beta-diversity scaling, respectively. The *intra-* prefix is omitted hereafter when there is not ambiguity (i.e., not to be confused with the inter-individual DAR or inter-DAR analyses for short, which are discussed elsewhere but may be compared with the intra-DAR below occasionally). In addition, we use the terms “within-body” and “intra-individual” interchangeably, although the formal is a more accurate description for what we study, but the latter is more intuitive in the context of comparing with the inter-individual DAR.

### The performance of DAR models (PL-DAR and PLEC-DAR)

The DAR models were constructed by accruing diversities across all the sites (up to 18) of an individual sampled in the HMP cohort. The intra-DAR model hence reflects the biogeography of the human microbiome within a human body, rather than across individuals within a human population (cohort) as discussed elsewhere (Ma, 2017 submitted). Furthermore, we distinguish between the alpha and beta version of DAR, i.e., alpha-DAR and beta-DAR. The results, a pair of PL & PLEC models for alpha-DAR and beta-DAR, respectively, for each individual in the HMP cohort, respectively, are listed in Tables 1, 2 (brief version) and Tables S1, S2 (full version), included in the online Supplementary Information that also contains the full results of the statistical distribution testing for the major parameters of the DAR models in Tables S1, S2.

**Table 1:** The intra-individual alpha-DAR modeling for the HMP dataset (Demon version, Full version in Table S1).

**Order**	**Subject number**	**Power law (PL)**						**PL with exponential cutoff (PLEC)**							
		***z***	**ln(*c*)**	***R***	***p*-value**	***g***	N*	***z***	***d***	**ln(*c*)**	***R***	***p*-value**	N*	***A_max_***	***D_max_***
*q = 0*	132902142	1.061	6.170	0.947	0.000	−0.162	100	1.896	−0.213	6.077	0.979	0.000	100	9	4146.7
	147406386	0.776	6.723	0.988	0.000	0.282	100	1.020	−0.045	6.632	0.996	0.000	100	23	6552.0
	158013734	0.827	6.359	0.977	0.000	0.213	100	1.167	−0.057	6.205	0.990	0.000	100	20	5200.5
	158114885	0.776	6.641	0.974	0.000	0.271	100	1.120	−0.055	6.471	0.989	0.000	100	20	6124.7
	158155345	0.730	6.742	0.992	0.000	0.340	100	0.909	−0.033	6.676	0.997	0.000	100	27	6474.3
	^**^…														
	**Mean**	0.860	6.473	0.972	0.000	0.151	100	1.301	−0.093	6.346	0.989	0.000	100.	19	6068.2
	**Std. Err**.	0.068	0.128	0.009	0.000	0.106	0	0.201	0.040	0.126	0.004	0.000	0	0	145.8
*q = 1*	132902142	0.930	4.704	0.900	0.001	0.019	100	1.994	−0.271	4.585	0.960	0.001	100	7	715.5
	147406386	0.617	5.058	0.892	0.001	0.440	100	1.121	−0.094	4.871	0.950	0.000	100	12	686.6
	158013734	0.704	4.944	0.910	0.000	0.343	100	1.237	−0.090	4.701	0.956	0.000	100	14	820.7
	158114885	0.633	5.099	0.822	0.002	0.400	96	1.193	−0.090	4.820	0.903	0.001	96	13	820.8
	158155345	0.523	5.267	0.869	0.001	0.539	98	0.854	−0.063	5.159	0.923	0.001	100	14	685.3
	^**^…														
	**Mean**	0.721	4.951	0.881	0.001	0.301	99	1.386	−0.136	4.744	0.942	0.001	99	12	790.7
	**Std. Err**.	0.072	0.089	0.020	0.000	0.096	1	0.204	0.045	0.064	0.013	0.000	1	0	26.6
*q = 2*	132902142	0.950	3.053	0.867	0.004	0.012	75	2.009	−0.273	2.965	0.943	0.003	76	7	143.4
	147406386	0.526	3.141	0.738	0.008	0.526	54	1.218	−0.157	3.244	0.848	0.004	79	8	91.6
	158013734	0.685	3.675	0.913	0.000	0.369	93	1.111	−0.073	3.491	0.950	0.001	94	15	223.8
	158114885	0.566	3.299	0.795	0.004	0.454	67	0.995	−0.084	3.296	0.867	0.003	77	12	116.1
	158155345	0.645	3.112	0.836	0.005	0.399	45	0.823	−0.072	3.453	0.862	0.007	63	11	102.7
	^**^…														
	**Mean**	0.682	3.292	0.828	0.004	0.340	72	1.333	−0.147	3.249	0.902	0.003	82	10	158.0
	**Std. Err**.	0.096	0.138	0.039	0.002	0.114	8	0.230	0.046	0.109	0.026	0.001	4	0	9.3
*q = 3*	132902142	0.903	2.346	0.883	0.004	0.102	63	1.675	−0.217	2.422	0.936	0.003	70	8	64.8
	147406386	0.269	2.876	0.699	0.013	0.740	52	1.033	−0.154	2.807	0.821	0.007	80	7	42.1
	158013734	0.670	3.178	0.929	0.000	0.390	93	0.951	−0.049	3.072	0.947	0.001	95	19	139.2
	158114885	0.429	2.874	0.790	0.005	0.588	70	0.757	−0.064	2.853	0.859	0.004	79	12	52.7
	158155345	0.403	2.955	0.790	0.009	0.613	49	0.696	−0.070	3.000	0.853	0.007	63	10	49.6
	^**^…														
	**Mean**	0.568	2.818	0.825	0.006	0.455	70	1.104	−0.121	2.789	0.891	0.004	81	12	81.5
	**Std. Err**.	0.139	0.173	0.051	0.003	0.138	9	0.199	0.039	0.135	0.030	0.001	5	2	5.0

** *Only the alpha-DAR models for five individuals are listed here to save page space, and the full results are presented in Table S1*.

**Table 2:** The intra-individual beta-DAR modeling for the HMP dataset (Demon version, Full version in Table S2).

**Order**	**Subject number**	**Power law (PL)**						**PL with exponential cutoff (PLEC)**							
		***z***	**ln(*c*)**	***R***	***p*-value**	***g***	N*	***z***	***d***	**ln(*c*)**	***R***	***p*-value**	N*	***A _max_***	***D_max_***
*q = 0*	132902142	0.731	0.168	0.987	0.000	0.338	100	1.060	−0.066	0.012	0.993	0.000	100	16	6.6
	147406386	0.721	0.184	0.994	0.000	0.351	100	0.908	−0.028	0.051	0.997	0.000	100	32	9.9
	158013734	0.714	0.227	0.992	0.000	0.360	100	0.940	−0.031	0.051	0.997	0.000	100	30	10.1
	158114885	0.681	0.211	0.991	0.000	0.396	100	0.886	−0.027	0.044	0.996	0.000	100	33	9.4
	158155345	0.684	0.203	0.993	0.000	0.393	100	0.898	−0.033	0.052	0.997	0.000	100	28	8.5
	^**^…														
	**Mean**	0.733	0.192	0.992	0.000	0.337	100	0.964	−0.038	0.041	0.997	0.001	99	28	9.6
	**Std. Err**.	0.004	0.003	0.000	0.000	0.004	0	0.007	0.002	0.002	0.000	0.000	0	1	0.1
*q = 1*	132902142	0.617	0.239	0.956	0.000	0.461	100	1.136	−0.104	−0.007	0.978	0.000	100	11	4.8
	147406386	0.544	0.314	0.970	0.000	0.540	100	0.886	−0.052	0.073	0.987	0.000	100	17	5.5
	158013734	0.594	0.319	0.972	0.000	0.488	100	0.939	−0.048	0.050	0.988	0.000	100	20	6.8
	158114885	0.528	0.341	0.961	0.000	0.556	100	0.891	−0.048	0.045	0.985	0.000	100	18	5.8
	158155345	0.546	0.310	0.967	0.000	0.537	100	0.897	−0.053	0.062	0.986	0.000	100	17	5.5
	^**^…														
	**Mean**	0.594	0.305	0.963	0.001	0.486	99	1.024	−0.073	0.032	0.985	0.001	98	16	6.1
	**Std. Err**.	0.006	0.006	0.001	0.000	0.007	1	0.019	0.006	0.005	0.001	0.000	1	0	0.1
*q = 2*	132902142	0.733	0.156	0.967	0.000	0.333	100	1.183	−0.090	−0.057	0.983	0.000	100	13	6.1
	147406386	0.397	0.489	0.829	0.002	0.680	100	0.964	−0.086	0.088	0.914	0.001	99	11	4.3
	158013734	0.671	0.263	0.968	0.000	0.404	100	1.021	−0.048	−0.010	0.982	0.000	100	21	8.0
	158114885	0.532	0.429	0.899	0.000	0.551	100	1.062	−0.070	−0.003	0.948	0.000	100	15	6.2
	158155345	0.631	0.317	0.933	0.000	0.446	100	1.184	−0.084	−0.072	0.968	0.000	100	14	6.6
	^**^…														
	**Mean**	0.612	0.315	0.920	0.001	0.460	98	1.151	−0.088	−0.038	0.960	0.001	97	15	6.8
	**Std. Err**.	0.010	0.012	0.004	0.000	0.011	1	0.018	0.005	0.007	0.002	0.000	1	1	0.3
*q = 3*	132902142	0.809	0.109	0.970	0.000	0.243	100	1.180	−0.074	−0.066	0.982	0.001	100	16	7.5
	147406386	0.380	0.538	0.772	0.006	0.694	100	1.058	−0.101	0.051	0.895	0.002	94	10	4.4
	158013734	0.732	0.216	0.973	0.000	0.335	100	1.055	−0.045	−0.036	0.983	0.000	100	24	9.4
	158114885	0.533	0.471	0.868	0.000	0.550	100	1.130	−0.079	−0.016	0.929	0.000	100	14	6.4
	158155345	0.649	0.337	0.911	0.000	0.427	100	1.317	−0.101	−0.134	0.956	0.000	100	13	6.9
	^**^…														
	**Mean**	0.635	0.311	0.902	0.002	0.433	97	1.207	−0.093	−0.064	0.948	0.002	97	17	7.8
	**Std. Err**.	0.012	0.014	0.006	0.000	0.014	1	0.019	0.005	0.008	0.003	0.000	1	1	0.4

** *Only the beta-DAR models for five individuals are listed here to save page space, and the full results are presented in Table S2*.

In Tables 1, 2 and Tables S1, S2, listed are the columns of diversity order (*q*), subject number (in the HMP cohort), the parameters of the PL model and PLEC respectively. Listed parameters of the PL model include *z* (scaling parameter), ln(*c*), *R* (correlation coefficient), *p*-value, *g* (pair-wise diversity overlap), and N^*^ (the number of successful fittings). Similarly, listed parameters of the PLEC model include *z* (scaling parameter), ln(*c*), *d* (exponential cutoff parameter), *R, p*-value, N^*^, A_max_ (the number of accrued individuals corresponding to MAD), and *D*_max_ (the maximal accrual diversity, i.e., MAD). We use three parameters (*R, p*-value, and N^*^) to judge the performance or goodness-of-fitting of DAR models to the HMP dataset. Note that N^*^ is the number of successful fittings out of maximal 100 times of re-sampling from the randomly permutated orders of the 18 sites within an individual. We built one DAR model for each of the randomly permutated order and took the averages of the parameters from 100 times of re-sampling. Therefore, we consider some failures are tolerable as long as we can get the average parameters with large sample (>50). Since the model fitting we used is linear regression, either *R* or *p*-value alone is sufficient to determine the success of failure. Although the average *p*-value in Tables 1, 2 does not reflect goodness of individual model-fitting, it does demonstrate the performance of a model to the population. As shown in Tables 1, 2, the average *p*-values are < 0.01, which demonstrate the fine suitability of both the PL and PLEC models to the intra-DAR analysis of the HMP datasets.

In summary, from pure statistical fitting, the PLEC model fitted to the datasets slightly better than the PL model. From ecological perspective, PL model is simple but with established ecological interpretations inherited from SAR (species area relationship), and PLEC has an advantage of predicting maximal accrual diversity (MAD). Both the models are complementary to each other in our DAR analysis: the PL model is harnessed to establish DAR profiles (*z-q* pattern), and PDO profiles (*g-q*), and the PLEC model to establish MAD profiles (*D*_max_-*q*).

### The DAR profiles of 150 individuals in the HMP cohort

**Figure 1:**
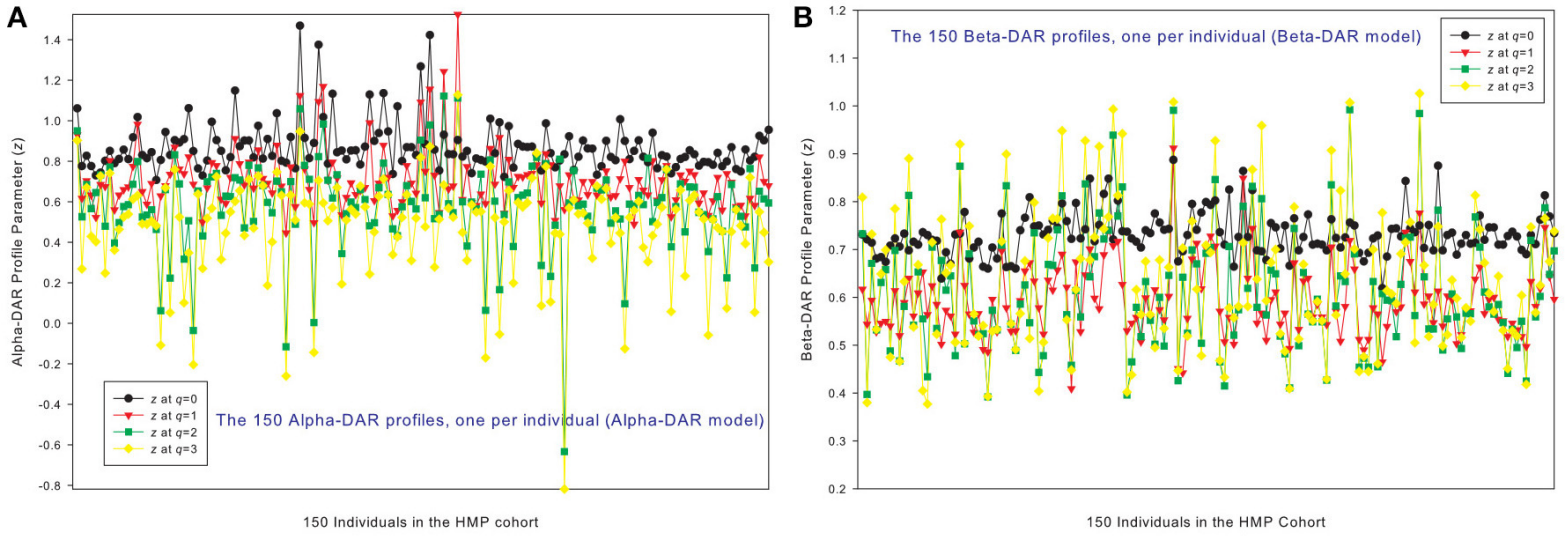
The alpha-DAR profiles **(A)** and beta-DAR profiles **(B)** of 150 individuals in the HMP cohort: x-axis should be the subject IDs of the individuals but omitted to avoid overcrowded labeling, and y-axis is the DAR profile parameter *z* at four different diversity orders (*q* = 0–3). Therefore, every four points corresponding to each ID on x-axis is the DAR profile of that specific individual, and the four curves constitute the DAR profiles of 150 the individuals in the HMP cohort. Inter-individual heterogeneity in the DAR profile is also obvious, which is further described with the power law statistical distribution (see Table 4, Figure 6).

**Figure 2:**
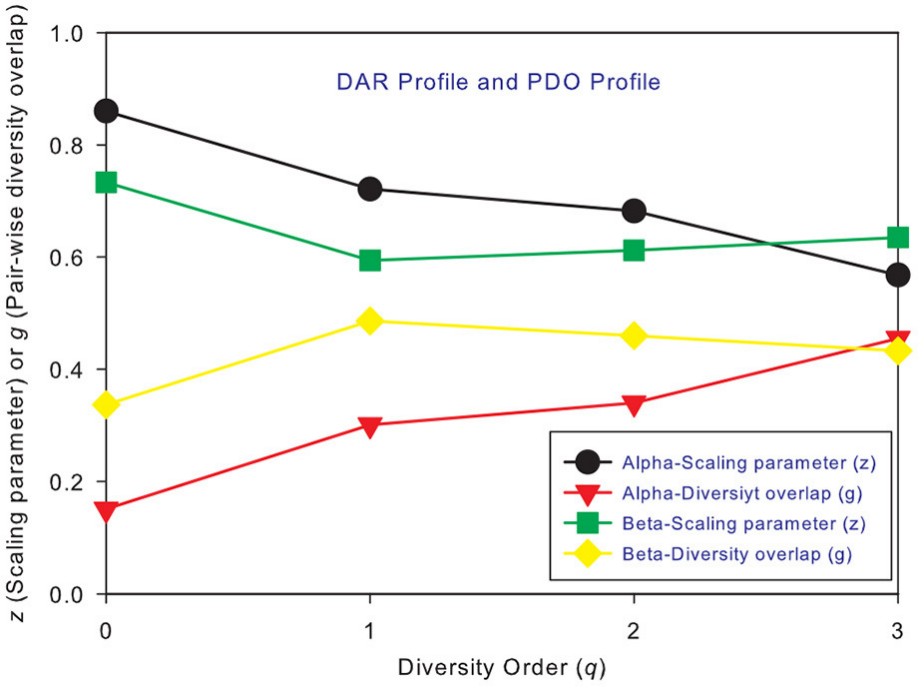
The intra-DAR profile and intra-PDO profile for the alpha- and beta-diversity scaling, respectively: (i) the alpha-DAR profile monotonically decreases with *q*, and its counterpart PDO profile monotonically increases with *q*; (ii) the beta-DAR profile is valley-shaped, and its counterpart PDO profile is mountain-shaped; (iii) the difference between alpha and beta profiles seems largest at diversity order *q* = 0–1, and smallest at *q* = 3.

Figures 1A,B show the alpha-DAR and beta-DAR profiles, respectively, of 150 individuals in the HMP cohort. The apparent irregularity in the *z*-values of the DAR profiles is primarily due to the strong variability (heterogeneity) among individuals. When *z* is averaged across individuals, the heterogeneity is hidden as shown in Figure 2, but the trend of average DAR profiles is obvious in Figure 2. The average DAR profile alpha-*z* is monotonically decreasing (0.860, 0.721, 0.682, 0.568) for *q* = 0–3, and average beta-*z* is (0.733, 0.594, 0.612, 0.635) for *q* = 0–3, respectively. As to the inter-individual heterogeneity in *z* of the DAR profiles, we postpone its discussion to a later discussion after we analyzed all three types of the profiles.

### The PDO profiles of 150 individuals in the HMP cohort

**Figure 3:**
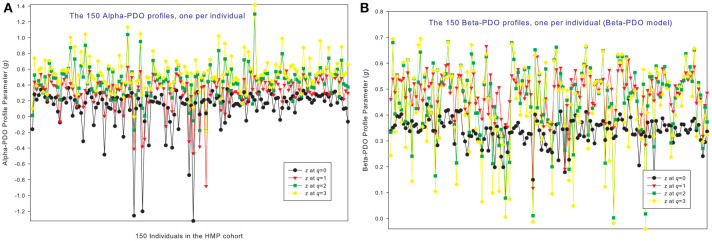
The alpha-PDO profiles **(A)** and beta-PDO profiles **(B)** of 150 individuals in the HMP cohort: x-axis should be the subject ID of individuals but omitted to avoid overcrowded labeling, and y-axis is the PDO profile parameter (*g*) at four different diversity orders (*q* = 0–3). Therefore, the four points corresponding to each subject ID on the x-axis is the PDO profile of that specific individual, and the four curves constitute the PDO profiles of 150 individuals in the cohort. Inter-individual heterogeneity in the PDO profile is also obvious, which is further described with the power law statistical distribution (see Table 4, Figure 6).

Figures 3A,B shows the alpha-PDO profiles and beta-PDO profiles respectively, of 150 individuals. Similar to the DAR profiles of the 150 individuals, the PDO profiles show strong inter-individual heterogeneity, which we further discuss in a later section. By taking the average *g* (PDO) of 150 individuals, the trend of PDO profile becomes clear as shown in Figure 2. The average alpha-PDO profile is monotonically increasing [alpha-*g* = (0.151, 0.301, 0.340, 0.455)], and the average beta-PDO profile is mountain-shaped [beta-*g* = (0.337, 0.486, 0.460,0.433)]. Figure 2 also reveals another interesting observation, that is, the patterns of DAR profile (*z*-*q*) and PDO profile (*g*-*q*) are reciprocal. For example, while alpha-DAR profile is monotonically decreasing, alpha-PDO profile is monotonically increasing.

### The MAD profiles of 150 individuals in the HMP cohort

**Figure 4:**
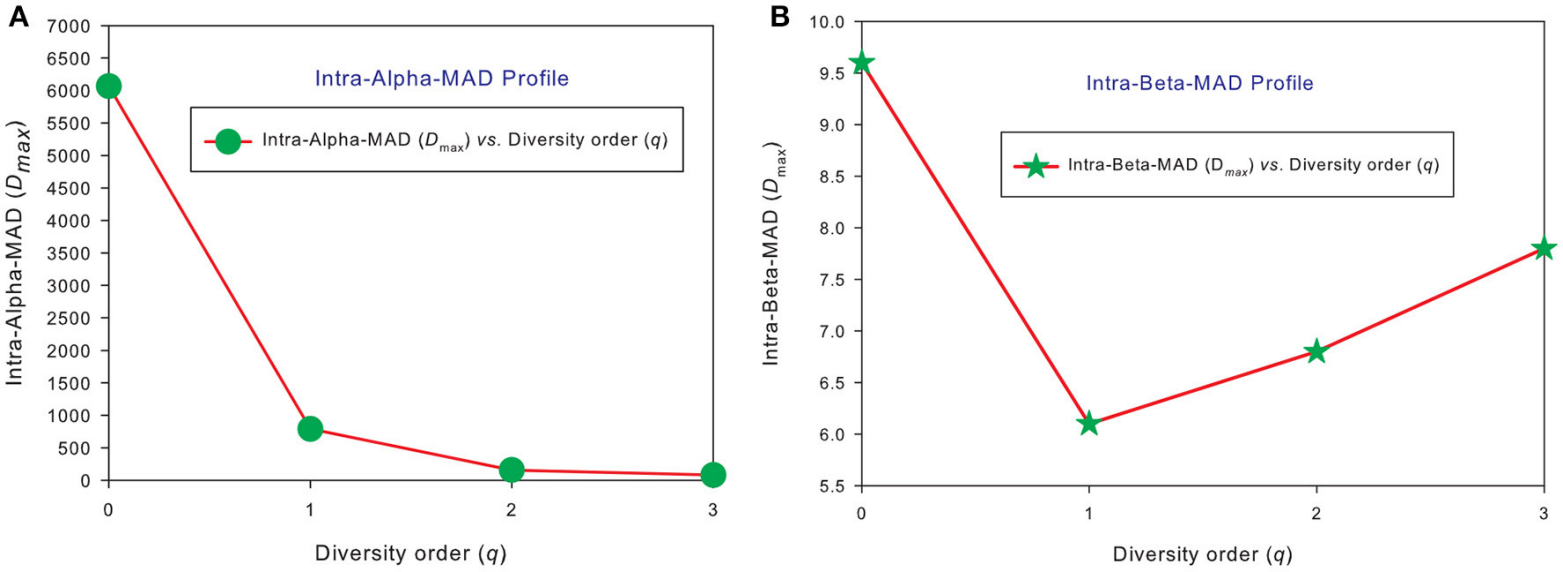
The *average* MAD profiles of 150 individuals in the HMP cohort: **(A)** for alpha-MAD monotonically decreases with diversity order (*q*), and **(B)** for beta-MAD is valley-shaped. The average *D*_max_ is calculated from the *D*_*max*_ values of 150 MAD profiles for each diversity order *q* (the x-axis).

**Figure 5:**
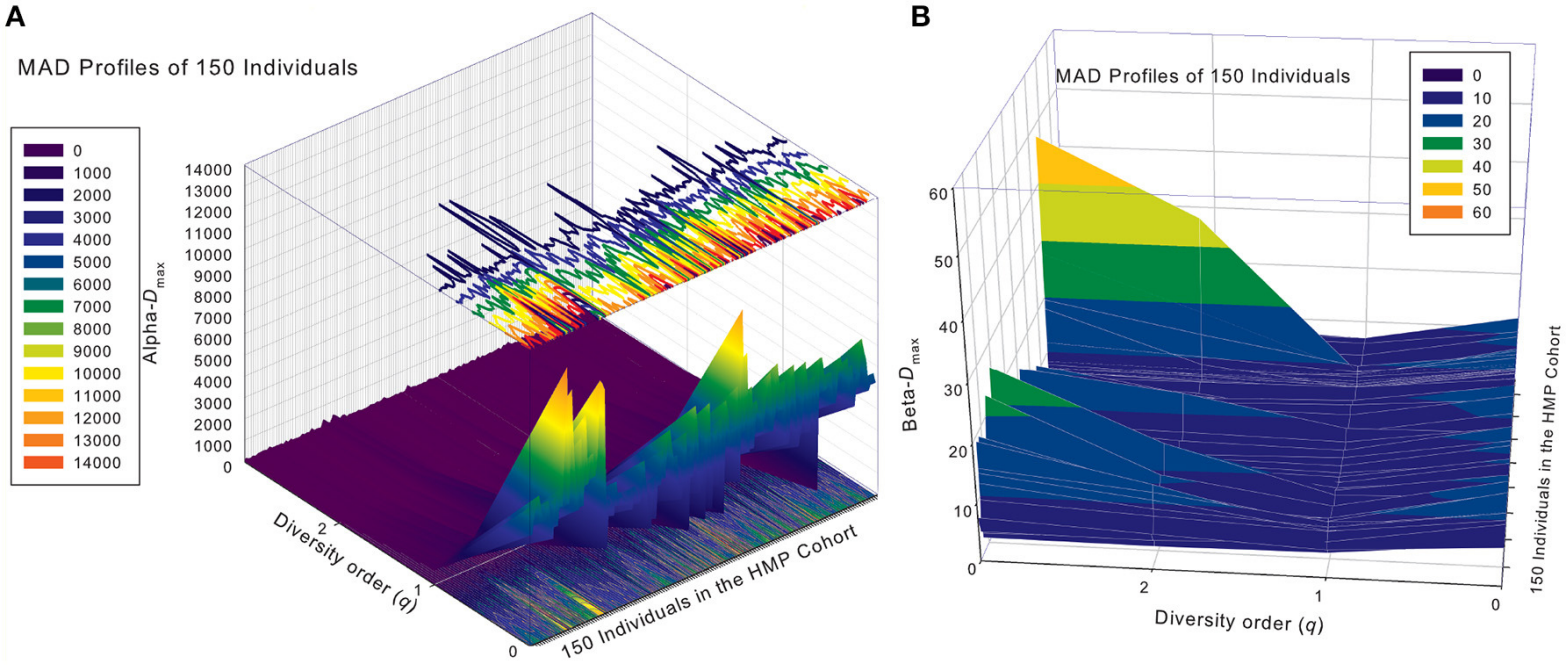
The alpha-MAD profiles **(A)** and beta-MAD profiles **(B)** of 150 individuals in the HMP cohort: *x*-axis should be the subject ID of the individuals in the HMP cohort but omitted to avoid overcrowded labeling, *y*-axis is the diversity order (*q* = 0–3), and *z*-axis is the alpha- or beta-*D*_max_ corresponding to each diversity order (*q*: *y*-axis) of each individual (*x*-axis). In the case of alpha-MAD **(A)**, along the diversity order (*q*) axis, each individual has a monotonically decreasing curve (fold lines). In contrast, in the case of beta-MAD **(B)**, the fold lines are valley-shaped.

Similar to DAR profiles and PDO profiles, we used the average *D*_max_ of 150 individuals (MAD profiles) to demonstrate the general pattern of MAD profile. The alpha-MAD is monotonically decreasing [alpha-*D*_max_ = (6068, 790.7, 158.0, 81.5)] (Figure 4A) and beta-MAD is valley-shaped [beta-*D*_max_ = (9.6, 6.1, 6.8, 7.8)] (Figure 4B). We further illustrate the MAD profiles of 150 individuals in Figure 5A (alpha-MAD profiles) and Figure 5B (beta-MAD profiles).

### The inter-individual heterogeneity of the Intra-DAR, Intra-PDO, and Intra-MAD profiles

In previous sections, we established DAR, PDO and MAD profiles for each of the 150 individuals in the HMP cohort. Here we address a follow-up question about those profiles, that is, are individuals have the same or similar intra-individual (or within-body) DAR, PDO and MAD profiles? This question is of both important theoretical and practical significance. Theoretically, the variability or heterogeneity of the intra-DAR/PDO/MAD parameters (*z, g, D*_*max*_) reflects the evolutionary and ecological properties of the microbiome diversity distribution within a human body. Practically, the heterogeneity may reflect the inherent difference among individual differences due to genetic and/or environmental backgrounds. The differences may have important clinical implications for the personalized diagnosis and treatments of the so-termed microbiome-associated diseases.

The approach we used to assess the inter-individual heterogeneity is to fit the two contrastingly difference statistical distributions in terms of the skewness, the *normal* distribution and *power law* distribution. We are particularly interested in skewness because it can reveal the nature of cohort heterogeneity. The normal distribution has zero skewness, and is symmetrical. The power law distribution has long tail is highly skewed.

**Figure 6:**
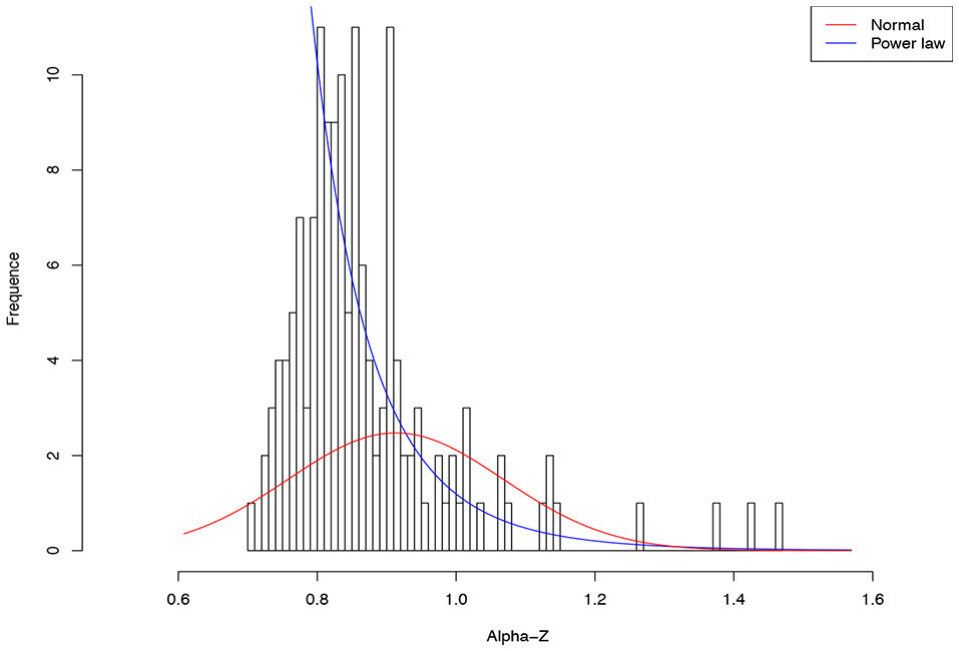
Fitting the power law statistical distribution (Equation 10) and normal distribution to the alpha-DAR scaling parameter *z* (at diversity order *q* = 0) of 150 individuals in the HMP cohort: the power law distribution successfully fitted to *z-*values (*p* = 0.999), but the normal distribution failed to fit (*p* = 0.000) (also see Table 4).

As shown in Table 4, among 64 test cases, the normal distribution succeeded only in four cases of ln(*c*), and one case in beta-*D*_max_. As interpreted previously, ln(*c*) has limited ecological significance and is largely due to random sampling effect. In contrast, only five cases failed to fit the power law distribution, and all of the five failures occurred in the cases of parameter *d* of the PLEC-DAR model. Parameter *d* (exponential cutoff parameter) of PLEC is the usually a rather small and of little differences among individuals (see Tables S1, S2). This may explain its failure to fit to the power law distribution. Therefore, majority (92%) of the DAR/PDO/MAD parameters satisfied with the power law distribution. Figure 6 shows one example of fitting the power law distribution to the alpha-DAR scaling parameter *z* at *q* = 0, and the highly skewed, long-tail feature is obvious. The poor fitting of the normal distribution to the same data is also displayed in Figure 6.

The wide suitability of the power law distribution indicates that most of the intra-DAR parameters are highly heterogeneous among individuals and the heterogeneity is highly skewed. The so-called “no average” property of the power law distribution implies that there is not an “average Joe” in a population (or cohort) that can represent the population (cohort). In other words, the average of a population is a rather poor representative of the majority in the population. The high-skewness, long-tail property predicts that in the cohort, most individuals should have rather small scaling parameter (long tail) values, while a handful of individuals may have disproportionally large values. If microbiome data follow the power law distribution, rather than the normal distribution, many statistical methods that assume the normal distribution should not be applied to analyze the data to ensure the validity of statistical analysis. We argue that the message from the power law distribution is critical for understanding the biogeography of the human microbiome, which may also imply that personalized medicine is not only necessary but also challenging for microbiome-associated diseases because the lack of an “average Joe.”

## Discussion

In a previous study, we investigate the inter-individual DAR with the same HMP datasets by building the DAR models for each of the 18 microbiome sites across individuals in the HMP cohort (Ma, in revision). Table 3 summarized the DAR- PDO- and MAD profile parameters of both the inter-DAR and intra-DAR analyses. To simplify the comparison, we used average parameters in both the inter-DAR and intra-DAR results. The inter-DAR averages were from averaging the parameters of 18 sites, and the intra-DAR averages were form the 150 individuals in the cohort. From Table 3, we can see that the patterns of intra-DAR/PDO/MAD are similar with their inter-DAR/PDO/MAD counterparts. However, there are differences, which we summarize below.

**Table 3:** Summary of the DAR profile (*z-q*), PDO profile (*g-q*) and MAD profile (*D*_max_-*q*) of both inter-DAR^*^ and intra-DAR^**^ analyses.

**Profile definitions**			***q* = 0**	***q* = 1**	***q* = 2**	***q* = 3**	**Pattern description**
DAR Profile (*z-q*)	Alpha	Inter-DAR^*^	0.502	0.253	0.154	0.121	Monotonically decreasing
		Intra-DAR	0.860	0.721	0.682	0.568	Monotonically decreasing
	Beta	Inter-DAR	0.445	0.232	0.229	0.283	Valley-shaped
		Intra-DAR	0.733	0.594	0.612	0.635	Valley-shaped
PDO Profile (*g-q*)	Alpha	Inter-DAR	0.580	0.805	0.882	0.907	Monotonically increasing
		Intra-DAR	0.151	0.301	0.340	0.455	Monotonically increasing
	Beta	Inter-DAR	0.637	0.824	0.824	0.774	Mountain-shaped
		Intra-DAR	0.337	0.486	0.460	0.433	Mountain-shaped
MAD Profile (*D_*max*_-q*)	Alpha	Inter-DAR	11990.1	876.5	175.3	87.6	Monotonically decreasing
		Intra-DAR	6068	790.7	158.0	81.5	Monotonically decreasing
	Beta	Inter-DAR	17.8	7.1	8.2	11.2	Valley-shaped
		Intra-DAR	9.6	6.1	6.8	7.8	Valley-shaped

* *The inter-DAR results are obtained from Ma (2017), and the parameters are based on the average of 18 sites (each site has a set of inter-individual DAR models)*.

** *The intra-DAR parameters are based on the averages of 150 intra-individual (within-body) DAR models (one set of model for each individual in the HMP cohort)*.

**Table 4:** The distribution fitting results for the parameters of PL and PLEC models of the intra-subject-DAR.

**Diversity order (** ***q*** **) and fitted distribution**			**PL-DAR**			**PLEC-DAR**				
			***z***	**ln(*c*)**	***g***	***z***	***d***	**ln(*c*)**	***A_*max*_***	***D_*max*_***
*q = 0*	*Alpha-*DAR	Normal (*p*-value)	0.000	0.000	0.000	0.000	0.000	0.000	0.010	0.000
		Power law (*p*-value)	0.999	1.000	1.000	0.784	NA	0.042	0.641	0.989
		Power law (*K*)	9.649	38.074	31.169	5.778	NA	21.473	10.023	7.353
	*Beta-*DAR	Normal (*p*-value)	0.000	0.000	0.000	0.000	0.000	0.000	0.000	0.002
		Power law (*p*-value)	0.978	0.888	0.407	0.145	NA	0.908	0.694	0.749
		Power law (*K*)	20.810	13.540	13.401	18.730	NA	6.247	10.014	9.598
*q = 1*	*Alpha-*DAR	Normal (*p*-value)	0.000	0.000	0.000	0.000	0.000	0.000	0.000	0.000
		Power law (*p*-value)	0.991	1.000	0.581	0.950	0.820	1.000	0.422	NA
		Power law (*K*)	7.523	37.834	7.890	6.167	1.796	38.271	8.963	NA
	*Beta-*DAR	Normal (*p*-value)	0.000	0.000	0.000	0.000	0.000	0.000	0.000	0.248
		Power law (*p*-value)	0.832	0.140	0.179	0.211	NA	0.883	0.871	0.868
		Power law (*K*)	10.303	9.004	13.274	9.182	NA	5.200	11.043	9.199
*q = 2*	*Alpha-*DAR	Normal (*p*-value)	0.000	0.928	0.000	0.000	0.000	0.900	0.651	0.000
		Power law (*p*-value)	0.297	0.584	0.999	0.959	0.925	0.388	0.071	0.993
		Power law (*K*)	6.261	9.685	5.868	6.285	1.860	9.616	5.040	4.037
	*Beta-*DAR	Normal (*p*-value)	0.001	0.000	0.000	0.000	0.000	0.000	0.000	0.000
		Power law (*p*-value)	0.774	0.944	0.348	0.983	NA	0.457	0.966	NA
		Power law (*K*)	8.687	17.611	9.192	9.093	NA	3.005	5.075	NA
*q = 3*	*Alpha-*DAR	Normal (*p*-value)	0.000	0.548	0.000	0.000	0.000	0.528	0.000	0.000
		Power law (*p*-value)	0.890	0.853	0.964	0.997	0.983	0.656	0.137	0.986
		Power law (*K*)	8.367	10.098	5.042	7.049	2.759	10.333	3.435	3.415
	*Beta-*DAR	Normal (*p*-value)	0.001	0.000	0.000	0.000	0.000	0.000	0.000	0.000
		Power law (*p*-value)	0.829	0.852	0.519	0.945	NA	0.996	0.709	NA
		Power law (*K*)	7.641	14.572	10.466	8.536	NA	4.838	3.604	NA

The scaling parameter *z* of intra-DAR profile is significantly larger than the *z* of the inter-DAR profile, i.e., comparing the series of intra-DAR z-*q* with inter-DAR *z*-*q* for each *q* respectively. The intra-PDO profile is significantly smaller that the inter-PDO profile, which can be explained by the relationship between *z* and *g* (Equation 7). Since PDO profile is a measure of similarity (PDO: pair-wise diversity overlap), the smaller PDO indicates larger similarity. Equation (7) shows that DAR-profile *z* is inversely related to PDO-profile *g*. That is, higher *z* or lower *g* means higher dissimilarity (difference) or lower similarity (overlap) and *vice versa*.

The intra-*z* is approximately 170–470% larger than the inter-*z* in the case of alpha-DAR. The difference is slightly smaller in the case of beta-DAR than in alpha-DAR (165–270%). The big difference between the intra-DAR vs. inter-DAR differences in both alpha- and beta-diversity scaling should be anticipated if we recognize that, for example, skin and gut are two very different microbiome habitats and they should exert very different selective forces shaping the microbiome in their respective environments. In contrast, the inter-individual difference of one specific site in terms of the microbiome habitat should obviously be lesser profound. With an analogy, in the case of intra-DAR scaling, we are possibly comparing lake and forest (gut vs. skin of the same individual), and in the case of inter-DAR scaling, we are comparing two lakes (gut of one individual vs. gut of another individual). Obviously, the huge difference between the intra-DAR scaling parameter and inter-DAR scaling parameter is because the intra-DAR is about the scaling of biodiversity across different microbiome habitats or human organs (tissues) of one individual, while the inter-DAR is about the scaling of biodiversity across the same type of microbiome habitat of a population of individuals. The finding therefore suggests that the diversity difference (heterogeneity) among microbiome habitats is larger than the inter-individual difference of the same habitat type.

The intra-MAD (*D*_max_) is smaller than the inter-MAD (*D*_max_), but the magnitude of difference is slightly smaller than the differences in the DAR and PDO profiles. Nevertheless, the difference revealed by MAD is certainly interesting. For example, the difference in MAD at *q* = 0 (i.e., OTU richness) is (intra = 6,068 vs. inter = 11,990. This suggests that, on average, the maximal number of OTUs (at the species equivalent level or 97% level of similarity) hosted by an individual is approximately ½ of that hosted by a human population. We caution to extend this number to the humankind, since although the sample size of the HMP datasets we adopted to establish the MAD profiles and make the prediction, is to date the largest, the number may change in future when larger human microbiome datasets are collected.

The HMP, MetaHIT, and other follow-up similar projects open a new era in biomedical research. In fact, apart from the growing list of the so-termed microbiome-associated diseases including obesity, diabetes, IBD (inflammatory bowel disease), bacterial vaginosis, rectal cancer, HIV, gout, infertility, mastitis, and periodontitis, the call for the *conservation* of human microbiome for the benefits of our health has begun to receive increasing attention in recent years (O'Doherty et al., 2014), which could have been perceived as heresy not long ago in clinical medicine, where bacteria were either treated as human enemies (pathogens) or simply ignored (non-pathogens). In clinical applications, fecal microbiotica transplantation (FMT) or stool transplantation, designed to restore proper gut microbiome biodiversity, is a treatment for diarrhea caused by the Clostridium difficile bacteria infection (CDI), and is now recommended as the most effective therapy for relapsing CDI. Other ongoing investigations related to GI dysbiosis include IBD, irritable bowel syndrome, obesity, diabetes mellitus and even Parkinson's disease (Borody et al., 2013). In those medical interventions requiring the personalized assessment and prediction of the microbial diversities as well as their biogeography, we suggest that the intra-DAR model may find important applications. This will require comparative analysis between the healthy and diseased microbiome samples, for example, by comparing the DAR parameters between the healthy and diseased treatments. However, at this stage, few appropriate datasets exist in the literature to demonstrate the application of intra-DAR in personalized medicine, although there are indeed suitable datasets for comparing inter-DAR parameters (i.e., the DAR models built with cross-individual microbiome samples) between the healthy and diseased cohorts and we are working their potential diagnostic applications. Still, we expect that the intra-DAR should be more useful given its individualized nature given that the intra-DAR model is built from multi-site samples taken from a single individual (i.e., each individual has his or her own intra-DAR model parameters).

The modern life style and industrialized food production have exerted significant impacts on the diversity of our gut microbiome, which has been confirmed by several high-profile studies during the last few years (e.g., Yatsunenko et al., 2012; Ordiz et al., 2015; Bahrndorff et al., 2016). It is expected that conserving the biodiversity of human microbiome should be put on the agenda of public health, much similar to public awareness of the need to conserve the biodiversity on the earth planet. Indeed, the classic SAR (species-area relationship) has been playing a critical role in the conservation of plants and animals and is a fundamental theory in conservation biology. We believe that DAR, as a general extension to the SAR, should have a similar role to play in the conservation of microbiome diversity.

Obviously, the potential applications of intra-DAR are not limited to the human microbiome. For example, imagine that we wish to investigate the altitudinal scaling of biodiversity of Mount Everest. By taking diversity samples at different altitudes, an intra-DAR model may be built for the mountain. Similarly, one may build intra-DAR models for biodiversity scaling of ocean microbiome by taking samples from different sea depth.

Finally, we would like to discuss some possible issues and remedies in DAR modeling and applications, which were rightly pointed out by two anonymous expert reviewers. Here we devote the remainder of this section to discuss them.

- The first issue is the current lack of appropriate data to demonstrate the potential applications of intra-DAR, as already discussed in the previous paragraphs.
- Second, some of the recent post-OTU clustering approaches such as DADA2 (Callahan et al., 2016), DEBLUR (Amir et al., 2017), should improve the quality of OTU binning and abundance estimations, and ultimately make the estimates of Hill numbers and DAR parameters more reliable.
- Third, the issue of possible uneven sequencing depth among samples may influence the estimates of DAR parameters, although this was not an issue with the HMP datasets we utilized in this article. When the sequencing depth is uneven, theoretically, there may not be a perfect solution. Nevertheless, three measures should be helpful for remedying the problem. One remedy measure is to use the rarefaction approach, which has already been developed by Chao et al. (2016) for the estimation of Hill numbers. A second approach is to adopt the random permutations of samples before accumulating the samples for building DAR models. In our study, we generated 100 times of random permutations of the samples from 15 to 18 body sites of an individual subject, and built one intra-DAR model for each of the 100 permutations. The average parameters from the 100 intra-DAR models were adopted as the final DAR model parameters for an individual. In this study, the measure was taken for illuminating the influence of arbitrarily ordering the accumulation sequences, but it should also be helpful for alleviating the effect of uneven sequencing depth among samples. A third possible approach could be to control the numbers of reads for all samples being approximately equal, e.g., all samples are normalized to 5,000 reads by various schemes such as random sampling.
- Since the characterization of the microbial communities is complex and often depends on DNA extraction, marker gene, sequencing platform and bioinformatics pipelines, the MAD values are not absolute truth. In other words, MAD could be variable in practice. Ideally, when DNA sequencing technologies and bioinformatics pipelines are standardized, the DAR parameters should be rather stable. Accordingly, MAD should be microbiome-specific to a large extent. In the case of intra-DAR (i.e., within-body DAR), MAD should be individual-specific. From its calculation formula, three parameters determine the value of MAD. Parameter *z* should be community (microbiome) specific (invariant theoretically). Parameter *c* should also be rather stable. The DAR parameter with potentially highest variability should be parameter *a*. From the experience with power law modeling (e.g., Ma, 2015), parameter *a* may be influenced by “sampling effects,” referring to the reality that we are sequencing microbiome sampled from microbiome habitats. The sampling effects are primarily determined by sequencing platform including bioinformatics pipelines. Resolving sampling effects may ultimately depend on the standardization of sequencing platforms.

Host environment, particularly, the health status of host or the impact of human microbiome-associated diseases, may influence DAR parameters, including MAD. Indeed, MAD may be strongly influenced by health status or occurrence of disease. However, as explained previously, at this stage, we do not have datasets available that can test the hypothesis because collecting the datasets for intra-DAR modeling (which requires sampling both the healthy and diseased individuals at multiple sites simultaneously), though not necessarily very difficult, is not a common practice yet in human microbiome research. We hope this study will motivate investigators to pursue such data collections in future.

## Data accessibility

The HMP healthy cohort dataset utilized in this study is available at: http://hmpdacc.org.

## Author contributions

ZM originally formulated the idea and developed methodology. LL performed the computation. WL collaborated in computing and testing. ZM wrote and revised the manuscript. All authors approved the submission.

### Conflict of interest statement

The authors declare that the research was conducted in the absence of any commercial or financial relationships that could be construed as a potential conflict of interest.
