# Supplementary material for: Assessing and Interpreting the Within-Body Biogeography of Human Microbiome Diversity
Source: Front Microbiol. 2018 Aug 7;9:1619. doi: 10.3389/fmicb.2018.01619 (PMC6090070; doi:10.3389/fmicb.2018.01619)
Supplement: Supplementary file 1 [file Data_Sheet_1.PDF]

Supplementary Information for Ma *et al.* (2018) Assessing and interpreting the within-body biogeography of human microbiome diversity

List of Supplementary Documents

Supplementary Information for "Materials and Methods" section
Supplementary information for the computational procedures for DAR analysis

Supplementary Information for "Results and Discussion" section
Table S1. The intra-individual (within-body) *alpha*-DAR modeling for the HMP dataset (Full version, Demo version is in Table 1)
Table S2. The intra-individual (within-body) *beta*-DAR modeling for the HMP dataset (Full version, Demo version is in Table 1)
Table S3. The numbers of sequencing reads and species (OTUs) from individual samples of the HMP cohort (see separate Excel file)

Supplementary information for the Computational Procedures for DAR analysis

To save page space, the methods description in the main document was kept brief, and some detailed description on the computational models and procedures for DAR analysis are provided as follows.

Definitions of alpha and beta diversities

We use the Hill numbers to measure both alpha and beta diversities, and we adopt *multiplicative version* of the Hill numbers as beta diversities.

The Hill numbers, originally introduced as an *evenness* index from economics by Hill (1973) who was apparently inspired by Renyi's (1961) general entropy of order, has not received the attention it deserves until recent years. Chao et al. (2012) further clarified Hill's numbers for measuring alpha diversity as (with slightly different symbols and notations):

$$^{q}D = \left(\sum_{i=1}^{S} p_i^q \right)^{1/(1-q)} \quad (1)$$

where S is the number of species, q is the order number.

The Hill number is undefined for q=1, but its limit as q approaches to *1* exists in the following form:

$$^{1}D = \lim_{q \to 1} {^{q}D} = \exp\left(-\sum_{i=1}^{s} p_i \log(p_i) \right) \quad (2)$$

The parameter q determines the sensitivity of the Hill number to the relative frequencies of species abundances. When q=0, the species abundances do not count at all and $^{0}D=S$, *i.e.,* species richness. When q=1, ^{1}D equal the *exponential* of Shannon entropy, and is interpreted as the number of typical or common species in the community. When q=2, ^{2}D equal the reciprocal of Simpson index, *i.e.*,

$$^{2}D = (1/\sum_{i=1}^{S} p_i^2) \quad (3)$$

which is interpreted as the number of dominant or very abundant species in the community (Chao et al. 2012).

The general interpretation of diversity of order q is that the community contains ${}^{q}D=x$ equally abundant species. Then, the diversity of a community can be measured with a series of Hill numbers, possibly plotted on a single graph as a 'continuous' function of the parameter q. Chao et al (2012) termed the series of plots "*community diversity profile*" that characterizes the species-abundance distribution of a community and offers complete information on its diversity. Since all Hill numbers are in units of species, and in fact, they are referred to as the effective number of species or as species equivalents, therefore, intuitively, Hill numbers should follow the same or similar pattern of SAR.

Recent studies (*e.g.*, Jost 2007, Ellison 2010, Chao et al. 2012, Gotelli & Chao 2013) have advocated the use of multiplicatively defined beta-diversity, rather than additively defined, by partitioning gamma diversity into the product of alpha and beta, in which both alpha and gamma diversities are measured with the Hill numbers. For example, Jost (2007) demonstrated that the partition of Hill numbers into independent alpha (within community) and beta (between communities) is necessarily multiplicative such as:

$$ {}^{q}D_{\beta} = {}^{q}D_{\gamma} / {}^{q}D_{\alpha} \qquad (4) $$

This beta diversity derived from the above partition takes the value of 1 if all communities are identical, the value of N (the number of communities) when all the communities are completely different from each other (there are no shared species). With Whittaker (1972) words, this beta diversity measures "*the extent of differentiation of communities*;" or with Jost (2007) words, "*the effective number of completely distinct communities*." Indeed, recent advances (Jost 2007, Ellison 2010, Chao 2012, Chao et al. 2014, Chiu et al. 2014, Chao & Jost 2015) have made a convincing case that Hill numbers and multiplicative beta-partition offer to date the most generally consistent and appropriate, yet simple solution to investigate diversity. For this we stick to the Hill numbers and multiplicative beta-diversity in this study.

To compute the Hill numbers of beta diversity with Eq. (4), we first must compute the Hill numbers of gamma diversity of multiple local communities (regional or meta-community). Let us consider a fixed set of N local communities. Assume that there are S species in the pooled assemblages (meta-community), y_{ij} is the abundance of the i-th species in the j-th local community. Let $y_{++} = \sum_{i=1}^{S}\sum_{j=1}^{N} y_{ij}$ be the total abundance in the meta-community, and let $y_{+j} = \sum_{i=1}^{S} y_{ij}$ be the community size of the j-th local community. Then we may denote the value y_{ij} as $y_{ij} = y_{++}(y_{+j}/y_{++})(y_{ij}/y_{+j}) = y_{++}w_j p_{ij}$, where $p_{ij} = y_{ij}/y_{+j}$ is the relative abundance of the i-th species in the j-th community, and $w_j = y_{+j}/y_{++}$ is the relative community size or the weigh of the j-th community, with $\sum_{j=1}^{N} w_j = 1$. For the gamma community, we pool the species abundances across local communities, and let $y_{i+} = \sum_{j=1}^{N} y_{ij} = y_{++}\sum_{j=1}^{N} w_j p_{ij}$ be the total value of the i-th species in the meta-community. Then the alpha diversity and gamma diversity of the meta-community can be computed as (5) & (6) respectively,

$$ {}^{q}D_{\alpha} = \frac{1}{N}\left\{\sum_{i=1}^{S}\sum_{j=1}^{N}\left(\frac{y_{ij}}{y_{++}}\right)^{q}\right\}^{1/(1-q)} = \frac{1}{N}\left\{\sum_{i=1}^{S}\sum_{j=1}^{N}\left(w_j p_{ij}\right)^{q}\right\}^{1/(1-q)} \quad (q \neq 1) \qquad (5) $$

$$ {}^{q}D_{\gamma} = \left\{\sum_{i=1}^{S}\left(\frac{y_{i+}}{y_{++}}\right)^{q}\right\}^{1/(1-q)} = \left\{\sum_{i=1}^{S}\left(\sum_{j=1}^{N} w_j p_{ij}\right)^{q}\right\}^{1/(1-q)} \quad (q \neq 1). \qquad (6) $$

When q=1, the corresponding ${}^1D_\alpha = \lim_{q \to 1} {}^qD_\alpha$ and ${}^1D_\gamma = \lim_{q \to 1} {}^qD_\gamma$, and their detailed formulae can be found in Chiu et al. (2014) and we omit them here.

In this article, we compute diversities until q=3, *i.e.,* to the third order, which includes traditional *species richness* (q=0), the exponential of Shannon index (q=1), the reciprocal of Simpson index (q=2) and one additional set of indexes for q=3.

Fitting the DAR models and quantifying the DAR profiles

The signs of DAR parameters: Occasionally, there may be the cases when the DAR model parameters do not make sense ecologically. Here we use PLEC model as example to explain the principles we adopt for dealing with the exceptional cases. With PLEC model [eqns. (4) & (6)], the sign of parameter z and d must be opposite to have $A_{\max}>0$ [eqn. (8)], otherwise, $A_{\max}<0$ the model does not have an extreme value. In this exceptional case, we simply discarded a permutation that generated the exception, and added another round of model fitting by randomly taking another order of samples from the total permutation. The additional fittings may be repeated as needed until a positive exponent is obtained. Note that we only remove exceptions that do not make sense ecologically such as $A_{\max}<0$ (negative number of accrual is not possible). For example, negative z and positive d in PLEC model are possible for DAR beyond species richness (q=0), we hence preserve DAR models with such parameter combination. But there is a complication from allowing such cases, that is, the function (PLEC model) does not have a maximum when $z<0$ and $d>0$. We adopt the following two principles to deal with this issue: (*i*) Using the average parameters from 100 times of re-sampling modeling of PLEC models, or (*ii*) if (*i*) fails, using individual models from the re-sampling that satisfy the maximum condition ($z>0$, $d<0$) only. Both options may be imperfect, but are rather reasonable approximations.

Note that occasional failures or seemingly abnormal parameters *out of 100 times* of re-sampling may not mean the model failure at all, because as long as the number of successful fittings reach a big sample (>50 times), we can still reliably estimate the model parameters by take the average parameters of the good fittings.

The scheme to accrue diversity: After dealing with the issues associated with arbitrarily ordering the areas (microbiome sites in our case) to accrue, we still need to design a proper scheme to accrue diversity. Although the accumulation of species in traditional SAR is well defined and there is no ambiguity on how the species counts are computed once the scheme for area accrual is decided, the computation of the diversity accrual is still largely an uncharted area, and there may be more than one scheme to accumulate diversity across space, especially for the accrual of beta-diversity. To devise what we believe to be the most appropriate and also natural methods to accrue diversity, we follow the following three principles. The first is to use the Hill numbers, or what Jost (2007) termed the *true* diversity; the second is to follow the essence of SAR, as captured by the word "accumulation" or "aggregate," *i.e.*, species (diversity) are accumulated for the accrued areas; the third is that the diversity scaling model should be useful for *predicting* diversity at different levels of areas accumulated. We consider these three principles as axioms in traditional SAR and we believe that any extension from SAR to DAR should not violate them.

The last principle is a major reason why SAR has been a central theme of both community ecology and conservation biology, and is obviously important for understanding the biogeography of human microbiome. One important advantage for us to stick to the three principles, which are embodied in the traditional SAR theory, is that our new DAR should inherit

many of the insights and applications that traditional SAR has reveled and offered. Based on these three principles, we construct the following procedures to accumulate diversity over areas.

To accrue alpha diversity across areas, at each accrual step, we *first* simply add up all the OTU lists (rows) in the OTU table of the communities up to the accrual step. Assuming there are N communities, at accrual step i=1, 2,…, N-1, we simply add up the rows in the standard OTU table corresponding to the first i communities, until the last row is added when the accrual is completed. For each of the aggregate (accumulated) community, we compute its alpha-diversity with Eq. (1) or Eq. (2) (for q=1) with the added-up OTU lists for the accrual step. The resulted pairs of accrued alpha diversities and areas are regressed to fit the DAR model.

An alternative accrual scheme is to use Eq. (5), but the output with Eq. (5) would be a measure of average single community (local community) diversity, which is not a cumulative quantity and is hence not a measure that we are interested in to investigate DAR relationship. This is because Eqn. (5) is constrained by its mission to partition gamma diversity [Eq. (6)] multiplicatively into alpha diversity [Eq. (5)] and beta-diversity [Eq. (4)]. Because it is a measure of the *average* alpha diversity of the local communities that constitute the regional meta-community, rather than the alpha-diversity of aggregate (pooled or accumulated) communities, therefore, this alternative scheme contradicts the second and third principles we identified above.

Yet another alternative accrual scheme is to use Eq. (6), which calculates the gamma diversity of pooled (aggregate or accumulated) regional or meta-community. Although conceptually it computes the gamma diversity of the aggregate community, it is actually equal to the alpha diversity we calculated for the aggregate community with Eqns. (1) or (2) (when q=1) when the community weight (w_j) is equal for all N communities (*i.e.*, w_j=1/N). Since the equal weight community assumption is largely true even if the weights are not exactly equal, we can expect that the difference between the results from Eqns. (1) and (6) should not to have significant influence on DAR modeling results. Therefore, we do not see an advantage with this alternative accrual scheme, especially the potential confusion in terminology. In this case, whether it is called alpha or gamma diversity matters little, and from the perspective of SAR (DAR), we prefer to use the term alpha-diversity.

To the best of our knowledge, scaling of beta-diversity has not been approached in the existing literature. Therefore, we have a benefit to prioritize the potential utilization that the beta-diversity scaling relationship may possess. We believe that the three axioms (principles) we identified above should also guide the extension of SAR to beta-DAR. Furthermore, the capability to predict beta-diversity of aggregate community, as designated in principle 3, is also the priority that the beta-DAR should pursue, in our belief. Through trial-and-error exploration, we found that the following scheme for accruing beta-diversity over areas best satisfy the three principles we set for developing beta-DAR.

Formally, to accrue beta-diversity across areas, we start the computational procedure with two local communities (samples) by using the formulae specified by Eq. (4)-(6), from which the first beta-diversity value is computed for the two initial communities. For each newly added community (sample) at the accrual step i (*i=3, 4, ... N*), we simply run the same computation procedure (same equations) with *3*, *4*, … N samples, until all N communities are accrued for their beta-diversity. With each newly added local community, we obtain a new Hill numbers of beta-diversity, until all N communities are accrued for their beta-diversity. The series of beta-diversities are regressed with their respective areas accrued at each step. Obviously, this accrual scheme calculates the *accumulated beta-diversity* of N communities (of individuals in the case of

human microbiome). It is also the *maximal* difference among *N* communities in terms of beta-diversity.

References for the Supplementary Document

Chao A, CH Chiu, TC Hsieh (2012) Proposing a resolution to debates on diversity partitioning. *Ecology*, 93(9): 2037-2051.

Chao A, CH Chiu, & L Jost (2014) Unifying species diversity, phylogenetic diversity, functional diversity and related similarity and differentiation measures through Hill numbers. *Annual Reviews of Ecology, Evolution, and Systematics*, 45:297–324.

Chao, A and L Jost (2015) Estimating diversity and entropy profiles via discovery rates of new species. *Methods in Ecology and Evolution* 2015, doi: 10.1111/2041-210X.12349

Chiu CH, L Jost, A Chao (2014) Phylogenetic beta diversity, similarity, and differentiation measures based on Hill numbers. *Ecological Monographs*, 84(1): 21-44.

Ellison AM (2010) Partitioning diversity. *Ecology* 91:1962– 1963.

Gotelli, N.J. & Chao, A. (2013) Measuring and estimating species richness, species diversity, and biotic similarity from sampling data. Encyclopedia of Biodiversity, vol 5, 2nd ed. (ed. S.A. Levin), pp. 195–211. Academic Press, Massachusetts, USA.

Hill MO (1973) Diversity and evenness: a unifying notation and its consequences. *Ecology,* 54:427-342.

Jost L (2007) Partitioning diversity into independent alpha and beta components. *Ecology* 88:2427–2439.

Renyi A (1961) On measures of entropy and information, p. 547-561. Neyman, ed. 4th *Berkeley symposium on mathematical statistics and probability*. Berkeley.

Table S1. The intra-individual (within-body) *alpha*-DAR modeling for the HMP dataset (Full version, Demo version is in Table 1)

Order	***Subject Number***	**Power Law (PL)**						**PL with Exponential Cutoff (PLEC)**							
		z	**ln(*c*)**	***R***	***p*-value**	***g***	***N****	*z*	***d***	**ln(*c*)**	***R***	***p*-value**	***N****	$A_{\max}$	$D_{\max}$
q=0	132902142	1.061	6.170	0.947	0.000	-0.162	100	1.896	-0.213	6.077	0.979	0.000	100	9	4146.7
	147406386	0.776	6.723	0.988	0.000	0.282	100	1.020	-0.045	6.632	0.996	0.000	100	23	6552.0
	158013734	0.827	6.359	0.977	0.000	0.213	100	1.167	-0.057	6.205	0.990	0.000	100	20	5200.5
	158114885	0.776	6.641	0.974	0.000	0.271	100	1.120	-0.055	6.471	0.989	0.000	100	20	6124.7
	158155345	0.730	6.742	0.992	0.000	0.340	100	0.909	-0.033	6.676	0.997	0.000	100	27	6474.3
	158216035	0.744	6.901	0.988	0.000	0.323	100	0.973	-0.045	6.826	0.995	0.000	100	22	6912.1
	158236265	0.802	6.617	0.978	0.000	0.246	100	1.126	-0.057	6.483	0.991	0.000	100	20	6059.9
	158276726	0.851	6.360	0.970	0.000	0.185	100	1.223	-0.060	6.176	0.986	0.000	100	20	5674.4
	158398106	0.790	6.513	0.984	0.000	0.263	100	1.029	-0.044	6.424	0.992	0.000	100	23	5586.2
	158418336	0.811	6.775	0.972	0.000	0.237	100	1.163	-0.069	6.660	0.987	0.000	100	17	6491.1
	158438567	0.857	6.456	0.970	0.000	0.155	100	1.281	-0.079	6.299	0.987	0.000	100	16	5375.7
	158458797	0.810	6.214	0.985	0.000	0.240	100	1.082	-0.046	6.090	0.994	0.000	100	24	4584.4
	158479027	0.918	6.544	0.973	0.000	0.062	100	1.381	-0.091	6.393	0.989	0.000	100	15	6414.6
	158499257	1.018	6.249	0.968	0.000	-0.077	100	1.635	-0.137	6.110	0.988	0.000	100	12	5083.8
	158721788	0.831	6.729	0.987	0.000	0.211	100	1.110	-0.058	6.652	0.995	0.000	100	19	6747.5
	158742018	0.815	6.448	0.983	0.000	0.231	100	1.117	-0.048	6.299	0.994	0.000	100	23	5922.2
	158802708	0.845	6.957	0.974	0.000	0.175	100	1.233	-0.072	6.812	0.990	0.000	100	17	8753.8
	158822939	0.707	7.599	0.984	0.000	0.362	100	0.939	-0.046	7.523	0.993	0.000	100	21	12395.8
	158883629	0.806	6.557	0.976	0.000	0.237	100	1.146	-0.057	6.402	0.989	0.000	100	20	5931.7
	158924089	0.944	7.057	0.951	0.000	0.008	100	1.609	-0.124	6.811	0.980	0.000	100	13	11248.6
	158944319	0.831	6.757	0.983	0.000	0.209	100	1.111	-0.050	6.641	0.993	0.000	100	22	7999.9
	158964549	0.904	6.479	0.967	0.000	0.112	100	1.338	-0.081	6.318	0.985	0.000	100	17	6222.7
	158984779	0.892	6.436	0.978	0.000	0.129	100	1.262	-0.077	6.333	0.990	0.000	100	16	5433.1
	159005010	0.909	6.712	0.960	0.000	0.045	100	1.439	-0.085	6.450	0.982	0.000	100	17	8768.9
	159025240	1.062	5.909	0.939	0.000	-0.314	100	1.943	-0.142	5.473	0.973	0.000	100	14	5530.2
	159085930	0.850	6.915	0.973	0.000	0.174	100	1.228	-0.061	6.728	0.988	0.000	100	20	9796.7
	159146620	0.764	6.779	0.986	0.000	0.298	100	1.033	-0.050	6.679	0.995	0.000	100	21	6454.4
	159166850	0.729	7.076	0.973	0.000	0.334	100	1.036	-0.057	6.962	0.988	0.000	100	18	7542.8
	159227541	0.803	6.813	0.975	0.000	0.242	100	1.161	-0.058	6.636	0.990	0.000	100	20	7812.6
	159247771	0.995	6.240	0.951	0.000	-0.111	100	1.648	-0.105	5.917	0.977	0.000	100	16	6678.3
	159389382	0.904	6.603	0.977	0.000	0.113	100	1.258	-0.066	6.472	0.990	0.000	100	19	7510.4
	159470302	0.851	7.088	0.957	0.000	0.166	100	1.323	-0.076	6.854	0.980	0.000	100	17	11081.5
	159713063	0.755	7.267	0.977	0.000	0.297	100	1.082	-0.052	7.106	0.991	0.000	100	21	10903.5
	159733294	0.820	7.083	0.971	0.000	0.217	100	1.201	-0.061	6.894	0.987	0.000	100	20	10567.5
	160744799	1.149	5.197	0.944	0.000	-0.483	100	2.057	-0.161	4.821	0.975	0.000	100	13	3010.7
	160825720	0.874	6.358	0.983	0.000	0.161	100	1.197	-0.063	6.253	0.993	0.000	100	19	5283.8
	160866180	0.902	6.002	0.986	0.000	0.114	100	1.184	-0.047	5.874	0.995	0.000	100	25	4911.3
	160886410	0.902	6.186	0.974	0.000	0.098	100	1.319	-0.082	6.051	0.988	0.000	100	16	4431.0
	160906640	0.819	6.557	0.991	0.000	0.233	100	1.041	-0.046	6.495	0.996	0.000	100	22	5974.7
	160947100	0.975	6.097	0.972	0.000	-0.122	100	1.528	-0.103	5.891	0.988	0.000	100	15	4840.4
	160967330	0.813	6.484	0.987	0.000	0.240	100	1.077	-0.052	6.398	0.995	0.000	100	21	5369.9
	160987560	0.910	6.316	0.975	0.000	0.062	100	1.348	-0.086	6.173	0.990	0.000	100	16	5092.1
	161007791	0.826	6.287	0.987	0.000	0.220	100	1.069	-0.045	6.197	0.994	0.000	100	24	4964.4
	161270782	1.037	5.970	0.973	0.000	-0.257	100	1.622	-0.115	5.779	0.988	0.000	100	14	4675.3
	161311242	0.802	6.481	0.994	0.000	0.255	100	0.995	-0.043	6.438	0.998	0.000	100	23	5289.8
	161412393	0.793	6.665	0.989	0.000	0.263	100	1.041	-0.074	6.671	0.995	0.000	100	14	4346.5
	161473083	0.920	6.040	0.981	0.000	0.081	100	1.239	-0.051	5.882	0.991	0.000	100	24	5361.5
	161554003	0.765	6.474	0.993	0.000	0.299	100	0.947	-0.038	6.423	0.998	0.000	100	25	5036.5
	178713055	1.469	5.010	0.914	0.000	-1.257	100	2.978	-0.297	4.519	0.964	0.000	100	10	4496.5
	184349034	0.915	6.666	0.981	0.000	0.100	100	1.335	-0.126	6.675	0.992	0.000	100	11	4874.2
	208027353	0.815	6.594	0.979	0.000	0.228	100	1.141	-0.055	6.445	0.991	0.000	100	21	6404.7

	246515023	0.890	6.028	0.981	0.000	0.140	100	1.168	-0.045	5.890	0.990	0.000	100	26	5088.5
	256789458	1.375	5.467	0.945	0.000	-1.202	100	2.694	-0.336	5.321	0.978	0.000	100	8	3781.8
	275382046	1.018	6.149	0.974	0.000	-0.062	100	1.571	-0.182	6.204	0.989	0.001	100	9	3035.0
	289996019	0.788	6.679	0.986	0.000	0.263	100	1.064	-0.061	6.617	0.994	0.000	100	17	5389.9
	295137534	1.133	5.539	0.964	0.000	-0.367	100	1.884	-0.157	5.331	0.985	0.000	100	12	3414.0
	336497421	0.844	6.586	0.972	0.000	0.192	100	1.235	-0.081	6.478	0.987	0.000	100	15	5434.8
	368533040	0.853	6.183	0.988	0.000	0.188	100	1.091	-0.044	6.095	0.994	0.000	100	25	4908.9
	370027359	0.809	6.628	0.987	0.000	0.242	100	1.061	-0.047	6.535	0.995	0.000	100	23	6533.7
	370425937	0.853	6.242	0.978	0.000	0.180	100	1.204	-0.057	6.068	0.991	0.000	100	21	5151.5
	375450439	0.854	6.542	0.977	0.000	0.170	100	1.195	-0.055	6.373	0.990	0.000	100	22	7040.6
	404239096	0.784	6.756	0.994	0.000	0.276	100	0.954	-0.035	6.709	0.997	0.000	100	27	7330.6
	414519462	0.872	6.429	0.964	0.000	0.148	100	1.282	-0.066	6.226	0.982	0.000	100	19	6307.5
	451588811	1.129	5.976	0.948	0.000	-0.365	100	1.957	-0.163	5.707	0.976	0.000	100	12	5521.6
	465578759	0.900	6.261	0.973	0.000	0.110	100	1.268	-0.059	6.079	0.986	0.000	100	21	5981.9
	492786515	0.939	6.098	0.970	0.000	-0.068	100	1.487	-0.102	5.895	0.987	0.000	100	15	4410.3
	508703490	1.136	5.537	0.936	0.000	-0.396	100	2.134	-0.186	5.166	0.972	0.000	100	11	3795.9
	516889361	0.947	6.597	0.961	0.001	0.023	100	1.330	-0.126	6.636	0.986	0.001	100	11	4615.9
	517810313	0.737	6.758	0.991	0.000	0.329	100	0.905	-0.031	6.696	0.996	0.000	100	29	6890.6
	550534656	1.071	6.064	0.980	0.004	-0.160	100	1.815	-0.307	6.273	0.996	0.009	100	6	2170.4
	553359145	0.801	6.566	0.977	0.000	0.240	100	1.137	-0.057	6.413	0.991	0.000	100	20	5928.1
	604812005	0.869	6.156	0.988	0.000	0.169	100	1.128	-0.051	6.072	0.995	0.000	100	22	4628.9
	612472597	0.869	6.652	0.969	0.000	0.156	100	1.259	-0.063	6.460	0.986	0.000	100	20	7931.3
	638754422	0.831	6.463	0.991	0.000	0.218	100	1.046	-0.038	6.374	0.997	0.000	100	28	6607.6
	643185023	1.268	5.436	0.947	0.000	-0.743	100	2.238	-0.215	5.217	0.978	0.000	100	10	3721.9
	650853796	0.884	6.385	0.983	0.000	0.139	100	1.202	-0.063	6.282	0.993	0.000	100	19	5619.3
	658594300	1.423	5.174	0.926	0.002	-1.323	100	2.731	-0.392	5.203	0.974	0.001	100	7	2380.4
	663835652	0.857	6.526	0.979	0.000	0.173	100	1.219	-0.071	6.408	0.991	0.000	100	17	5716.1
	668248235	0.754	6.888	0.991	0.000	0.311	100	0.949	-0.036	6.815	0.996	0.000	100	26	7797.0
	682102541	0.931	6.451	0.955	0.013	0.026	100	1.530	-0.238	6.594	0.988	0.024	86	6	2716.5
	682449369	0.835	6.466	0.968	0.000	0.183	100	1.255	-0.071	6.274	0.986	0.000	100	18	5593.5
	686765762	0.834	6.537	0.986	0.000	0.210	100	1.091	-0.048	6.442	0.993	0.000	100	23	6401.7
	706846339	0.905	6.772	0.947	0.016	0.040	100	1.434	-0.214	6.914	0.990	0.020	93	7	3679.5
	737052003	0.823	6.680	0.979	0.000	0.215	100	1.164	-0.060	6.539	0.992	0.000	100	19	6783.2
	739574095	0.851	6.143	0.986	0.000	0.183	100	1.173	-0.118	6.202	0.994	0.002	100	10	2260.5
	763395383	0.741	6.932	0.991	0.000	0.326	100	0.932	-0.037	6.870	0.996	0.000	100	25	7562.4
	763435843	0.814	6.858	0.977	0.000	0.145	100	1.232	-0.082	6.722	0.990	0.000	100	15	6808.4
	763456073	0.806	6.498	0.976	0.000	0.233	100	1.151	-0.061	6.355	0.990	0.000	100	19	5357.1
	763476303	0.798	6.482	0.993	0.000	0.258	100	0.968	-0.033	6.427	0.997	0.000	100	29	6095.1
	763516763	1.010	6.095	0.965	0.000	-0.169	100	1.617	-0.113	5.870	0.984	0.000	100	14	5205.6
	763536994	0.839	6.935	0.971	0.000	0.189	100	1.234	-0.078	6.807	0.987	0.000	100	16	7988.2
	763557224	0.992	5.787	0.961	0.000	-0.080	100	1.588	-0.117	5.593	0.982	0.000	100	14	3441.4
	763597684	0.723	7.451	0.989	0.000	0.348	100	0.939	-0.042	7.381	0.996	0.000	100	22	11500.2
	763638144	0.973	6.170	0.956	0.000	0.003	100	1.531	-0.094	5.916	0.980	0.000	100	16	5746.5
	763698834	0.768	7.094	0.980	0.000	0.286	100	1.071	-0.051	6.956	0.993	0.000	100	21	9375.1
	763719065	0.885	6.067	0.969	0.000	0.137	100	1.271	-0.062	5.876	0.986	0.000	100	20	4641.0
	763759525	0.872	6.559	0.977	0.000	0.160	100	1.198	-0.053	6.398	0.990	0.000	100	23	7688.7
	763820215	0.870	6.217	0.974	0.000	0.160	100	1.250	-0.067	6.059	0.988	0.000	100	19	4735.8
	763840445	0.872	6.538	0.968	0.000	0.139	100	1.303	-0.069	6.325	0.986	0.000	100	19	6939.8
	763860675	0.832	6.627	0.982	0.000	0.213	100	1.122	-0.057	6.533	0.991	0.000	100	20	6329.1
	763880905	0.754	6.840	0.989	0.000	0.308	100	0.986	-0.048	6.776	0.996	0.000	100	20	6404.1
	763901136	0.987	6.009	0.965	0.000	-0.053	100	1.537	-0.089	5.737	0.986	0.000	100	17	5364.4
	763921366	0.840	6.549	0.973	0.000	0.191	100	1.206	-0.059	6.368	0.988	0.000	100	21	6673.6
	763982056	0.770	6.836	0.989	0.000	0.290	100	0.993	-0.041	6.754	0.995	0.000	100	24	7440.9
	764002286	0.803	6.878	0.983	0.000	0.249	100	1.077	-0.051	6.777	0.993	0.000	100	21	7972.1
	764042746	0.834	6.268	0.987	0.000	0.209	100	1.084	-0.044	6.164	0.994	0.000	100	25	5166.2

	764062976	0.923	6.316	0.960	0.000	0.072	100	1.420	-0.084	6.089	0.981	0.000	100	17	5948.0
	764083206	0.757	6.997	0.987	0.000	0.306	100	0.988	-0.043	6.911	0.994	0.000	100	23	8257.8
	764143897	0.822	6.672	0.970	0.000	0.217	100	1.217	-0.064	6.476	0.988	0.000	100	19	6990.0
	764184357	0.902	6.281	0.974	0.000	0.114	100	1.306	-0.065	6.082	0.990	0.000	100	20	5976.5
	764224817	0.863	6.656	0.983	0.000	0.166	100	1.174	-0.058	6.540	0.993	0.000	100	20	7313.2
	764245047	0.862	6.174	0.970	0.000	0.169	100	1.245	-0.071	6.032	0.985	0.000	100	17	4228.1
	764285508	0.733	6.737	0.982	0.000	0.334	100	1.051	-0.081	6.702	0.994	0.000	100	13	4211.9
	764305738	0.775	6.833	0.987	0.000	0.284	100	1.033	-0.048	6.737	0.995	0.000	100	22	7147.7
	764325968	0.901	6.712	0.975	0.000	0.073	100	1.324	-0.079	6.555	0.989	0.000	100	17	7852.7
	764346198	0.821	6.417	0.987	0.000	0.229	100	1.092	-0.046	6.294	0.995	0.000	100	24	5811.2
	764366428	0.805	6.225	0.976	0.000	0.237	100	1.148	-0.064	6.098	0.989	0.000	100	18	3886.2
	764447348	1.007	5.926	0.969	0.000	-0.043	100	1.423	-0.082	5.790	0.985	0.000	100	17	4586.1
	764467579	0.900	6.302	0.973	0.000	0.108	100	1.325	-0.075	6.126	0.989	0.000	100	18	5460.3
	764487809	0.810	6.805	0.987	0.000	0.241	100	1.066	-0.048	6.710	0.995	0.000	100	22	7746.3
	764508039	0.851	6.528	0.986	0.000	0.189	100	1.091	-0.047	6.450	0.993	0.000	100	23	6549.2
	764588959	0.903	6.072	0.971	0.000	0.104	100	1.336	-0.077	5.892	0.988	0.000	100	17	4347.0
	764649650	0.822	6.515	0.986	0.000	0.226	100	1.075	-0.041	6.390	0.994	0.000	100	26	6881.6
	764669880	0.795	6.761	0.983	0.000	0.258	100	1.073	-0.052	6.657	0.992	0.000	100	21	6894.5
	764710340	0.941	5.348	0.954	0.000	0.049	100	1.335	-0.118	5.356	0.978	0.001	100	11	1423.1
	764750800	0.765	6.927	0.990	0.000	0.295	100	0.993	-0.045	6.853	0.996	0.000	100	22	7616.8
	764811490	0.830	6.680	0.983	0.000	0.216	100	1.119	-0.049	6.549	0.993	0.000	100	23	7626.0
	764831721	0.823	6.750	0.988	0.000	0.227	100	1.094	-0.056	6.675	0.996	0.000	100	19	6806.7
	764872181	0.740	6.704	0.992	0.000	0.328	100	0.931	-0.037	6.642	0.997	0.000	100	25	6016.8
	764892411	0.771	6.679	0.992	0.000	0.290	100	0.962	-0.035	6.608	0.996	0.000	100	27	6768.3
	764953101	0.811	6.786	0.990	0.000	0.242	100	1.054	-0.054	6.731	0.996	0.000	100	20	6710.0
	765013792	0.821	6.396	0.980	0.000	0.220	100	1.149	-0.055	6.247	0.992	0.000	100	21	5352.1
	765034022	0.854	6.311	0.974	0.000	0.174	100	1.239	-0.065	6.136	0.989	0.000	100	19	5184.2
	765074482	0.834	6.572	0.986	0.000	0.207	100	1.105	-0.051	6.472	0.994	0.000	100	22	6478.0
	765094712	0.778	6.823	0.991	0.000	0.282	100	0.996	-0.043	6.752	0.997	0.000	100	23	7255.5
	765155402	0.797	6.595	0.991	0.000	0.260	100	1.002	-0.038	6.518	0.997	0.000	100	26	6569.6
	765195863	0.795	6.575	0.986	0.000	0.258	100	1.037	-0.039	6.455	0.994	0.000	100	27	6791.1
	765216093	0.782	6.708	0.990	0.000	0.276	100	0.985	-0.038	6.633	0.996	0.000	100	26	7059.7
	765256553	0.841	6.546	0.974	0.000	0.195	100	1.230	-0.076	6.419	0.989	0.000	100	16	5469.5
	765276783	0.775	6.654	0.992	0.000	0.287	100	0.967	-0.036	6.582	0.997	0.000	100	27	6648.7
	765317243	0.799	6.378	0.986	0.000	0.250	100	1.063	-0.052	6.292	0.994	0.000	100	21	4634.3
	765337473	0.869	6.396	0.972	0.000	0.144	100	1.286	-0.067	6.190	0.989	0.000	100	19	6026.9
	765377934	0.761	6.825	0.988	0.000	0.301	100	0.994	-0.059	6.799	0.995	0.000	100	17	5464.8
	765560005	0.749	6.961	0.991	0.000	0.317	100	0.962	-0.042	6.892	0.997	0.000	100	23	7678.8
	809635352	0.858	6.512	0.974	0.000	0.173	100	1.185	-0.053	6.351	0.986	0.000	100	23	7021.7
	863126187	0.803	6.848	0.975	0.000	0.247	100	1.145	-0.071	6.753	0.990	0.000	100	16	6556.2
	892969023	0.822	6.232	0.989	0.000	0.228	100	1.037	-0.038	6.143	0.995	0.000	100	27	5087.6
	937495960	0.925	6.251	0.987	0.000	0.096	100	1.182	-0.057	6.193	0.993	0.000	100	21	5425.2
	953045535	0.903	6.312	0.977	0.000	0.105	100	1.271	-0.065	6.159	0.989	0.000	100	20	5811.0
	970836795	0.955	6.149	0.965	0.000	-0.065	100	1.534	-0.097	5.885	0.985	0.000	100	16	5315.8
	**Mean**	0.860	6.473	0.972	0.000	0.151	100	1.301	-0.093	6.346	0.989	0.000	100	19	6068.2
	**Std. Err.**	0.068	0.128	0.009	0.000	0.106	0	0.201	0.040	0.126	0.004	0.000	0	0	145.8
q=1	132902142	0.930	4.704	0.900	0.001	0.019	100	1.994	-0.271	4.585	0.960	0.001	100	7	715.5
	147406386	0.617	5.058	0.892	0.001	0.440	100	1.121	-0.094	4.871	0.950	0.000	100	12	686.6
	158013734	0.704	4.944	0.910	0.000	0.343	100	1.237	-0.090	4.701	0.956	0.000	100	14	820.7
	158114885	0.633	5.099	0.822	0.002	0.400	96	1.193	-0.090	4.820	0.903	0.001	96	13	820.8
	158155345	0.523	5.267	0.869	0.001	0.539	98	0.854	-0.063	5.159	0.923	0.001	100	14	685.3
	158216035	0.685	5.221	0.936	0.000	0.370	100	1.152	-0.092	5.069	0.967	0.000	100	13	926.9
	158236265	0.675	4.715	0.821	0.003	0.348	87	1.222	-0.104	4.578	0.890	0.001	94	12	580.0
	158276726	0.803	4.506	0.922	0.000	0.205	100	1.413	-0.098	4.205	0.963	0.000	100	14	707.9
	158398106	0.560	5.438	0.955	0.000	0.523	100	0.920	-0.067	5.304	0.984	0.000	100	14	891.9

	158418336	0.633	5.611	0.942	0.000	0.442	100	1.072	-0.086	5.468	0.976	0.000	100	12	1207.2
	158438567	0.660	5.124	0.927	0.000	0.397	100	1.177	-0.096	4.932	0.969	0.000	100	12	815.6
	158458797	0.674	4.816	0.945	0.000	0.391	100	1.086	-0.069	4.628	0.973	0.000	100	16	684.0
	158479027	0.775	5.366	0.951	0.000	0.262	100	1.253	-0.094	5.210	0.976	0.000	100	13	1342.6
	158499257	0.983	4.622	0.942	0.000	-0.063	100	1.736	-0.167	4.452	0.976	0.000	100	10	882.9
	158721788	0.650	5.300	0.958	0.000	0.415	100	1.041	-0.081	5.192	0.981	0.000	100	13	901.9
	158742018	0.588	5.129	0.882	0.001	0.475	99	0.990	-0.065	4.930	0.936	0.000	99	15	766.9
	158802708	0.690	5.270	0.927	0.000	0.361	100	1.189	-0.093	5.084	0.966	0.000	100	13	1018.6
	158822939	0.470	6.049	0.911	0.000	0.609	100	0.791	-0.063	5.944	0.953	0.000	100	13	1276.8
	158883629	0.629	4.475	0.729	0.006	0.421	57	1.141	-0.113	4.581	0.836	0.004	82	10	437.6
	158924089	0.680	5.561	0.877	0.000	0.352	100	1.331	-0.121	5.319	0.933	0.000	100	11	1309.2
	158944319	0.736	4.168	0.778	0.006	0.264	69	1.197	-0.097	4.166	0.855	0.004	80	12	396.2
	158964549	0.869	4.746	0.915	0.000	0.126	100	1.450	-0.108	4.531	0.953	0.000	100	13	938.8
	158984779	0.762	4.430	0.855	0.002	0.254	90	1.392	-0.133	4.266	0.917	0.003	91	10	465.4
	159005010	0.738	4.766	0.805	0.002	0.286	84	1.310	-0.101	4.603	0.883	0.001	92	13	771.9
	159025240	0.822	3.940	0.806	0.002	0.122	79	1.618	-0.139	3.699	0.881	0.002	88	12	423.8
	159085930	0.687	4.552	0.735	0.006	0.334	74	1.197	-0.093	4.453	0.838	0.004	83	13	555.0
	159146620	0.645	5.236	0.940	0.000	0.426	100	1.097	-0.084	5.068	0.974	0.000	100	13	888.0
	159166850	0.500	5.425	0.886	0.000	0.574	100	0.930	-0.080	5.265	0.948	0.000	100	12	747.7
	159227541	0.669	5.079	0.865	0.000	0.357	97	1.228	-0.091	4.814	0.920	0.001	98	14	882.5
	159247771	0.793	4.487	0.823	0.001	0.184	95	1.476	-0.112	4.177	0.894	0.002	97	13	669.4
	159389382	0.779	5.177	0.954	0.000	0.267	100	1.229	-0.084	5.010	0.979	0.000	100	15	1191.5
	159470302	0.613	5.064	0.769	0.004	0.428	92	1.093	-0.080	4.859	0.868	0.002	95	14	755.2
	159713063	0.592	5.185	0.844	0.001	0.439	97	1.018	-0.071	5.002	0.899	0.001	100	14	811.1
	159733294	0.720	4.912	0.871	0.001	0.312	98	1.307	-0.095	4.631	0.931	0.000	99	14	850.3
	160744799	0.912	3.647	0.853	0.002	0.007	96	1.810	-0.159	3.275	0.921	0.001	96	11	354.0
	160825720	0.788	5.736	0.966	0.000	0.262	100	1.234	-0.088	5.591	0.987	0.000	100	14	2038.1
	160866180	0.683	5.368	0.939	0.000	0.381	100	1.118	-0.073	5.170	0.970	0.000	100	15	1210.6
	160886410	0.796	5.021	0.939	0.000	0.218	100	1.397	-0.118	4.826	0.975	0.000	100	12	973.1
	160906640	0.667	5.428	0.958	0.000	0.401	100	1.081	-0.086	5.314	0.983	0.000	100	13	1059.3
	160947100	0.856	5.532	0.954	0.000	0.074	100	1.487	-0.117	5.298	0.981	0.000	100	13	1969.1
	160967330	0.715	5.661	0.964	0.000	0.349	100	1.094	-0.075	5.537	0.984	0.000	100	15	1607.8
	160987560	0.691	5.696	0.909	0.000	0.338	100	1.285	-0.117	5.503	0.952	0.000	100	11	1479.0
	161007791	0.644	5.066	0.869	0.002	0.417	93	1.028	-0.076	4.974	0.923	0.001	98	13	748.2
	161270782	0.880	5.279	0.946	0.000	0.012	100	1.561	-0.134	5.058	0.975	0.000	100	12	1529.0
	161311242	0.677	5.675	0.963	0.000	0.391	100	1.041	-0.081	5.593	0.983	0.000	100	13	1360.7
	161412393	0.446	5.405	0.865	0.010	0.630	76	0.729	-0.091	5.432	0.934	0.010	81	8	503.9
	161473083	0.772	4.662	0.905	0.000	0.252	100	1.248	-0.076	4.415	0.949	0.000	99	17	785.9
	161554003	0.576	5.167	0.948	0.000	0.499	100	0.929	-0.074	5.069	0.977	0.000	100	13	662.4
	178713055	1.124	3.817	0.858	0.001	-0.408	98	2.347	-0.242	3.433	0.931	0.001	99	10	612.2
	184349034	0.762	5.062	0.878	0.008	0.279	85	1.513	-0.228	5.113	0.953	0.006	84	7	640.4
	208027353	0.661	5.195	0.881	0.000	0.379	100	1.225	-0.095	4.939	0.942	0.000	100	13	940.0
	246515023	0.497	5.131	0.769	0.003	0.573	85	0.622	-0.025	5.117	0.836	0.004	94	25	664.8
	256789458	1.094	3.881	0.850	0.005	-0.379	87	2.211	-0.304	3.890	0.922	0.005	94	7	431.3
	275382046	1.169	3.744	0.890	0.010	-0.283	47	2.490	-0.480	4.196	0.952	0.012	56	5	331.2
	289996019	0.707	4.796	0.842	0.003	0.305	83	1.319	-0.144	4.731	0.900	0.005	89	9	564.2
	295137534	0.796	4.779	0.931	0.000	0.179	100	1.424	-0.131	4.605	0.969	0.000	100	11	720.5
	336497421	0.717	5.325	0.900	0.000	0.300	100	1.335	-0.128	5.149	0.953	0.000	99	10	1038.3
	368533040	0.536	5.386	0.928	0.000	0.540	100	0.847	-0.058	5.271	0.960	0.000	100	15	810.1
	370027359	0.622	5.204	0.932	0.000	0.448	100	1.050	-0.080	5.046	0.967	0.000	100	13	815.7
	370425937	0.693	4.551	0.928	0.000	0.354	100	1.166	-0.076	4.317	0.965	0.000	100	15	564.0
	375450439	0.636	5.175	0.906	0.000	0.417	100	1.112	-0.077	4.940	0.948	0.000	100	15	900.8
	404239096	0.657	5.484	0.975	0.000	0.418	100	0.925	-0.056	5.410	0.987	0.000	100	17	1191.7
	414519462	0.670	4.784	0.852	0.001	0.357	100	1.217	-0.088	4.514	0.918	0.001	100	14	661.7
	451588811	0.990	3.983	0.801	0.005	-0.120	81	1.952	-0.207	3.851	0.892	0.003	91	9	534.6

	465578759	0.606	4.343	0.828	0.002	0.441	100	0.984	-0.061	4.156	0.904	0.001	100	16	369.9
	492786515	0.692	4.673	0.903	0.000	0.292	100	1.323	-0.117	4.439	0.956	0.000	100	11	555.7
	508703490	0.879	4.178	0.845	0.001	0.065	100	1.899	-0.190	3.800	0.926	0.001	100	10	530.0
	516889361	0.720	5.048	0.942	0.005	0.350	73	0.490	0.051	5.156	0.973	0.006	78	-10	NaN
	517810313	0.531	5.201	0.903	0.001	0.544	100	0.939	-0.074	5.036	0.961	0.000	98	13	650.7
	550534656	0.558	4.219	0.970	0.009	0.524	6	-0.450	0.272	4.469	0.989	0.023	16	2	109.2
	553359145	0.600	4.749	0.840	0.002	0.444	97	1.118	-0.090	4.542	0.913	0.001	100	12	515.9
	604812005	0.779	5.493	0.968	0.000	0.267	100	1.188	-0.080	5.360	0.987	0.000	100	15	1589.2
	612472597	0.692	4.990	0.824	0.002	0.311	95	1.222	-0.087	4.752	0.888	0.002	97	14	861.8
	638754422	0.642	5.111	0.924	0.000	0.424	100	1.000	-0.063	4.962	0.959	0.000	100	16	831.4
	643185023	1.092	3.824	0.920	0.000	-0.313	99	1.937	-0.189	3.648	0.961	0.000	100	10	500.6
	650853796	0.750	4.531	0.836	0.004	0.258	89	1.313	-0.109	4.327	0.910	0.002	87	12	536.7
	658594300	1.156	3.883	0.897	0.004	-0.465	95	2.273	-0.338	3.924	0.962	0.003	96	7	396.7
	663835652	0.731	4.700	0.854	0.002	0.294	93	1.415	-0.137	4.504	0.928	0.001	95	10	597.1
	668248235	0.547	5.330	0.921	0.000	0.529	100	0.958	-0.077	5.177	0.966	0.000	100	13	766.1
	682102541	1.243	3.217	0.906	0.035	-0.381	21	4.155	-1.284	4.870	0.989	0.022	19	3	269.3
	682449369	0.663	5.168	0.894	0.000	0.371	100	1.222	-0.094	4.913	0.945	0.000	100	13	918.9
	686765762	0.678	5.042	0.961	0.000	0.386	100	1.050	-0.069	4.905	0.983	0.000	100	15	820.4
	706846339	1.525	3.723	0.926	0.025	-0.882	25	0.980	-0.056	4.512	0.987	0.026	37	17	562.0
	737052003	0.611	4.838	0.858	0.001	0.429	99	1.097	-0.086	4.637	0.919	0.000	99	13	563.3
	739574095	0.773	2.898	0.898	0.017	0.275	28	1.785	-0.410	3.439	0.965	0.020	33	4	72.3
	763395383	0.596	5.510	0.958	0.000	0.480	100	0.898	-0.059	5.412	0.978	0.000	100	15	1045.5
	763435843	0.581	5.310	0.868	0.001	0.434	100	1.148	-0.111	5.126	0.932	0.001	100	10	777.1
	763456073	0.638	4.915	0.844	0.001	0.373	100	1.246	-0.106	4.648	0.923	0.001	98	12	648.6
	763476303	0.591	4.480	0.781	0.006	0.471	73	0.908	-0.075	4.512	0.869	0.006	85	12	351.1
	763516763	0.863	4.785	0.934	0.000	0.080	100	1.552	-0.128	4.529	0.968	0.000	100	12	940.8
	763536994	0.687	5.093	0.909	0.000	0.359	100	1.223	-0.105	4.919	0.958	0.000	100	12	808.1
	763557224	0.917	3.362	0.777	0.007	0.034	67	1.710	-0.178	3.361	0.872	0.004	75	10	249.3
	763597684	0.544	5.707	0.938	0.000	0.535	100	0.928	-0.076	5.582	0.975	0.000	100	12	1077.3
	763638144	0.810	4.882	0.890	0.000	0.182	100	1.491	-0.115	4.572	0.942	0.000	100	13	996.7
	763698834	0.671	4.608	0.791	0.003	0.335	83	1.227	-0.103	4.466	0.882	0.001	92	12	534.6
	763719065	0.725	4.956	0.926	0.000	0.304	100	1.209	-0.078	4.716	0.959	0.000	100	16	918.2
	763759525	0.722	4.786	0.893	0.000	0.317	100	1.186	-0.075	4.557	0.942	0.000	100	16	773.2
	763820215	0.734	4.008	0.828	0.003	0.298	93	1.278	-0.097	3.797	0.901	0.002	94	13	333.9
	763840445	0.740	4.945	0.872	0.000	0.261	100	1.373	-0.102	4.632	0.932	0.000	100	13	926.1
	763860675	0.782	4.998	0.913	0.000	0.221	100	1.341	-0.110	4.817	0.958	0.000	100	12	925.7
	763880905	0.593	4.797	0.855	0.003	0.475	89	0.947	-0.080	4.748	0.916	0.003	95	12	466.2
	763901136	0.837	4.901	0.917	0.000	0.164	99	1.438	-0.098	4.619	0.956	0.000	100	15	1149.4
	763921366	0.783	4.055	0.791	0.002	0.215	71	1.342	-0.104	3.989	0.855	0.003	83	13	434.7
	763982056	0.508	5.385	0.902	0.000	0.565	99	0.789	-0.053	5.289	0.940	0.000	100	15	760.0
	764002286	0.679	4.885	0.851	0.002	0.347	98	1.233	-0.102	4.671	0.927	0.001	97	12	671.8
	764042746	0.562	5.026	0.687	0.010	0.511	60	0.794	-0.062	5.154	0.809	0.006	86	13	591.5
	764062976	0.733	4.900	0.902	0.000	0.295	100	1.335	-0.101	4.626	0.952	0.000	100	13	839.5
	764083206	0.612	5.239	0.884	0.000	0.439	100	1.091	-0.088	5.053	0.944	0.000	99	12	816.5
	764143897	0.609	5.050	0.859	0.000	0.445	100	1.161	-0.089	4.777	0.926	0.000	100	13	736.0
	764184357	0.700	4.842	0.874	0.000	0.346	99	1.243	-0.088	4.585	0.926	0.001	100	14	757.3
	764224817	0.635	5.509	0.954	0.000	0.438	100	1.016	-0.071	5.367	0.981	0.000	100	14	1160.1
	764245047	0.700	3.966	0.815	0.003	0.317	88	1.386	-0.125	3.690	0.900	0.001	86	11	281.3
	764285508	0.633	5.354	0.944	0.000	0.437	100	1.176	-0.138	5.294	0.980	0.000	100	9	761.9
	764305738	0.662	5.387	0.956	0.000	0.407	100	1.073	-0.076	5.235	0.983	0.000	100	14	1091.6
	764325968	0.752	5.464	0.948	0.000	0.276	100	1.252	-0.093	5.279	0.977	0.000	100	13	1450.6
	764346198	0.623	5.423	0.908	0.000	0.443	100	1.093	-0.079	5.209	0.958	0.000	100	14	1080.0
	764366428	0.630	4.400	0.820	0.002	0.413	84	1.154	-0.105	4.287	0.890	0.004	91	11	363.8
	764447348	0.714	4.927	0.947	0.000	0.344	100	1.112	-0.078	4.797	0.973	0.000	100	14	763.2
	764467579	0.801	4.413	0.776	0.004	0.190	75	1.460	-0.134	4.331	0.863	0.004	88	11	579.2

	764487809	0.670	5.142	0.953	0.000	0.399	100	1.047	-0.070	5.002	0.978	0.000	100	15	885.1
	764508039	0.489	5.048	0.780	0.006	0.582	90	0.817	-0.067	4.963	0.885	0.003	94	12	487.6
	764588959	0.710	4.642	0.919	0.000	0.336	100	1.165	-0.080	4.454	0.956	0.000	100	14	603.0
	764649650	0.658	4.956	0.895	0.000	0.387	100	1.149	-0.079	4.713	0.945	0.000	100	15	766.0
	764669880	0.634	5.548	0.910	0.000	0.429	100	1.113	-0.089	5.371	0.953	0.000	100	12	1173.2
	764710340	0.803	3.084	0.841	0.013	0.242	46	1.427	-0.231	3.438	0.925	0.011	58	6	100.6
	764750800	0.608	5.364	0.936	0.000	0.465	100	0.950	-0.067	5.252	0.967	0.000	100	14	914.0
	764811490	0.709	4.787	0.922	0.000	0.342	100	1.124	-0.069	4.586	0.957	0.000	99	16	735.5
	764831721	0.779	5.090	0.948	0.000	0.257	100	1.306	-0.110	4.944	0.979	0.000	100	12	964.5
	764872181	0.526	5.139	0.811	0.003	0.532	90	0.864	-0.071	5.074	0.889	0.003	96	12	583.5
	764892411	0.610	5.548	0.966	0.000	0.468	100	0.920	-0.058	5.433	0.984	0.000	100	16	1165.0
	764953101	0.732	5.275	0.949	0.000	0.320	100	1.190	-0.101	5.171	0.977	0.000	100	12	1004.3
	765013792	0.702	4.609	0.823	0.002	0.312	91	1.311	-0.103	4.343	0.905	0.001	91	13	581.2
	765034022	0.751	4.812	0.912	0.000	0.279	100	1.283	-0.090	4.570	0.954	0.000	100	14	813.2
	765074482	0.734	5.267	0.951	0.000	0.297	100	1.165	-0.080	5.108	0.976	0.000	100	15	1164.8
	765094712	0.593	5.411	0.944	0.000	0.480	100	0.990	-0.078	5.282	0.975	0.000	100	13	905.1
	765155402	0.644	5.338	0.952	0.000	0.429	100	1.032	-0.072	5.194	0.980	0.000	100	14	999.8
	765195863	0.532	5.273	0.805	0.002	0.518	99	0.972	-0.071	5.063	0.896	0.001	100	14	756.7
	765216093	0.605	5.413	0.949	0.000	0.469	100	0.965	-0.067	5.279	0.976	0.000	100	14	979.5
	765256553	0.720	4.847	0.840	0.003	0.299	88	1.380	-0.138	4.719	0.916	0.001	95	10	677.2
	765276783	0.559	5.150	0.935	0.000	0.519	100	0.941	-0.071	5.008	0.969	0.000	100	13	663.0
	765317243	0.739	3.905	0.797	0.009	0.276	56	1.227	-0.121	4.022	0.880	0.005	67	10	280.9
	765337473	0.697	4.705	0.824	0.001	0.285	100	1.370	-0.107	4.364	0.908	0.000	99	13	653.7
	765377934	0.576	5.395	0.953	0.000	0.504	100	0.986	-0.104	5.350	0.983	0.000	100	9	719.7
	765560005	0.583	5.448	0.937	0.000	0.488	100	0.996	-0.081	5.314	0.973	0.000	100	12	911.5
	809635352	0.531	5.137	0.741	0.006	0.530	81	0.961	-0.077	5.025	0.856	0.003	92	13	660.9
	863126187	0.617	5.339	0.896	0.001	0.440	99	0.982	-0.076	5.233	0.948	0.000	99	13	869.5
	892969023	0.582	5.202	0.862	0.001	0.487	99	0.906	-0.057	5.067	0.929	0.001	99	16	781.2
	937495960	0.821	4.967	0.983	0.000	0.219	100	1.128	-0.068	4.897	0.992	0.000	100	17	1029.4
	953045535	0.700	4.854	0.864	0.001	0.330	97	1.239	-0.095	4.628	0.927	0.000	97	13	710.8
	970836795	0.680	4.804	0.793	0.003	0.297	96	1.427	-0.128	4.492	0.902	0.002	98	11	669.7
	**Mean**	0.721	4.951	0.881	0.001	0.301	99	1.386	-0.136	4.744	0.942	0.001	99	12	790.7
	**Std. Err.**	0.072	0.089	0.020	0.000	0.096	1	0.204	0.045	0.064	0.013	0.000	1	0	26.6
q=2	132902142	0.950	3.053	0.867	0.004	0.012	75	2.009	-0.273	2.965	0.943	0.003	76	7	143.4
	147406386	0.526	3.141	0.738	0.008	0.526	54	1.218	-0.157	3.244	0.848	0.004	79	8	91.6
	158013734	0.685	3.675	0.913	0.000	0.369	93	1.111	-0.073	3.491	0.950	0.001	94	15	223.8
	158114885	0.566	3.299	0.795	0.004	0.454	67	0.995	-0.084	3.296	0.867	0.003	77	12	116.1
	158155345	0.645	3.112	0.836	0.005	0.399	45	0.823	-0.072	3.453	0.862	0.007	63	11	102.7
	158216035	0.732	3.572	0.873	0.002	0.301	89	1.259	-0.104	3.426	0.929	0.002	88	12	200.7
	158236265	0.479	3.058	0.754	0.007	0.504	49	1.055	-0.128	3.208	0.827	0.006	76	8	79.4
	158276726	0.771	3.231	0.893	0.000	0.233	100	1.309	-0.087	2.965	0.939	0.000	100	15	183.5
	158398106	0.396	4.439	0.787	0.005	0.677	92	0.870	-0.089	4.270	0.895	0.001	93	10	218.6
	158418336	0.497	4.371	0.871	0.001	0.575	100	0.916	-0.082	4.232	0.930	0.001	99	11	252.0
	158438567	0.577	3.956	0.890	0.001	0.488	99	0.953	-0.069	3.802	0.937	0.001	97	14	212.0
	158458797	0.586	3.589	0.918	0.000	0.486	100	0.961	-0.063	3.418	0.953	0.000	100	15	159.8
	158479027	0.687	4.280	0.926	0.000	0.372	100	1.099	-0.081	4.146	0.957	0.000	100	14	369.6
	158499257	0.799	3.554	0.904	0.000	0.211	99	1.536	-0.163	3.388	0.960	0.000	99	9	199.5
	158721788	0.525	3.939	0.862	0.002	0.545	90	1.016	-0.104	3.821	0.930	0.001	91	10	167.3
	158742018	0.535	3.861	0.839	0.003	0.538	77	0.888	-0.061	3.730	0.896	0.002	82	15	184.2
	158802708	0.561	3.678	0.843	0.003	0.506	88	1.081	-0.099	3.513	0.914	0.002	91	11	151.3
	158822939	0.464	4.279	0.797	0.006	0.610	67	0.822	-0.076	4.203	0.884	0.003	73	11	207.2
	158883629	0.062	3.507	0.639	0.016	0.873	40	0.794	-0.132	3.374	0.786	0.006	76	6	54.7
	158924089	0.660	4.074	0.806	0.005	0.388	83	1.146	-0.096	3.950	0.877	0.004	86	12	281.9
	158944319	0.223	2.929	0.756	0.006	0.729	52	0.573	-0.070	2.904	0.826	0.007	69	8	34.4
	158964549	0.832	3.458	0.873	0.001	0.178	96	1.330	-0.094	3.286	0.921	0.001	97	14	240.0

	158984779	0.688	2.691	0.786	0.007	0.359	58	1.387	-0.176	2.823	0.872	0.005	72	8	73.7
	159005010	0.317	3.439	0.684	0.011	0.643	50	0.885	-0.106	3.344	0.811	0.005	75	8	76.2
	159025240	0.506	2.599	0.742	0.006	0.500	59	1.061	-0.105	2.549	0.812	0.006	80	10	51.4
	159085930	-0.035	3.582	0.705	0.008	0.902	50	0.565	-0.097	3.399	0.788	0.005	75	6	46.0
	159146620	0.653	3.815	0.915	0.000	0.416	98	1.109	-0.086	3.657	0.955	0.000	99	13	218.3
	159166850	0.431	3.629	0.759	0.006	0.629	54	0.945	-0.115	3.694	0.849	0.004	77	8	114.7
	159227541	0.699	3.267	0.772	0.003	0.339	63	1.345	-0.121	3.174	0.866	0.002	75	11	158.7
	159247771	0.722	2.711	0.814	0.003	0.316	68	1.252	-0.101	2.670	0.869	0.002	80	12	96.1
	159389382	0.740	3.975	0.929	0.000	0.308	100	1.216	-0.089	3.798	0.962	0.000	100	14	319.4
	159470302	0.533	3.243	0.682	0.010	0.521	44	0.806	-0.070	3.430	0.794	0.006	66	11	98.7
	159713063	0.628	3.135	0.754	0.008	0.394	52	1.112	-0.102	3.171	0.841	0.004	70	11	111.6
	159733294	0.626	3.142	0.832	0.002	0.432	80	1.134	-0.091	3.024	0.894	0.002	90	12	115.2
	160744799	0.719	2.358	0.832	0.002	0.302	80	1.350	-0.121	2.209	0.889	0.002	87	11	61.2
	160825720	0.708	4.916	0.942	0.000	0.344	100	1.189	-0.095	4.759	0.974	0.000	100	13	720.5
	160866180	0.471	4.438	0.756	0.006	0.603	73	1.050	-0.105	4.271	0.860	0.003	83	10	282.3
	160886410	0.780	3.516	0.863	0.002	0.207	83	1.223	-0.096	3.467	0.907	0.004	90	13	211.0
	160906640	0.503	4.031	0.808	0.004	0.567	85	1.112	-0.132	3.920	0.896	0.004	91	8	177.2
	160947100	0.767	4.744	0.920	0.000	0.211	100	1.390	-0.116	4.513	0.960	0.000	100	12	717.2
	160967330	0.680	4.688	0.947	0.000	0.382	100	1.034	-0.070	4.573	0.969	0.000	100	15	560.4
	160987560	0.597	4.263	0.703	0.013	0.444	41	1.562	-0.226	4.416	0.830	0.007	63	7	355.8
	161007791	0.546	3.407	0.803	0.005	0.510	57	0.803	-0.077	3.604	0.857	0.005	80	10	108.6
	161270782	0.702	4.281	0.796	0.004	0.271	88	1.467	-0.155	4.083	0.886	0.003	92	9	370.0
	161311242	0.649	4.406	0.902	0.002	0.412	87	1.161	-0.116	4.319	0.948	0.001	89	10	342.1
	161412393	-0.116	4.220	0.827	0.015	1.039	46	-0.282	0.057	4.172	0.911	0.016	64	5	54.7
	161473083	0.700	3.198	0.868	0.001	0.352	77	1.077	-0.071	3.133	0.904	0.002	86	15	146.5
	161554003	0.489	3.738	0.806	0.005	0.565	75	0.873	-0.085	3.679	0.879	0.005	82	10	126.4
	178713055	1.059	2.298	0.837	0.003	-0.173	78	1.794	-0.160	2.198	0.895	0.002	86	11	113.8
	184349034	0.779	3.136	0.878	0.009	0.273	51	1.571	-0.263	3.298	0.949	0.006	59	6	93.3
	208027353	0.711	3.371	0.804	0.006	0.330	63	1.213	-0.106	3.450	0.877	0.003	80	11	179.2
	246515023	0.004	4.290	0.666	0.010	0.950	56	-0.336	0.051	4.413	0.780	0.005	79	7	61.4
	256789458	0.823	2.246	0.841	0.010	0.105	45	1.704	-0.264	2.513	0.908	0.009	62	6	54.0
	275382046	0.983	1.881	0.888	0.014	0.006	24	2.437	-0.575	2.788	0.946	0.015	34	4	48.0
	289996019	0.706	2.845	0.793	0.008	0.300	52	1.370	-0.172	2.893	0.877	0.008	60	8	79.0
	295137534	0.618	3.710	0.833	0.004	0.430	78	1.116	-0.111	3.600	0.899	0.003	82	10	158.4
	336497421	0.734	3.498	0.805	0.006	0.261	66	1.371	-0.148	3.461	0.887	0.005	76	9	171.5
	368533040	0.344	3.925	0.775	0.008	0.710	51	0.772	-0.087	3.862	0.863	0.005	59	9	118.5
	370027359	0.541	3.886	0.854	0.002	0.536	85	1.074	-0.098	3.674	0.932	0.001	83	11	175.9
	370425937	0.599	3.068	0.895	0.000	0.464	99	0.995	-0.064	2.871	0.939	0.000	99	16	100.6
	375450439	0.571	3.704	0.821	0.002	0.493	83	1.003	-0.076	3.576	0.880	0.002	90	13	174.6
	404239096	0.660	4.081	0.949	0.001	0.408	100	0.929	-0.056	4.007	0.970	0.001	100	17	294.9
	414519462	0.611	3.200	0.821	0.004	0.445	80	1.042	-0.075	3.065	0.888	0.002	86	14	117.0
	451588811	0.481	2.665	0.765	0.008	0.467	55	1.334	-0.195	2.711	0.839	0.007	75	7	51.4
	465578759	0.496	2.879	0.784	0.003	0.573	76	0.647	-0.037	2.922	0.854	0.003	93	18	62.5
	492786515	0.670	2.819	0.762	0.007	0.351	53	1.275	-0.147	2.987	0.855	0.004	79	9	87.3
	508703490	0.791	2.901	0.811	0.003	0.235	83	1.641	-0.168	2.699	0.894	0.002	90	10	121.3
	516889361	0.636	3.640	0.927	0.005	0.440	50	-0.021	0.183	3.708	0.966	0.007	63	0	43.5
	517810313	0.466	3.519	0.791	0.006	0.599	70	0.925	-0.096	3.469	0.881	0.004	81	10	103.9
	550534656	0.433	2.456	0.955	0.016	0.647	8	-1.495	0.536	2.889	0.988	0.024	13	3	17.3
	553359145	0.575	2.868	0.817	0.002	0.487	74	1.031	-0.090	2.837	0.874	0.003	86	11	74.8
	604812005	0.680	4.583	0.902	0.001	0.363	98	1.104	-0.085	4.457	0.941	0.001	99	13	487.7
	612472597	0.601	3.198	0.749	0.008	0.346	45	1.204	-0.126	3.322	0.818	0.004	69	10	125.6
	638754422	0.565	3.603	0.870	0.002	0.509	80	0.887	-0.065	3.558	0.918	0.001	88	14	146.3
	643185023	0.905	2.670	0.906	0.001	0.043	93	1.560	-0.147	2.538	0.950	0.001	94	11	105.6
	650853796	0.620	2.634	0.824	0.006	0.401	60	0.991	-0.096	2.759	0.869	0.006	75	10	59.3
	658594300	0.979	2.635	0.869	0.010	-0.054	81	1.791	-0.252	2.700	0.949	0.006	83	7	83.5

	663835652	0.517	2.965	0.770	0.006	0.507	64	1.291	-0.178	3.038	0.860	0.004	85	7	74.2
	668248235	0.537	3.294	0.820	0.005	0.538	52	1.022	-0.109	3.382	0.881	0.006	67	9	104.2
	682102541	1.122	1.438	0.911	0.032	-0.177	6	2.687	-0.945	3.427	0.990	0.021	17	3	34.7
	682449369	0.591	3.901	0.796	0.004	0.459	88	1.102	-0.089	3.714	0.876	0.002	92	12	217.4
	686765762	0.546	3.602	0.872	0.003	0.526	95	0.848	-0.055	3.474	0.928	0.002	93	15	141.1
	706846339	1.113	2.615	0.904	0.036	-0.185	16	2.342	-0.607	3.410	0.992	0.015	15	4	68.7
	737052003	0.600	3.026	0.754	0.007	0.443	54	1.169	-0.131	3.169	0.825	0.006	78	9	95.4
	739574095	0.382	1.950	0.902	0.017	0.656	34	0.732	-0.144	2.141	0.957	0.026	37	5	13.5
	763395383	0.599	4.027	0.897	0.001	0.462	92	0.952	-0.071	3.921	0.937	0.001	93	13	231.3
	763435843	0.565	3.743	0.789	0.005	0.475	74	1.191	-0.131	3.636	0.891	0.003	82	9	159.4
	763456073	0.736	2.862	0.782	0.005	0.273	53	1.324	-0.140	3.059	0.849	0.005	74	9	110.9
	763476303	0.064	3.243	0.746	0.011	0.835	37	0.500	-0.086	3.252	0.836	0.007	68	6	37.7
	763516763	0.806	3.698	0.907	0.001	0.172	99	1.432	-0.115	3.453	0.950	0.000	98	12	278.0
	763536994	0.603	3.437	0.794	0.005	0.445	75	1.133	-0.117	3.384	0.881	0.004	87	10	124.5
	763557224	0.166	2.698	0.720	0.012	0.730	51	0.894	-0.156	2.726	0.839	0.007	65	6	29.8
	763597684	0.536	3.837	0.802	0.005	0.535	54	1.045	-0.123	3.929	0.877	0.004	74	9	167.6
	763638144	0.698	3.604	0.843	0.002	0.324	92	1.172	-0.083	3.433	0.890	0.002	95	14	212.6
	763698834	0.379	2.895	0.746	0.006	0.601	54	0.864	-0.103	2.980	0.817	0.006	79	8	52.0
	763719065	0.621	3.846	0.814	0.003	0.412	84	1.152	-0.086	3.603	0.885	0.001	84	13	230.4
	763759525	0.633	3.141	0.825	0.003	0.425	77	1.041	-0.073	3.026	0.882	0.004	84	14	115.3
	763820215	0.644	2.536	0.853	0.003	0.412	76	1.005	-0.070	2.440	0.901	0.003	82	14	60.8
	763840445	0.776	3.304	0.845	0.002	0.241	78	1.324	-0.101	3.224	0.895	0.002	88	13	200.8
	763860675	0.820	3.310	0.816	0.005	0.161	79	1.384	-0.121	3.238	0.899	0.003	86	11	185.8
	763880905	0.286	2.951	0.773	0.007	0.719	45	0.713	-0.111	3.183	0.843	0.008	70	6	44.7
	763901136	0.788	3.670	0.894	0.001	0.240	90	1.229	-0.073	3.483	0.929	0.001	92	17	305.3
	763921366	0.232	2.933	0.773	0.003	0.697	57	0.577	-0.067	2.911	0.825	0.004	73	9	35.6
	763982056	0.484	3.753	0.804	0.004	0.570	68	0.576	-0.026	3.794	0.859	0.005	78	22	149.4
	764002286	0.810	2.351	0.842	0.004	0.159	41	1.241	-0.142	3.003	0.855	0.005	73	9	85.7
	764042746	-0.633	5.521	0.590	0.019	1.299	26	0.576	-0.164	4.555	0.764	0.007	82	4	110.1
	764062976	0.594	3.745	0.775	0.003	0.458	84	1.242	-0.116	3.538	0.872	0.002	92	11	188.9
	764083206	0.754	2.863	0.835	0.002	0.282	48	1.126	-0.120	3.352	0.856	0.005	75	9	114.8
	764143897	0.581	3.505	0.818	0.003	0.482	82	1.097	-0.087	3.313	0.882	0.002	87	13	147.3
	764184357	0.588	3.525	0.799	0.003	0.478	84	1.116	-0.093	3.376	0.867	0.003	93	12	152.7
	764224817	0.529	4.320	0.907	0.000	0.547	100	0.863	-0.062	4.196	0.946	0.000	100	14	271.1
	764245047	0.461	2.388	0.769	0.006	0.545	50	0.913	-0.105	2.470	0.841	0.006	68	9	34.2
	764285508	0.667	4.231	0.933	0.001	0.389	100	1.152	-0.123	4.177	0.968	0.001	100	9	270.2
	764305738	0.659	3.965	0.925	0.000	0.401	99	1.096	-0.082	3.810	0.957	0.001	100	13	258.1
	764325968	0.708	4.317	0.926	0.000	0.328	100	1.218	-0.095	4.128	0.963	0.000	100	13	411.2
	764346198	0.495	4.021	0.768	0.005	0.570	69	1.037	-0.104	3.967	0.855	0.003	82	10	202.9
	764366428	0.555	3.107	0.763	0.008	0.494	58	1.053	-0.114	3.145	0.851	0.006	79	9	84.0
	764447348	0.515	3.739	0.850	0.003	0.562	76	0.810	-0.062	3.685	0.890	0.005	82	13	141.7
	764467579	0.096	3.692	0.684	0.011	0.796	43	0.983	-0.161	3.374	0.794	0.007	82	6	64.8
	764487809	0.588	3.748	0.882	0.001	0.485	99	1.055	-0.088	3.582	0.934	0.001	100	12	172.5
	764508039	0.580	3.258	0.817	0.005	0.485	55	0.610	-0.031	3.486	0.875	0.004	76	19	108.4
	764588959	0.631	3.439	0.867	0.001	0.430	98	1.055	-0.075	3.267	0.924	0.000	98	14	148.5
	764649650	0.586	3.138	0.731	0.006	0.455	52	1.191	-0.129	3.281	0.824	0.003	83	9	113.8
	764669880	0.815	3.105	0.821	0.007	0.191	27	1.427	-0.199	3.987	0.838	0.005	66	7	214.6
	764710340	0.502	2.275	0.811	0.018	0.533	42	1.225	-0.249	2.567	0.914	0.016	62	5	26.9
	764750800	0.596	3.309	0.807	0.006	0.470	58	0.976	-0.096	3.387	0.864	0.006	72	10	107.4
	764811490	0.621	3.299	0.848	0.002	0.437	96	1.073	-0.075	3.082	0.917	0.001	95	14	128.7
	764831721	0.759	3.337	0.879	0.003	0.267	90	1.363	-0.126	3.175	0.945	0.001	90	11	157.6
	764872181	0.378	3.181	0.744	0.008	0.608	35	0.772	-0.112	3.494	0.826	0.008	61	7	67.4
	764892411	0.576	4.361	0.936	0.000	0.498	100	0.942	-0.068	4.225	0.966	0.000	100	14	316.7
	764953101	0.688	3.914	0.896	0.002	0.358	100	1.213	-0.114	3.775	0.946	0.001	98	11	227.7
	765013792	0.452	3.046	0.769	0.005	0.538	57	1.096	-0.130	3.100	0.842	0.003	82	8	77.1

	765034022	0.674	3.539	0.865	0.001	0.371	96	1.093	-0.072	3.356	0.913	0.001	97	15	189.6
	765074482	0.672	4.071	0.880	0.001	0.362	98	1.107	-0.082	3.921	0.927	0.001	99	14	297.4
	765094712	0.548	3.926	0.881	0.004	0.521	89	0.931	-0.076	3.812	0.931	0.002	90	12	183.4
	765155402	0.537	4.116	0.839	0.003	0.532	94	1.057	-0.098	3.937	0.923	0.001	95	11	220.8
	765195863	0.354	3.437	0.718	0.012	0.619	32	0.960	-0.137	3.831	0.795	0.006	74	7	114.4
	765216093	0.516	4.248	0.836	0.002	0.556	93	0.998	-0.090	4.077	0.911	0.001	94	11	238.8
	765256553	0.626	3.270	0.785	0.008	0.411	60	1.316	-0.167	3.418	0.874	0.005	75	8	123.6
	765276783	0.455	3.723	0.783	0.006	0.617	82	0.957	-0.095	3.554	0.893	0.002	84	10	121.9
	765317243	0.225	2.884	0.764	0.008	0.739	58	0.688	-0.098	2.856	0.847	0.007	67	7	33.3
	765337473	0.685	2.559	0.765	0.005	0.305	49	1.272	-0.131	2.836	0.845	0.002	74	10	86.0
	765377934	0.564	4.020	0.913	0.002	0.514	96	1.048	-0.122	3.961	0.962	0.001	95	9	175.0
	765560005	0.528	3.755	0.820	0.005	0.533	83	1.043	-0.107	3.650	0.907	0.002	89	10	145.6
	809635352	0.476	3.631	0.766	0.004	0.598	51	0.725	-0.065	3.840	0.829	0.004	82	11	130.0
	863126187	0.764	3.506	0.822	0.004	0.254	61	1.099	-0.097	3.609	0.890	0.003	76	11	177.8
	892969023	0.274	3.653	0.750	0.010	0.714	45	0.539	-0.065	3.762	0.827	0.006	75	8	78.8
	937495960	0.652	3.818	0.897	0.002	0.413	98	0.942	-0.062	3.737	0.945	0.001	96	15	211.5
	953045535	0.614	3.038	0.756	0.009	0.395	41	1.158	-0.130	3.292	0.832	0.005	68	9	106.1
	970836795	0.594	2.599	0.757	0.008	0.375	37	1.302	-0.173	3.099	0.831	0.004	85	8	83.3
	**Mean**	0.682	3.292	0.828	0.004	0.340	72	1.333	-0.147	3.249	0.902	0.003	82	10	158.0
	**Std. Err.**	0.096	0.138	0.039	0.002	0.114	8	0.230	0.046	0.109	0.026	0.001	4	0	9.3
q=3	132902142	0.903	2.346	0.883	0.004	0.102	63	1.675	-0.217	2.422	0.936	0.003	70	8	64.8
	147406386	0.269	2.876	0.699	0.013	0.740	52	1.033	-0.154	2.807	0.821	0.007	80	7	42.1
	158013734	0.670	3.178	0.929	0.000	0.390	93	0.951	-0.049	3.072	0.947	0.001	95	19	139.2
	158114885	0.429	2.874	0.790	0.005	0.588	70	0.757	-0.064	2.853	0.859	0.004	79	12	52.7
	158155345	0.403	2.955	0.790	0.009	0.613	49	0.696	-0.070	3.000	0.853	0.007	63	10	49.6
	158216035	0.727	2.884	0.881	0.002	0.315	78	1.194	-0.100	2.824	0.922	0.002	83	12	98.8
	158236265	0.248	2.825	0.743	0.007	0.712	48	0.881	-0.131	2.845	0.821	0.006	75	7	38.3
	158276726	0.741	2.740	0.880	0.000	0.277	98	1.199	-0.075	2.525	0.923	0.000	99	16	105.1
	158398106	0.361	3.819	0.719	0.009	0.710	62	0.867	-0.104	3.748	0.839	0.004	83	8	112.2
	158418336	0.464	3.718	0.855	0.002	0.608	95	0.827	-0.069	3.580	0.919	0.002	91	12	122.4
	158438567	0.525	3.436	0.879	0.001	0.545	97	0.790	-0.049	3.334	0.915	0.001	96	16	114.8
	158458797	0.539	3.084	0.907	0.000	0.536	100	0.871	-0.056	2.933	0.942	0.000	100	16	86.0
	158479027	0.613	3.765	0.898	0.000	0.455	100	0.997	-0.076	3.640	0.938	0.000	100	13	184.3
	158499257	0.631	3.143	0.811	0.006	0.420	93	1.450	-0.186	2.993	0.925	0.002	97	8	92.2
	158721788	0.491	3.223	0.830	0.004	0.585	70	1.005	-0.121	3.234	0.899	0.004	84	8	77.8
	158742018	0.489	3.373	0.830	0.003	0.587	74	0.822	-0.057	3.251	0.889	0.001	79	14	101.0
	158802708	0.505	3.042	0.833	0.003	0.569	82	1.015	-0.099	2.904	0.906	0.002	88	10	70.1
	158822939	0.482	3.471	0.818	0.003	0.596	55	0.714	-0.064	3.526	0.864	0.005	71	11	93.4
	158883629	-0.108	3.139	0.661	0.013	1.003	40	0.608	-0.119	2.905	0.785	0.006	76	5	26.9
	158924089	0.669	3.343	0.810	0.003	0.389	66	0.998	-0.078	3.379	0.873	0.003	76	13	138.5
	158944319	0.053	2.667	0.732	0.009	0.884	58	0.320	-0.052	2.635	0.820	0.007	71	6	18.1
	158964549	0.759	2.978	0.856	0.003	0.277	93	1.195	-0.083	2.840	0.912	0.002	95	14	124.6
	158984779	0.525	2.319	0.766	0.009	0.524	52	1.240	-0.182	2.496	0.853	0.007	74	7	38.0
	159005010	0.101	3.120	0.702	0.007	0.819	45	0.677	-0.097	2.933	0.802	0.005	76	7	35.6
	159025240	0.348	2.270	0.713	0.009	0.661	59	0.786	-0.083	2.233	0.796	0.007	80	9	24.9
	159085930	-0.204	3.257	0.705	0.006	1.043	57	0.251	-0.069	3.044	0.783	0.006	81	4	22.6
	159146620	0.638	3.258	0.912	0.001	0.433	98	1.073	-0.080	3.087	0.956	0.001	97	13	121.3
	159166850	0.271	3.156	0.731	0.008	0.761	46	0.736	-0.105	3.250	0.821	0.008	74	7	51.8
	159227541	0.519	2.947	0.726	0.007	0.517	63	1.195	-0.119	2.793	0.839	0.004	78	10	77.5
	159247771	0.568	2.398	0.797	0.004	0.480	72	0.984	-0.077	2.322	0.857	0.003	81	13	46.8
	159389382	0.724	3.458	0.924	0.000	0.327	100	1.191	-0.087	3.285	0.958	0.000	100	14	183.5
	159470302	0.316	3.047	0.643	0.017	0.703	35	0.636	-0.077	3.199	0.770	0.009	64	8	49.5
	159713063	0.445	2.733	0.741	0.007	0.558	44	1.001	-0.114	2.806	0.803	0.008	74	9	53.3
	159733294	0.550	2.637	0.832	0.002	0.516	78	0.963	-0.075	2.544	0.885	0.002	88	13	56.8
	160744799	0.602	2.034	0.800	0.004	0.445	76	1.184	-0.112	1.895	0.871	0.002	85	11	33.2

	160825720	0.655	4.356	0.916	0.000	0.398	100	1.090	-0.085	4.215	0.953	0.000	100	13	365.0
	160866180	0.434	3.672	0.741	0.007	0.641	48	0.992	-0.116	3.740	0.818	0.006	73	9	130.9
	160886410	0.710	2.921	0.848	0.004	0.290	75	1.052	-0.072	2.854	0.907	0.003	78	15	102.0
	160906640	0.470	3.288	0.768	0.008	0.602	58	1.128	-0.153	3.280	0.868	0.006	78	7	81.9
	160947100	0.727	4.204	0.902	0.000	0.274	99	1.293	-0.107	4.006	0.942	0.001	100	12	379.5
	160967330	0.680	4.122	0.950	0.000	0.383	100	0.976	-0.058	4.025	0.969	0.000	100	17	330.4
	160987560	0.187	4.279	0.667	0.019	0.773	29	1.519	-0.270	4.107	0.816	0.007	59	6	183.8
	161007791	0.402	3.023	0.790	0.005	0.638	57	0.635	-0.064	3.128	0.849	0.006	79	10	51.9
	161270782	0.747	3.362	0.777	0.007	0.243	56	1.562	-0.185	3.375	0.870	0.003	73	8	171.1
	161311242	0.632	3.689	0.881	0.002	0.431	79	1.115	-0.117	3.675	0.923	0.003	87	10	160.2
	161412393	-0.260	3.708	0.827	0.015	1.132	49	-0.564	0.105	3.612	0.913	0.015	64	5	25.2
	161473083	0.633	2.693	0.869	0.001	0.432	73	0.923	-0.059	2.696	0.893	0.003	84	16	74.8
	161554003	0.510	2.973	0.792	0.009	0.543	52	0.816	-0.077	3.022	0.870	0.006	62	11	62.1
	178713055	0.947	1.898	0.834	0.005	-0.001	77	1.334	-0.098	1.971	0.877	0.005	89	14	61.5
	184349034	0.595	2.744	0.863	0.012	0.446	52	1.243	-0.202	2.794	0.938	0.010	60	6	45.1
	208027353	0.589	2.883	0.785	0.007	0.457	60	1.051	-0.099	2.983	0.857	0.004	79	11	82.7
	246515023	-0.144	3.950	0.676	0.008	1.049	53	-0.614	0.077	4.084	0.785	0.005	79	8	30.7
	256789458	0.705	1.756	0.874	0.005	0.277	39	1.133	-0.159	2.126	0.912	0.008	55	7	25.0
	275382046	0.594	1.825	0.902	0.014	0.363	20	1.930	-0.468	2.347	0.944	0.017	31	4	23.4
	289996019	0.506	2.522	0.769	0.011	0.500	56	1.143	-0.147	2.436	0.865	0.009	63	8	38.0
	295137534	0.571	3.183	0.833	0.003	0.493	70	0.984	-0.096	3.130	0.894	0.003	77	10	84.8
	336497421	0.670	2.802	0.789	0.010	0.353	61	1.221	-0.130	2.788	0.876	0.006	70	9	74.1
	368533040	0.194	3.495	0.745	0.008	0.831	57	0.568	-0.072	3.383	0.834	0.008	66	8	53.9
	370027359	0.510	3.344	0.861	0.001	0.572	77	0.993	-0.093	3.195	0.923	0.002	81	11	94.5
	370425937	0.563	2.534	0.890	0.001	0.505	99	0.901	-0.054	2.367	0.930	0.001	99	17	54.4
	375450439	0.540	3.105	0.799	0.003	0.529	77	0.937	-0.073	3.019	0.864	0.003	87	13	88.1
	404239096	0.678	3.422	0.952	0.001	0.388	98	0.889	-0.046	3.382	0.968	0.001	100	19	167.4
	414519462	0.575	2.686	0.838	0.003	0.492	77	0.919	-0.061	2.584	0.898	0.001	83	15	63.7
	451588811	0.243	2.464	0.775	0.005	0.703	53	0.944	-0.154	2.398	0.845	0.006	70	6	23.8
	465578759	0.451	2.430	0.767	0.004	0.622	73	0.545	-0.028	2.503	0.845	0.003	91	19	35.6
	492786515	0.627	2.115	0.761	0.008	0.412	39	1.209	-0.159	2.515	0.838	0.005	73	8	43.0
	508703490	0.713	2.496	0.810	0.004	0.340	81	1.458	-0.147	2.315	0.888	0.003	87	10	67.0
	516889361	0.634	3.034	0.927	0.005	0.442	43	-0.120	0.204	3.137	0.963	0.008	59	1	27.7
	517810313	0.339	3.048	0.765	0.007	0.703	63	0.803	-0.094	2.979	0.860	0.005	79	9	49.2
	550534656	0.427	1.941	0.980	0.004	0.654	8	0.692	-0.110	2.016	0.993	0.014	8	6	13.5
	553359145	0.523	2.343	0.816	0.002	0.546	75	0.922	-0.078	2.307	0.873	0.003	85	12	38.8
	604812005	0.635	4.004	0.862	0.003	0.415	95	1.053	-0.080	3.843	0.923	0.002	92	13	246.8
	612472597	0.310	3.108	0.729	0.008	0.634	48	1.019	-0.130	3.014	0.806	0.004	75	8	60.1
	638754422	0.519	3.051	0.867	0.001	0.555	76	0.766	-0.053	3.051	0.903	0.001	87	14	75.7
	643185023	0.819	2.258	0.901	0.001	0.177	91	1.376	-0.127	2.160	0.942	0.001	93	11	58.0
	650853796	0.477	2.234	0.820	0.006	0.546	60	0.851	-0.088	2.282	0.867	0.006	74	10	28.8
	658594300	0.873	2.213	0.877	0.009	0.115	77	1.378	-0.160	2.265	0.945	0.007	81	9	47.4
	663835652	0.278	2.754	0.724	0.012	0.719	69	1.090	-0.167	2.610	0.847	0.005	84	7	35.4
	668248235	0.514	2.623	0.846	0.003	0.566	45	0.954	-0.102	2.734	0.893	0.004	59	9	50.1
	682102541	0.683	1.392	0.901	0.037	0.309	7	2.354	-0.884	3.006	0.989	0.022	17	3	19.2
	682449369	0.569	3.280	0.792	0.004	0.494	79	1.027	-0.083	3.145	0.874	0.002	85	12	110.8
	686765762	0.524	2.943	0.877	0.002	0.554	82	0.736	-0.043	2.893	0.918	0.002	87	17	69.6
	706846339	1.128	1.913	0.908	0.033	-0.188	8	2.395	-0.659	2.976	0.988	0.024	15	4	39.2
	737052003	0.449	2.654	0.719	0.010	0.584	49	1.085	-0.141	2.760	0.809	0.006	76	8	49.0
	739574095	0.311	1.679	0.888	0.020	0.722	39	0.721	-0.154	1.811	0.956	0.026	37	5	9.1
	763395383	0.585	3.406	0.882	0.002	0.475	90	0.874	-0.059	3.327	0.925	0.002	92	15	122.6
	763435843	0.549	3.158	0.772	0.006	0.503	67	1.151	-0.132	3.111	0.874	0.004	80	9	85.6
	763456073	0.552	2.506	0.746	0.009	0.469	52	1.130	-0.133	2.633	0.833	0.007	73	9	50.7
	763476303	-0.170	3.065	0.732	0.013	1.023	43	0.243	-0.064	2.881	0.828	0.008	73	4	19.4
	763516763	0.775	3.187	0.889	0.000	0.222	96	1.331	-0.105	2.996	0.929	0.001	97	13	155.8

	763536994	0.522	2.759	0.772	0.007	0.526	59	1.034	-0.122	2.842	0.859	0.005	80	8	55.6
	763557224	-0.054	2.595	0.724	0.010	0.925	54	0.543	-0.125	2.535	0.834	0.007	70	4	16.3
	763597684	0.501	3.075	0.801	0.005	0.569	41	1.006	-0.130	3.291	0.856	0.006	65	8	77.2
	763638144	0.655	3.066	0.857	0.002	0.384	91	1.007	-0.060	2.920	0.892	0.002	92	17	115.3
	763698834	0.199	2.589	0.739	0.006	0.766	55	0.624	-0.084	2.580	0.814	0.007	77	7	24.7
	763719065	0.597	3.222	0.773	0.003	0.449	71	1.150	-0.095	3.024	0.856	0.003	75	12	115.1
	763759525	0.574	2.582	0.836	0.002	0.491	72	0.937	-0.063	2.451	0.888	0.002	78	15	56.7
	763820215	0.595	2.122	0.870	0.002	0.468	75	0.843	-0.052	2.092	0.898	0.004	84	16	36.7
	763840445	0.718	2.771	0.848	0.002	0.318	76	1.234	-0.091	2.643	0.901	0.001	82	14	102.4
	763860675	0.840	2.501	0.838	0.004	0.165	62	1.276	-0.115	2.688	0.886	0.005	79	11	88.4
	763880905	0.087	2.609	0.764	0.008	0.878	44	0.468	-0.093	2.759	0.840	0.007	68	5	21.0
	763901136	0.774	3.086	0.913	0.000	0.268	86	1.022	-0.048	3.063	0.920	0.001	93	21	175.0
	763921366	0.106	2.571	0.759	0.004	0.821	59	0.413	-0.059	2.526	0.825	0.004	72	7	18.5
	763982056	0.445	3.154	0.773	0.007	0.604	61	0.421	-0.005	3.247	0.843	0.007	72	81	107.7
	764002286	0.440	2.368	0.801	0.006	0.530	48	1.062	-0.145	2.619	0.843	0.006	75	7	39.4
	764042746	-0.818	4.969	0.596	0.020	1.419	34	0.468	-0.171	4.093	0.771	0.006	84	3	60.1
	764062976	0.570	3.070	0.733	0.008	0.490	60	1.267	-0.138	3.034	0.845	0.002	82	9	97.0
	764083206	0.609	2.402	0.821	0.004	0.436	46	0.979	-0.120	2.905	0.840	0.007	76	8	53.7
	764143897	0.540	2.976	0.824	0.003	0.527	82	0.997	-0.074	2.761	0.889	0.002	82	13	78.1
	764184357	0.544	3.042	0.807	0.003	0.528	84	1.045	-0.087	2.882	0.873	0.002	92	12	84.2
	764224817	0.495	3.729	0.888	0.001	0.581	99	0.797	-0.057	3.620	0.926	0.001	100	14	138.4
	764245047	0.322	2.061	0.746	0.009	0.683	51	0.717	-0.088	2.106	0.845	0.004	63	8	18.1
	764285508	0.678	3.689	0.927	0.001	0.373	98	1.021	-0.090	3.665	0.954	0.002	100	11	168.8
	764305738	0.613	3.396	0.895	0.000	0.451	98	1.069	-0.085	3.227	0.942	0.001	98	13	129.9
	764325968	0.666	3.809	0.914	0.000	0.379	100	1.142	-0.089	3.633	0.954	0.000	100	13	223.6
	764346198	0.395	3.467	0.744	0.007	0.660	62	0.913	-0.099	3.439	0.831	0.004	77	9	94.6
	764366428	0.523	2.606	0.745	0.011	0.536	50	1.031	-0.122	2.719	0.840	0.006	75	8	49.0
	764447348	0.445	3.229	0.814	0.005	0.629	68	0.756	-0.065	3.153	0.864	0.007	75	12	70.3
	764467579	-0.125	3.393	0.664	0.013	0.989	48	0.771	-0.147	2.944	0.792	0.007	82	5	31.5
	764487809	0.523	3.240	0.828	0.002	0.554	96	1.067	-0.103	3.051	0.912	0.001	98	10	88.3
	764508039	0.557	2.763	0.835	0.005	0.508	62	0.456	-0.002	2.999	0.875	0.006	82	256	159.8
	764588959	0.602	2.943	0.861	0.002	0.466	96	1.010	-0.073	2.785	0.916	0.001	97	14	83.9
	764649650	0.375	2.791	0.691	0.011	0.637	43	1.126	-0.147	2.837	0.814	0.004	76	8	54.8
	764669880	0.304	3.307	0.758	0.012	0.651	33	1.387	-0.239	3.580	0.837	0.005	66	6	102.5
	764710340	0.433	1.975	0.808	0.019	0.603	41	1.136	-0.242	2.245	0.917	0.015	62	5	17.6
	764750800	0.489	2.652	0.781	0.008	0.573	50	0.866	-0.093	2.724	0.855	0.005	63	9	44.4
	764811490	0.573	2.748	0.820	0.003	0.491	91	1.034	-0.079	2.555	0.900	0.001	93	13	64.9
	764831721	0.760	2.553	0.894	0.002	0.283	77	1.261	-0.119	2.574	0.926	0.003	87	11	73.1
	764872181	0.058	3.153	0.722	0.010	0.874	41	0.463	-0.091	3.141	0.822	0.007	64	5	30.9
	764892411	0.563	3.798	0.925	0.000	0.511	100	0.912	-0.065	3.669	0.955	0.000	100	14	175.1
	764953101	0.658	3.287	0.887	0.002	0.394	93	1.147	-0.111	3.207	0.928	0.003	96	10	113.9
	765013792	0.234	2.729	0.748	0.006	0.737	55	0.944	-0.132	2.684	0.838	0.003	79	7	36.6
	765034022	0.607	3.044	0.849	0.001	0.451	95	0.977	-0.063	2.882	0.898	0.002	96	15	97.7
	765074482	0.633	3.498	0.848	0.004	0.412	95	1.082	-0.080	3.298	0.919	0.002	91	13	153.2
	765094712	0.549	3.239	0.892	0.003	0.523	80	0.858	-0.063	3.163	0.930	0.002	82	14	93.8
	765155402	0.515	3.418	0.789	0.006	0.554	76	1.079	-0.115	3.326	0.886	0.002	89	9	106.3
	765195863	-0.058	3.635	0.667	0.013	0.958	47	0.787	-0.143	3.476	0.797	0.005	76	6	56.4
	765216093	0.511	3.638	0.795	0.004	0.565	78	1.065	-0.109	3.486	0.883	0.002	85	10	128.0
	765256553	0.472	2.820	0.784	0.006	0.561	52	1.131	-0.164	3.049	0.863	0.005	74	7	60.3
	765276783	0.453	3.012	0.782	0.006	0.622	61	0.909	-0.098	3.005	0.872	0.004	77	9	61.6
	765317243	0.074	2.593	0.746	0.009	0.872	64	0.453	-0.078	2.535	0.844	0.006	68	6	17.8
	765337473	0.452	2.280	0.744	0.007	0.544	50	1.072	-0.128	2.482	0.837	0.003	74	8	40.1
	765377934	0.561	3.437	0.908	0.002	0.515	95	1.011	-0.114	3.387	0.953	0.001	95	9	97.4
	765560005	0.482	3.074	0.794	0.006	0.579	73	0.988	-0.110	3.039	0.885	0.003	85	9	67.8
	809635352	0.393	3.243	0.763	0.007	0.673	56	0.585	-0.046	3.357	0.823	0.005	81	13	70.5

	863126187	0.720	2.838	0.803	0.006	0.305	54	1.008	-0.093	3.042	0.879	0.004	70	11	84.9
	892969023	0.055	3.306	0.766	0.004	0.882	46	0.364	-0.056	3.227	0.828	0.005	74	6	34.5
	937495960	0.550	3.310	0.814	0.005	0.526	84	0.939	-0.089	3.237	0.904	0.003	86	11	91.2
	953045535	0.449	2.545	0.774	0.009	0.548	34	0.910	-0.116	2.894	0.820	0.007	65	8	47.4
	970836795	0.303	2.437	0.742	0.011	0.663	38	1.116	-0.169	2.702	0.826	0.004	87	7	40.3
	**Mean**	0.568	2.818	0.825	0.006	0.455	70	1.104	-0.121	2.789	0.891	0.004	81	12	81.5
	**Std. Err.**	0.139	0.173	0.051	0.003	0.138	9	0.199	0.039	0.135	0.030	0.001	5	2	5.0

Table S2. The intra-individual (within-body) *beta*-DAR modeling for the HMP dataset (Full version, Demo version is in Table 2)

Order	Subject Number	Power Law (PL)						PL with Exponential Cutoff (PLEC)							
		z	ln(c)	R	p-value	g	N^*	z	d	ln(c)	R	p-value	N^*	A_{max}	D_{max}
$q=0$	132902142	0.731	0.168	0.987	0.000	0.338	100	1.060	-0.066	0.012	0.993	0.000	100	16	6.6
	147406386	0.721	0.184	0.994	0.000	0.351	100	0.908	-0.028	0.051	0.997	0.000	100	32	9.9
	158013734	0.714	0.227	0.992	0.000	0.360	100	0.940	-0.031	0.051	0.997	0.000	100	30	10.1
	158114885	0.681	0.211	0.991	0.000	0.396	100	0.886	-0.027	0.044	0.996	0.000	100	33	9.4
	158155345	0.684	0.203	0.993	0.000	0.393	100	0.898	-0.033	0.052	0.997	0.000	100	28	8.5
	158216035	0.674	0.187	0.992	0.000	0.403	100	0.876	-0.032	0.053	0.997	0.000	100	27	7.9
	158236265	0.708	0.219	0.991	0.000	0.366	100	0.915	-0.030	0.065	0.996	0.000	100	30	9.8
	158276726	0.723	0.214	0.994	0.000	0.349	100	0.913	-0.025	0.059	0.998	0.000	100	36	11.3
	158398106	0.706	0.203	0.993	0.000	0.368	100	0.910	-0.031	0.059	0.997	0.000	100	29	9.3
	158418336	0.733	0.154	0.993	0.000	0.337	100	0.953	-0.035	0.007	0.997	0.000	100	27	9.0
	158438567	0.698	0.235	0.989	0.000	0.377	100	0.953	-0.039	0.055	0.996	0.000	100	25	8.6
	158458797	0.716	0.221	0.991	0.000	0.357	100	0.957	-0.033	0.033	0.997	0.000	100	29	9.8
	158479027	0.710	0.235	0.989	0.000	0.364	100	0.983	-0.043	0.053	0.996	0.000	100	23	8.5
	158499257	0.736	0.192	0.992	0.000	0.334	100	1.002	-0.047	0.038	0.997	0.000	100	21	8.1
	158721788	0.730	0.176	0.992	0.000	0.340	100	0.960	-0.039	0.033	0.996	0.000	100	25	8.6
	158742018	0.720	0.225	0.992	0.000	0.352	100	0.930	-0.028	0.055	0.997	0.000	100	33	10.9
	158802708	0.718	0.195	0.993	0.000	0.354	100	0.936	-0.033	0.042	0.997	0.000	100	28	9.4
	158822939	0.639	0.218	0.990	0.000	0.441	100	0.870	-0.037	0.065	0.996	0.000	100	24	7.0
	158883629	0.694	0.244	0.991	0.000	0.382	100	0.912	-0.030	0.073	0.996	0.000	100	30	9.7
	158924089	0.670	0.253	0.985	0.000	0.408	100	0.959	-0.044	0.049	0.994	0.000	100	22	7.8
	158944319	0.731	0.215	0.992	0.000	0.339	100	0.955	-0.032	0.049	0.997	0.000	100	30	10.3
	158964549	0.733	0.170	0.993	0.000	0.337	100	0.941	-0.031	0.023	0.997	0.000	100	30	9.8
	158984779	0.778	0.158	0.992	0.000	0.283	100	1.034	-0.043	-0.001	0.997	0.000	100	24	9.5
	159005010	0.681	0.268	0.986	0.000	0.395	100	0.931	-0.033	0.064	0.994	0.000	100	28	9.4
	159025240	0.704	0.203	0.992	0.000	0.370	100	0.898	-0.026	0.044	0.997	0.000	100	35	10.3
	159085930	0.716	0.246	0.990	0.000	0.356	100	0.953	-0.031	0.053	0.996	0.000	100	30	10.5
	159146620	0.664	0.227	0.990	0.000	0.415	100	0.891	-0.034	0.066	0.996	0.000	100	26	8.0
	159166850	0.660	0.224	0.991	0.000	0.419	100	0.891	-0.035	0.061	0.997	0.000	100	26	7.8
	159227541	0.704	0.229	0.993	0.000	0.371	100	0.910	-0.027	0.061	0.997	0.000	100	33	10.4
	159247771	0.681	0.211	0.991	0.000	0.396	100	0.886	-0.027	0.044	0.996	0.000	100	33	9.4
	159389382	0.775	0.150	0.995	0.000	0.288	100	0.976	-0.030	0.008	0.998	0.000	100	32	11.2
	159470302	0.663	0.265	0.988	0.000	0.416	100	0.897	-0.031	0.074	0.994	0.000	100	29	9.0
	159713063	0.665	0.254	0.991	0.000	0.413	100	0.882	-0.029	0.077	0.996	0.000	100	31	9.2
	159733294	0.660	0.280	0.988	0.000	0.418	100	0.902	-0.032	0.083	0.995	0.000	100	28	8.9
	160744799	0.740	0.202	0.992	0.000	0.328	100	0.956	-0.031	0.042	0.997	0.000	100	31	10.6
	160825720	0.766	0.171	0.995	0.000	0.298	100	0.976	-0.033	0.031	0.998	0.000	100	29	10.5
	160866180	0.809	0.170	0.996	0.000	0.247	100	0.990	-0.025	0.029	0.998	0.000	100	40	14.6
	160886410	0.748	0.171	0.995	0.000	0.320	100	0.929	-0.029	0.051	0.997	0.000	100	32	10.5
	160906640	0.750	0.155	0.995	0.000	0.318	100	0.942	-0.032	0.036	0.998	0.000	100	29	9.7
	160947100	0.731	0.215	0.992	0.000	0.339	100	0.955	-0.032	0.049	0.997	0.000	100	30	10.3
	160967330	0.741	0.180	0.995	0.000	0.328	100	0.947	-0.033	0.043	0.998	0.000	100	29	9.8
	160987560	0.760	0.161	0.995	0.000	0.306	100	0.966	-0.033	0.024	0.998	0.000	100	29	10.2
	161007791	0.746	0.177	0.992	0.000	0.322	100	0.998	-0.038	-0.001	0.997	0.000	100	26	9.6
	161270782	0.796	0.163	0.995	0.000	0.264	100	1.011	-0.034	0.020	0.998	0.000	100	30	11.4
	161311242	0.759	0.158	0.995	0.000	0.307	100	0.970	-0.037	0.037	0.998	0.000	100	26	9.3
	161412393	0.722	0.131	0.993	0.000	0.349	100	1.028	-0.071	0.023	0.998	0.000	100	14	5.7
	161473083	0.797	0.185	0.996	0.000	0.262	100	0.983	-0.025	0.034	0.999	0.000	100	40	14.5
	161554003	0.723	0.179	0.994	0.000	0.349	100	0.940	-0.036	0.044	0.997	0.000	100	26	8.7
	178713055	0.742	0.233	0.988	0.000	0.327	100	1.031	-0.046	0.042	0.995	0.000	100	22	9.2

	184349034	0.848	0.060	0.995	0.000	0.198	100	1.122	-0.064	-0.037	0.998	0.000	100	18	7.9
	208027353	0.717	0.201	0.992	0.000	0.355	100	0.945	-0.031	0.024	0.997	0.000	100	30	9.9
	246515023	0.751	0.211	0.995	0.000	0.317	100	0.935	-0.024	0.061	0.998	0.000	100	38	12.6
	256789458	0.816	0.144	0.995	0.000	0.239	100	1.065	-0.050	0.026	0.998	0.000	100	21	9.2
	275382046	0.848	0.087	0.993	0.000	0.198	100	1.255	-0.103	-0.028	0.997	0.000	100	12	6.4
	289996019	0.721	0.198	0.990	0.000	0.351	100	0.988	-0.047	0.044	0.996	0.000	100	21	7.8
	295137534	0.802	0.148	0.996	0.000	0.256	100	0.993	-0.032	0.029	0.999	0.000	100	31	11.5
	336497421	0.738	0.185	0.993	0.000	0.331	100	0.970	-0.039	0.041	0.997	0.000	100	25	8.9
	368533040	0.737	0.197	0.995	0.000	0.333	100	0.941	-0.031	0.053	0.998	0.000	100	30	10.2
	370027359	0.723	0.203	0.991	0.000	0.349	100	0.975	-0.038	0.025	0.997	0.000	100	26	9.1
	370425937	0.716	0.217	0.993	0.000	0.357	100	0.911	-0.026	0.057	0.997	0.000	100	35	10.9
	375450439	0.704	0.247	0.992	0.000	0.370	100	0.919	-0.028	0.072	0.997	0.000	100	32	10.4
	404239096	0.740	0.171	0.993	0.000	0.329	100	0.983	-0.041	0.020	0.997	0.000	100	24	8.7
	414519462	0.734	0.202	0.994	0.000	0.336	100	0.936	-0.027	0.037	0.998	0.000	100	35	11.3
	451588811	0.775	0.168	0.993	0.000	0.288	100	0.998	-0.035	0.020	0.997	0.000	100	28	10.5
	465578759	0.756	0.204	0.994	0.000	0.311	100	0.951	-0.026	0.045	0.997	0.000	100	37	12.4
	492786515	0.741	0.168	0.995	0.000	0.328	100	0.919	-0.027	0.042	0.997	0.000	100	34	10.6
	508703490	0.743	0.211	0.991	0.000	0.326	100	0.959	-0.033	0.059	0.996	0.000	100	29	10.4
	516889361	0.887	0.070	0.998	0.000	0.150	100	1.110	-0.056	0.007	0.999	0.000	100	20	9.1
	517810313	0.675	0.219	0.992	0.000	0.402	100	0.862	-0.028	0.088	0.996	0.000	100	30	8.8
	550534656	0.696	0.150	0.995	0.005	0.376	100	1.049	-0.117	0.144	1.000	0.027	73	9	4.1
	553359145	0.730	0.214	0.993	0.000	0.341	100	0.930	-0.028	0.058	0.997	0.000	100	34	11.0
	604812005	0.795	0.144	0.996	0.000	0.265	100	0.981	-0.030	0.021	0.998	0.000	100	33	11.9
	612472597	0.741	0.209	0.991	0.000	0.327	100	0.987	-0.033	0.008	0.997	0.000	100	30	10.9
	638754422	0.777	0.181	0.996	0.000	0.286	100	0.962	-0.027	0.043	0.999	0.000	100	36	12.5
	643185023	0.799	0.182	0.993	0.000	0.260	100	1.063	-0.047	0.030	0.997	0.000	100	23	9.8
	650853796	0.793	0.146	0.996	0.000	0.267	100	0.975	-0.029	0.025	0.998	0.000	100	34	11.9
	658594300	0.802	0.152	0.989	0.000	0.255	100	1.156	-0.082	0.028	0.995	0.000	100	14	6.9
	663835652	0.736	0.186	0.991	0.000	0.333	100	0.985	-0.040	0.021	0.996	0.000	100	25	9.0
	668248235	0.710	0.193	0.993	0.000	0.363	100	0.920	-0.032	0.046	0.998	0.000	100	29	9.2
	682102541	0.825	0.037	0.990	0.010	0.225	100	1.278	-0.150	0.060	0.999	0.031	58	9	4.6
	682449369	0.664	0.271	0.989	0.000	0.414	100	0.885	-0.030	0.099	0.995	0.000	100	29	9.0
	686765762	0.726	0.203	0.995	0.000	0.345	100	0.910	-0.028	0.073	0.998	0.000	100	33	10.3
	706846339	0.864	0.091	0.993	0.007	0.179	100	1.533	-0.203	0.007	0.999	0.033	55	8	4.8
	737052003	0.726	0.200	0.990	0.000	0.345	100	0.971	-0.035	0.018	0.996	0.000	100	27	9.6
	739574095	0.824	0.048	0.994	0.001	0.227	100	1.293	-0.130	-0.048	0.999	0.003	100	10	5.1
	763395383	0.698	0.193	0.991	0.000	0.377	100	0.938	-0.038	0.034	0.996	0.000	100	25	8.1
	763435843	0.696	0.228	0.988	0.000	0.378	100	0.980	-0.045	0.039	0.995	0.000	100	22	8.0
	763456073	0.678	0.238	0.989	0.000	0.399	100	0.910	-0.034	0.065	0.995	0.000	100	27	8.6
	763476303	0.755	0.173	0.994	0.000	0.311	100	0.974	-0.035	0.028	0.998	0.000	100	28	10.0
	763516763	0.746	0.193	0.993	0.000	0.322	100	0.969	-0.034	0.036	0.997	0.000	100	29	10.2
	763536994	0.702	0.200	0.987	0.000	0.372	100	0.961	-0.041	0.028	0.994	0.000	100	23	8.1
	763557224	0.750	0.182	0.991	0.000	0.317	100	0.993	-0.039	0.020	0.996	0.000	100	26	9.5
	763597684	0.666	0.199	0.990	0.000	0.412	100	0.890	-0.036	0.050	0.996	0.000	100	25	7.6
	763638144	0.765	0.201	0.994	0.000	0.301	100	0.954	-0.026	0.053	0.997	0.000	100	36	12.5
	763698834	0.698	0.230	0.991	0.000	0.377	100	0.922	-0.031	0.054	0.996	0.000	100	30	9.6
	763719065	0.726	0.229	0.990	0.000	0.344	100	0.969	-0.032	0.032	0.996	0.000	100	30	10.6
	763759525	0.773	0.182	0.995	0.000	0.290	100	0.942	-0.022	0.044	0.998	0.000	100	42	13.8
	763820215	0.710	0.230	0.992	0.000	0.363	100	0.915	-0.030	0.078	0.996	0.000	100	31	10.0
	763840445	0.712	0.242	0.988	0.000	0.360	100	0.962	-0.033	0.038	0.995	0.000	100	29	10.2
	763860675	0.710	0.230	0.992	0.000	0.363	100	0.915	-0.030	0.078	0.996	0.000	100	31	10.0
	763880905	0.698	0.173	0.993	0.000	0.377	100	0.922	-0.038	0.033	0.998	0.000	100	25	7.9
	763901136	0.763	0.190	0.995	0.000	0.303	100	0.941	-0.024	0.045	0.998	0.000	100	40	13.1
	763921366	0.724	0.206	0.991	0.000	0.347	100	0.949	-0.030	0.023	0.997	0.000	100	32	10.5
	763982056	0.704	0.212	0.991	0.000	0.370	100	0.940	-0.036	0.046	0.996	0.000	100	26	8.8

	764002286	0.725	0.197	0.993	0.000	0.347	100	0.932	-0.031	0.050	0.997	0.000	100	30	9.7
	764042746	0.756	0.205	0.993	0.000	0.310	100	0.982	-0.033	0.037	0.998	0.000	100	30	11.0
	764062976	0.750	0.221	0.993	0.000	0.318	100	0.971	-0.031	0.049	0.997	0.000	100	32	11.4
	764083206	0.693	0.197	0.991	0.000	0.382	100	0.915	-0.034	0.041	0.996	0.000	100	27	8.6
	764143897	0.675	0.266	0.987	0.000	0.403	100	0.920	-0.033	0.066	0.995	0.000	100	28	9.2
	764184357	0.693	0.197	0.991	0.000	0.382	100	0.915	-0.034	0.041	0.996	0.000	100	27	8.6
	764224817	0.723	0.225	0.992	0.000	0.348	100	0.969	-0.037	0.052	0.997	0.000	100	26	9.4
	764245047	0.729	0.212	0.992	0.000	0.342	100	0.940	-0.032	0.063	0.996	0.000	100	29	10.0
	764285508	0.619	0.200	0.984	0.000	0.463	100	0.973	-0.071	0.032	0.994	0.000	100	14	5.0
	764305738	0.685	0.195	0.992	0.000	0.391	100	0.894	-0.032	0.047	0.997	0.000	100	28	8.5
	764325968	0.743	0.187	0.992	0.000	0.325	100	0.967	-0.034	0.029	0.997	0.000	100	29	10.0
	764346198	0.744	0.208	0.993	0.000	0.324	100	0.969	-0.031	0.033	0.997	0.000	100	31	11.0
	764366428	0.727	0.215	0.992	0.000	0.344	100	0.957	-0.035	0.052	0.996	0.000	100	27	9.6
	764447348	0.843	0.118	0.996	0.000	0.205	100	1.019	-0.028	0.001	0.998	0.000	100	36	14.1
	764467579	0.744	0.210	0.994	0.000	0.324	100	0.944	-0.029	0.062	0.997	0.000	100	33	11.1
	764487809	0.736	0.188	0.993	0.000	0.334	100	0.944	-0.032	0.041	0.997	0.000	100	30	10.0
	764508039	0.750	0.189	0.994	0.000	0.318	100	0.984	-0.037	0.033	0.998	0.000	100	26	9.7
	764588959	0.703	0.255	0.989	0.000	0.371	100	0.946	-0.035	0.075	0.995	0.000	100	27	9.4
	764649650	0.751	0.195	0.994	0.000	0.316	100	0.938	-0.025	0.043	0.997	0.000	100	38	12.3
	764669880	0.698	0.221	0.991	0.000	0.376	100	0.896	-0.030	0.082	0.996	0.000	100	30	9.3
	764710340	0.875	0.026	0.993	0.000	0.162	100	1.164	-0.067	-0.076	0.997	0.000	100	17	8.0
	764750800	0.698	0.196	0.991	0.000	0.377	100	0.930	-0.037	0.042	0.996	0.000	100	25	8.3
	764811490	0.730	0.219	0.993	0.000	0.340	100	0.929	-0.027	0.064	0.997	0.000	100	34	11.1
	764831721	0.735	0.166	0.992	0.000	0.334	100	0.980	-0.041	0.013	0.996	0.000	100	24	8.5
	764872181	0.689	0.200	0.990	0.000	0.387	100	0.932	-0.039	0.038	0.996	0.000	100	24	7.9
	764892411	0.712	0.195	0.992	0.000	0.361	100	0.937	-0.034	0.036	0.996	0.000	100	27	9.1
	764953101	0.730	0.165	0.992	0.000	0.341	100	0.962	-0.041	0.031	0.996	0.000	100	23	8.2
	765013792	0.712	0.225	0.990	0.000	0.361	100	0.950	-0.033	0.039	0.997	0.000	100	29	9.8
	765034022	0.714	0.219	0.991	0.000	0.359	100	0.940	-0.031	0.043	0.996	0.000	100	30	10.0
	765074482	0.771	0.150	0.994	0.000	0.293	100	0.971	-0.030	0.009	0.997	0.000	100	32	11.1
	765094712	0.720	0.189	0.992	0.000	0.351	100	0.962	-0.038	0.028	0.997	0.000	100	25	8.7
	765155402	0.746	0.163	0.994	0.000	0.322	100	0.942	-0.030	0.025	0.998	0.000	100	32	10.4
	765195863	0.746	0.201	0.995	0.000	0.323	100	0.918	-0.023	0.060	0.998	0.000	100	40	12.6
	765216093	0.710	0.226	0.992	0.000	0.364	100	0.934	-0.034	0.068	0.997	0.000	100	27	9.3
	765256553	0.710	0.205	0.989	0.000	0.363	100	0.950	-0.038	0.045	0.995	0.000	100	25	8.6
	765276783	0.728	0.181	0.992	0.000	0.342	100	0.957	-0.035	0.019	0.997	0.000	100	28	9.4
	765317243	0.737	0.164	0.993	0.000	0.331	100	0.931	-0.031	0.035	0.996	0.000	100	30	9.7
	765337473	0.727	0.217	0.994	0.000	0.344	100	0.898	-0.023	0.078	0.997	0.000	100	40	12.0
	765377934	0.699	0.142	0.993	0.000	0.376	100	0.966	-0.054	0.016	0.998	0.000	100	18	6.3
	765560005	0.690	0.186	0.994	0.000	0.386	100	0.895	-0.033	0.050	0.997	0.000	100	27	8.3
	809635352	0.730	0.227	0.993	0.000	0.341	100	0.951	-0.029	0.046	0.998	0.000	100	32	11.1
	863126187	0.711	0.181	0.995	0.000	0.362	100	0.906	-0.033	0.060	0.998	0.000	100	28	8.7
	892969023	0.762	0.174	0.994	0.000	0.303	100	0.966	-0.029	0.022	0.998	0.000	100	33	11.3
	937495960	0.813	0.100	0.995	0.000	0.241	100	1.021	-0.037	-0.020	0.997	0.000	100	28	10.5
	953045535	0.769	0.185	0.996	0.000	0.295	100	0.947	-0.026	0.053	0.998	0.000	100	37	12.5
	970836795	0.734	0.215	0.994	0.000	0.336	100	0.931	-0.027	0.061	0.998	0.000	100	34	11.2
	**Mean**	0.733	0.192	0.992	0.000	0.337	100	0.964	-0.038	0.041	0.997	0.001	99	28	9.6
	**Std. Err.**	0.004	0.003	0.000	0.000	0.004	0	0.007	0.002	0.002	0.000	0.000	0	1	0.1
q=1	132902142	0.617	0.239	0.956	0.000	0.461	100	1.136	-0.104	-0.007	0.978	0.000	100	11	4.8
	147406386	0.544	0.314	0.970	0.000	0.540	100	0.886	-0.052	0.073	0.987	0.000	100	17	5.5
	158013734	0.594	0.319	0.972	0.000	0.488	100	0.939	-0.048	0.050	0.988	0.000	100	20	6.8
	158114885	0.528	0.341	0.961	0.000	0.556	100	0.891	-0.048	0.045	0.985	0.000	100	18	5.8
	158155345	0.546	0.310	0.967	0.000	0.537	100	0.897	-0.053	0.062	0.986	0.000	100	17	5.5
	158216035	0.550	0.281	0.971	0.000	0.533	100	0.894	-0.055	0.052	0.988	0.000	100	16	5.2
	158236265	0.540	0.365	0.958	0.000	0.543	100	0.931	-0.057	0.074	0.982	0.000	100	16	5.8

	158276726	0.614	0.319	0.978	0.000	0.468	100	0.928	-0.042	0.063	0.991	0.000	100	22	7.5
	158398106	0.520	0.351	0.958	0.000	0.564	100	0.876	-0.054	0.100	0.979	0.000	100	16	5.3
	158418336	0.589	0.241	0.965	0.000	0.493	100	0.983	-0.063	-0.021	0.984	0.000	100	16	5.5
	158438567	0.640	0.294	0.976	0.000	0.440	100	1.021	-0.058	0.025	0.991	0.000	100	18	6.9
	158458797	0.561	0.345	0.960	0.000	0.522	100	0.951	-0.054	0.041	0.983	0.000	100	18	6.2
	158479027	0.609	0.325	0.963	0.000	0.472	100	1.050	-0.070	0.032	0.986	0.000	100	15	6.2
	158499257	0.653	0.269	0.971	0.000	0.425	100	1.098	-0.079	0.014	0.989	0.000	100	14	6.1
	158721788	0.564	0.281	0.956	0.000	0.517	100	0.965	-0.067	0.032	0.978	0.000	100	14	5.1
	158742018	0.624	0.318	0.970	0.000	0.457	100	0.993	-0.049	0.017	0.987	0.000	100	20	7.5
	158802708	0.585	0.294	0.972	0.000	0.498	100	0.935	-0.053	0.048	0.988	0.000	100	18	6.0
	158822939	0.502	0.305	0.964	0.000	0.581	100	0.834	-0.053	0.084	0.984	0.000	100	16	4.7
	158883629	0.573	0.380	0.960	0.000	0.510	100	0.980	-0.056	0.063	0.984	0.000	100	17	6.6
	158924089	0.561	0.367	0.952	0.000	0.523	100	1.026	-0.071	0.039	0.980	0.000	100	15	5.8
	158944319	0.523	0.391	0.946	0.000	0.560	100	0.962	-0.063	0.065	0.978	0.000	100	15	5.6
	158964549	0.736	0.159	0.985	0.000	0.332	100	1.058	-0.049	-0.068	0.994	0.000	100	22	8.4
	158984779	0.625	0.299	0.956	0.000	0.452	100	1.134	-0.085	-0.017	0.981	0.000	100	13	5.9
	159005010	0.565	0.382	0.950	0.000	0.518	100	0.977	-0.055	0.045	0.979	0.000	100	18	6.6
	159025240	0.527	0.323	0.943	0.000	0.556	100	0.911	-0.051	0.009	0.973	0.000	100	18	5.6
	159085930	0.566	0.406	0.950	0.000	0.517	100	0.996	-0.057	0.056	0.979	0.000	100	17	6.7
	159146620	0.491	0.349	0.951	0.000	0.592	100	0.869	-0.057	0.082	0.978	0.000	100	15	4.8
	159166850	0.486	0.340	0.950	0.000	0.596	100	0.862	-0.057	0.075	0.978	0.000	100	15	4.7
	159227541	0.596	0.316	0.969	0.000	0.486	100	0.945	-0.046	0.031	0.987	0.000	100	20	6.9
	159247771	0.528	0.341	0.961	0.000	0.556	100	0.891	-0.048	0.045	0.985	0.000	100	18	5.8
	159389382	0.697	0.208	0.983	0.000	0.377	100	1.017	-0.048	-0.018	0.992	0.000	100	21	7.8
	159470302	0.578	0.363	0.957	0.000	0.505	100	0.982	-0.054	0.033	0.982	0.000	100	18	6.7
	159713063	0.529	0.376	0.964	0.000	0.555	100	0.872	-0.045	0.097	0.986	0.000	100	19	6.1
	159733294	0.530	0.384	0.958	0.000	0.553	100	0.894	-0.048	0.087	0.982	0.000	100	19	6.1
	160744799	0.593	0.321	0.963	0.000	0.488	100	0.963	-0.053	0.046	0.983	0.000	100	18	6.5
	160825720	0.655	0.249	0.982	0.000	0.424	100	0.969	-0.050	0.040	0.992	0.000	100	19	7.0
	160866180	0.672	0.317	0.972	0.000	0.404	100	1.032	-0.050	0.036	0.987	0.000	100	21	8.4
	160886410	0.634	0.261	0.978	0.000	0.446	100	0.957	-0.051	0.046	0.990	0.000	100	19	6.6
	160906640	0.578	0.265	0.972	0.000	0.505	100	0.930	-0.059	0.046	0.987	0.000	100	16	5.4
	160947100	0.523	0.391	0.946	0.000	0.560	100	0.962	-0.063	0.065	0.978	0.000	100	15	5.6
	160967330	0.636	0.251	0.984	0.000	0.445	100	0.927	-0.046	0.057	0.993	0.000	100	20	6.7
	160987560	0.615	0.268	0.980	0.000	0.467	100	0.972	-0.057	0.030	0.993	0.000	100	17	6.2
	161007791	0.657	0.234	0.971	0.000	0.420	100	1.076	-0.064	-0.061	0.989	0.000	100	17	6.7
	161270782	0.690	0.250	0.982	0.000	0.385	100	1.052	-0.058	0.009	0.994	0.000	100	18	7.5
	161311242	0.621	0.245	0.980	0.000	0.460	100	0.964	-0.061	0.048	0.992	0.000	100	16	5.7
	161412393	0.409	0.306	0.873	0.013	0.666	94	1.221	-0.187	0.009	0.952	0.012	91	7	2.9
	161473083	0.674	0.315	0.982	0.000	0.404	100	0.999	-0.043	0.050	0.994	0.000	100	23	8.9
	161554003	0.528	0.322	0.961	0.000	0.556	100	0.934	-0.068	0.069	0.984	0.000	100	14	4.9
	178713055	0.647	0.333	0.967	0.000	0.433	100	1.069	-0.067	0.053	0.985	0.000	100	16	7.0
	184349034	0.701	0.150	0.972	0.000	0.368	100	1.268	-0.132	-0.049	0.988	0.001	100	10	4.7
	208027353	0.598	0.298	0.970	0.000	0.484	100	0.965	-0.051	0.011	0.988	0.000	100	19	6.6
	246515023	0.576	0.397	0.959	0.000	0.507	100	0.962	-0.051	0.082	0.983	0.000	100	19	7.0
	256789458	0.689	0.259	0.970	0.000	0.385	100	1.199	-0.102	0.017	0.985	0.000	100	12	5.9
	275382046	0.742	0.161	0.970	0.001	0.320	100	1.482	-0.187	-0.048	0.989	0.004	100	8	4.6
	289996019	0.708	0.216	0.981	0.000	0.364	100	1.090	-0.068	-0.004	0.993	0.000	100	16	6.9
	295137534	0.717	0.231	0.981	0.000	0.354	100	1.101	-0.064	-0.007	0.992	0.000	100	17	7.5
	336497421	0.627	0.277	0.973	0.000	0.453	100	1.039	-0.069	0.021	0.990	0.000	100	15	6.0
	368533040	0.530	0.376	0.952	0.000	0.553	100	0.956	-0.064	0.075	0.981	0.000	100	15	5.5
	370027359	0.546	0.340	0.952	0.000	0.537	100	0.974	-0.065	0.038	0.979	0.000	100	15	5.5
	370425937	0.558	0.356	0.965	0.000	0.525	100	0.910	-0.047	0.069	0.986	0.000	100	20	6.4
	375450439	0.507	0.423	0.949	0.000	0.577	100	0.894	-0.051	0.108	0.980	0.000	100	17	5.9
	404239096	0.599	0.285	0.967	0.000	0.483	100	1.032	-0.073	0.016	0.987	0.000	100	14	5.6

	414519462	0.556	0.365	0.966	0.000	0.528	100	0.938	-0.051	0.054	0.988	0.000	100	19	6.4
	451588811	0.616	0.314	0.959	0.000	0.464	100	1.076	-0.073	0.008	0.981	0.000	100	15	6.2
	465578759	0.574	0.397	0.961	0.000	0.509	100	0.980	-0.054	0.066	0.985	0.000	100	18	6.9
	492786515	0.553	0.291	0.967	0.000	0.530	100	0.878	-0.049	0.062	0.984	0.000	100	18	5.6
	508703490	0.602	0.344	0.964	0.000	0.480	100	0.996	-0.060	0.067	0.984	0.000	100	17	6.5
	516889361	0.912	0.046	0.997	0.000	0.118	100	1.184	-0.069	-0.030	0.999	0.000	100	17	8.6
	517810313	0.451	0.389	0.943	0.000	0.630	100	0.803	-0.053	0.141	0.973	0.000	100	15	4.6
	550534656	0.442	0.250	0.975	0.025	0.626	33	2.564	-0.608	-0.145	0.999	0.035	12	4	2.7
	553359145	0.556	0.377	0.965	0.000	0.528	100	0.927	-0.051	0.087	0.986	0.000	100	18	6.3
	604812005	0.680	0.239	0.983	0.000	0.397	100	1.010	-0.053	0.020	0.994	0.000	100	19	7.3
	612472597	0.713	0.216	0.969	0.000	0.356	100	1.088	-0.050	-0.090	0.983	0.000	100	22	8.8
	638754422	0.618	0.326	0.973	0.000	0.464	100	0.965	-0.050	0.068	0.987	0.000	100	19	7.1
	643185023	0.703	0.274	0.974	0.000	0.369	100	1.133	-0.076	0.027	0.987	0.000	100	15	7.0
	650853796	0.728	0.196	0.983	0.000	0.341	100	1.086	-0.057	-0.041	0.993	0.000	100	19	8.0
	658594300	0.707	0.237	0.964	0.001	0.365	100	1.280	-0.133	0.035	0.983	0.001	100	10	5.2
	663835652	0.571	0.319	0.955	0.000	0.511	100	1.008	-0.070	0.028	0.980	0.000	100	14	5.6
	668248235	0.507	0.341	0.959	0.000	0.576	100	0.874	-0.055	0.082	0.984	0.000	100	16	5.0
	682102541	0.558	0.257	0.964	0.036	0.513	43	2.591	-0.602	-0.042	1.000	0.018	9	4	3.2
	682449369	0.502	0.410	0.951	0.000	0.582	100	0.872	-0.051	0.121	0.979	0.000	100	17	5.6
	686765762	0.578	0.332	0.974	0.000	0.506	100	0.920	-0.052	0.090	0.989	0.000	100	18	6.1
	706846339	0.849	0.091	0.991	0.009	0.192	96	1.668	-0.256	0.013	0.999	0.030	74	7	4.4
	737052003	0.640	0.255	0.964	0.000	0.438	100	1.019	-0.055	-0.027	0.982	0.000	100	19	6.9
	739574095	0.744	0.031	0.944	0.017	0.307	100	2.155	-0.377	-0.333	0.990	0.019	74	6	3.6
	763395383	0.546	0.306	0.958	0.000	0.538	100	0.956	-0.065	0.033	0.982	0.000	100	15	5.2
	763435843	0.567	0.352	0.947	0.000	0.514	100	1.085	-0.082	0.008	0.981	0.000	100	13	5.6
	763456073	0.510	0.373	0.949	0.000	0.573	100	0.918	-0.059	0.070	0.978	0.000	100	16	5.3
	763476303	0.595	0.317	0.965	0.000	0.488	100	1.035	-0.070	0.024	0.986	0.000	100	15	5.9
	763516763	0.611	0.301	0.968	0.000	0.470	100	0.987	-0.057	0.036	0.986	0.000	100	17	6.4
	763536994	0.544	0.327	0.947	0.000	0.538	100	0.985	-0.070	0.034	0.975	0.000	100	14	5.2
	763557224	0.567	0.348	0.940	0.000	0.514	100	1.063	-0.079	0.018	0.972	0.000	100	13	5.6
	763597684	0.493	0.319	0.950	0.000	0.589	100	0.865	-0.059	0.073	0.977	0.000	100	15	4.6
	763638144	0.672	0.284	0.984	0.000	0.406	100	0.966	-0.041	0.055	0.993	0.000	100	24	8.6
	763698834	0.533	0.356	0.961	0.000	0.550	100	0.899	-0.051	0.071	0.984	0.000	100	18	5.8
	763719065	0.634	0.308	0.971	0.000	0.446	100	0.996	-0.048	0.013	0.988	0.000	100	21	7.7
	763759525	0.640	0.311	0.981	0.000	0.439	100	0.943	-0.040	0.064	0.992	0.000	100	23	8.2
	763820215	0.559	0.390	0.955	0.000	0.524	100	0.980	-0.061	0.077	0.981	0.000	100	16	6.2
	763840445	0.585	0.366	0.960	0.000	0.497	100	1.013	-0.057	0.018	0.985	0.000	100	18	6.9
	763860675	0.559	0.390	0.955	0.000	0.524	100	0.980	-0.061	0.077	0.981	0.000	100	16	6.2
	763880905	0.543	0.282	0.959	0.000	0.539	100	0.970	-0.072	0.016	0.983	0.000	100	14	4.8
	763901136	0.705	0.238	0.988	0.000	0.368	100	0.962	-0.034	0.029	0.995	0.000	100	28	9.8
	763921366	0.571	0.336	0.959	0.000	0.512	100	0.982	-0.055	0.001	0.985	0.000	100	18	6.4
	763982056	0.508	0.386	0.932	0.000	0.574	100	0.979	-0.071	0.054	0.973	0.000	100	14	5.1
	764002286	0.581	0.313	0.969	0.000	0.501	100	0.952	-0.056	0.052	0.987	0.000	100	17	6.0
	764042746	0.719	0.254	0.982	0.000	0.352	100	1.102	-0.055	-0.031	0.994	0.000	100	20	8.7
	764062976	0.659	0.310	0.978	0.000	0.419	100	1.012	-0.049	0.035	0.991	0.000	100	21	8.1
	764083206	0.512	0.327	0.960	0.000	0.572	100	0.892	-0.058	0.059	0.984	0.000	100	15	5.0
	764143897	0.490	0.437	0.934	0.000	0.593	100	0.923	-0.057	0.084	0.974	0.000	100	16	5.6
	764184357	0.512	0.327	0.960	0.000	0.572	100	0.892	-0.058	0.059	0.984	0.000	100	15	5.0
	764224817	0.578	0.357	0.961	0.000	0.504	100	1.010	-0.065	0.053	0.985	0.000	100	15	6.1
	764245047	0.565	0.363	0.959	0.000	0.518	100	0.975	-0.062	0.074	0.983	0.000	100	16	6.0
	764285508	0.465	0.302	0.926	0.001	0.614	100	1.053	-0.118	0.023	0.974	0.000	100	9	3.6
	764305738	0.539	0.289	0.962	0.000	0.544	100	0.902	-0.055	0.033	0.984	0.000	100	16	5.2
	764325968	0.603	0.302	0.966	0.000	0.478	100	0.977	-0.057	0.038	0.985	0.000	100	17	6.3
	764346198	0.570	0.363	0.966	0.000	0.513	100	0.959	-0.054	0.059	0.987	0.000	100	18	6.4
	764366428	0.580	0.360	0.961	0.000	0.503	100	0.995	-0.063	0.067	0.984	0.000	100	16	6.2

	764447348	0.736	0.222	0.976	0.000	0.332	100	1.110	-0.060	-0.026	0.987	0.000	100	19	8.2
	764467579	0.675	0.277	0.981	0.000	0.402	100	1.004	-0.048	0.032	0.993	0.000	100	21	8.1
	764487809	0.611	0.294	0.975	0.000	0.471	100	0.950	-0.051	0.054	0.989	0.000	100	18	6.5
	764508039	0.776	0.158	0.984	0.000	0.285	100	1.165	-0.062	-0.100	0.994	0.000	100	19	8.6
	764588959	0.586	0.371	0.958	0.000	0.496	100	0.997	-0.059	0.066	0.982	0.000	100	17	6.6
	764649650	0.603	0.328	0.973	0.000	0.479	100	0.937	-0.044	0.056	0.989	0.000	100	21	7.2
	764669880	0.547	0.344	0.958	0.000	0.536	100	0.883	-0.051	0.107	0.978	0.000	100	17	5.7
	764710340	0.613	0.215	0.867	0.015	0.456	84	1.700	-0.252	-0.174	0.953	0.011	83	7	3.9
	764750800	0.540	0.328	0.954	0.000	0.543	100	0.955	-0.066	0.053	0.982	0.000	100	14	5.2
	764811490	0.603	0.347	0.971	0.000	0.479	100	0.943	-0.047	0.082	0.987	0.000	100	20	7.2
	764831721	0.595	0.258	0.961	0.000	0.486	100	1.040	-0.075	-0.019	0.984	0.000	100	14	5.4
	764872181	0.503	0.353	0.939	0.000	0.580	100	0.995	-0.078	0.026	0.978	0.000	100	13	4.8
	764892411	0.522	0.324	0.951	0.000	0.561	100	0.923	-0.061	0.041	0.979	0.000	100	15	5.1
	764953101	0.572	0.282	0.962	0.000	0.511	100	0.993	-0.075	0.040	0.983	0.000	100	13	5.0
	765013792	0.572	0.335	0.961	0.000	0.510	100	0.969	-0.055	0.025	0.985	0.000	100	18	6.3
	765034022	0.638	0.287	0.972	0.000	0.441	100	0.989	-0.049	0.014	0.988	0.000	100	20	7.4
	765074482	0.663	0.225	0.976	0.000	0.414	100	1.015	-0.053	-0.024	0.989	0.000	100	19	7.0
	765094712	0.566	0.301	0.956	0.000	0.514	100	1.001	-0.069	0.011	0.982	0.000	100	14	5.4
	765155402	0.596	0.277	0.974	0.000	0.486	100	0.945	-0.053	0.031	0.990	0.000	100	18	6.1
	765195863	0.600	0.337	0.975	0.000	0.482	100	0.929	-0.044	0.069	0.990	0.000	100	21	7.3
	765216093	0.554	0.357	0.966	0.000	0.530	100	0.918	-0.055	0.101	0.986	0.000	100	17	5.8
	765256553	0.551	0.329	0.954	0.000	0.531	100	0.983	-0.069	0.042	0.981	0.000	100	14	5.3
	765276783	0.518	0.314	0.952	0.000	0.564	100	0.921	-0.061	0.030	0.980	0.000	100	15	5.0
	765317243	0.546	0.319	0.948	0.000	0.536	100	0.998	-0.072	0.019	0.978	0.000	100	14	5.2
	765337473	0.532	0.382	0.967	0.000	0.552	100	0.833	-0.040	0.137	0.984	0.000	100	21	6.3
	765377934	0.518	0.232	0.960	0.000	0.565	100	0.991	-0.095	0.008	0.984	0.000	100	10	3.8
	765560005	0.497	0.307	0.963	0.000	0.587	100	0.845	-0.055	0.076	0.984	0.000	100	15	4.6
	809635352	0.633	0.323	0.972	0.000	0.447	100	1.006	-0.049	0.019	0.989	0.000	100	20	7.7
	863126187	0.581	0.290	0.975	0.000	0.502	100	0.921	-0.057	0.079	0.989	0.000	100	16	5.6
	892969023	0.618	0.296	0.969	0.000	0.463	100	1.023	-0.059	-0.005	0.988	0.000	100	17	6.7
	937495960	0.746	0.138	0.984	0.000	0.319	100	1.110	-0.065	-0.071	0.993	0.000	100	17	7.2
	953045535	0.643	0.319	0.979	0.000	0.438	100	0.981	-0.049	0.068	0.992	0.000	100	20	7.6
	970836795	0.596	0.340	0.969	0.000	0.486	100	0.977	-0.053	0.043	0.988	0.000	100	19	6.8
	**Mean**	0.594	0.305	0.963	0.001	0.486	99	1.024	-0.073	0.032	0.985	0.001	98	16	6.1
	**Std. Err.**	0.006	0.006	0.001	0.000	0.007	1	0.019	0.006	0.005	0.001	0.000	1	0	0.1
q=2	132902142	0.733	0.156	0.967	0.000	0.333	100	1.183	-0.090	-0.057	0.983	0.000	100	13	6.1
	147406386	0.397	0.489	0.829	0.002	0.680	100	0.964	-0.086	0.088	0.914	0.001	99	11	4.3
	158013734	0.671	0.263	0.968	0.000	0.404	100	1.021	-0.048	-0.010	0.982	0.000	100	21	8.0
	158114885	0.532	0.429	0.899	0.000	0.551	100	1.062	-0.070	-0.003	0.948	0.000	100	15	6.2
	158155345	0.631	0.317	0.933	0.000	0.446	100	1.184	-0.084	-0.072	0.968	0.000	100	14	6.6
	158216035	0.658	0.273	0.967	0.000	0.420	100	1.103	-0.071	-0.024	0.985	0.000	100	16	6.7
	158236265	0.488	0.483	0.839	0.001	0.592	100	1.123	-0.092	0.011	0.911	0.001	100	12	5.5
	158276726	0.698	0.266	0.980	0.000	0.375	100	1.019	-0.042	0.005	0.991	0.000	100	24	9.2
	158398106	0.467	0.440	0.896	0.000	0.614	100	0.987	-0.079	0.073	0.947	0.000	100	13	4.9
	158418336	0.581	0.278	0.927	0.000	0.498	100	1.167	-0.093	-0.111	0.968	0.000	100	13	5.3
	158438567	0.813	0.152	0.985	0.000	0.241	100	1.132	-0.048	-0.073	0.992	0.000	100	23	10.7
	158458797	0.542	0.379	0.934	0.000	0.541	100	0.981	-0.061	0.036	0.966	0.000	100	16	5.9
	158479027	0.653	0.303	0.966	0.000	0.425	100	1.121	-0.074	-0.008	0.987	0.000	100	15	6.8
	158499257	0.555	0.393	0.906	0.000	0.527	100	1.281	-0.129	-0.025	0.963	0.001	100	10	5.1
	158721788	0.434	0.397	0.849	0.002	0.643	99	1.017	-0.098	0.035	0.926	0.001	99	10	4.1
	158742018	0.705	0.281	0.947	0.000	0.367	100	1.195	-0.065	-0.119	0.971	0.000	100	18	8.7
	158802708	0.535	0.386	0.915	0.000	0.547	100	1.080	-0.082	0.002	0.960	0.000	100	13	5.5
	158822939	0.677	0.207	0.981	0.000	0.398	100	0.985	-0.049	0.002	0.990	0.000	100	20	7.2
	158883629	0.615	0.404	0.909	0.000	0.465	100	1.192	-0.080	-0.046	0.949	0.000	100	15	7.3
	158924089	0.645	0.327	0.952	0.000	0.433	100	1.141	-0.075	-0.023	0.976	0.000	100	15	7.0

	158944319	0.478	0.460	0.813	0.002	0.601	100	1.164	-0.099	-0.050	0.905	0.001	100	12	5.2
	158964549	0.874	0.061	0.988	0.000	0.165	100	1.177	-0.046	-0.153	0.993	0.000	100	26	12.0
	158984779	0.503	0.488	0.794	0.007	0.574	98	1.493	-0.164	-0.133	0.913	0.002	93	9	5.3
	159005010	0.691	0.299	0.939	0.000	0.379	100	1.186	-0.066	-0.105	0.966	0.000	100	18	8.5
	159025240	0.551	0.341	0.856	0.000	0.527	100	1.200	-0.086	-0.188	0.923	0.000	100	14	5.9
	159085930	0.522	0.529	0.810	0.001	0.556	100	1.254	-0.097	-0.067	0.896	0.000	100	13	6.6
	159146620	0.524	0.351	0.944	0.000	0.559	100	0.970	-0.068	0.036	0.976	0.000	100	14	5.2
	159166850	0.392	0.425	0.849	0.002	0.683	100	0.873	-0.073	0.083	0.925	0.001	99	12	4.0
	159227541	0.573	0.404	0.903	0.000	0.507	100	1.141	-0.075	-0.059	0.952	0.000	100	15	6.7
	159247771	0.532	0.429	0.899	0.000	0.551	100	1.062	-0.070	-0.003	0.948	0.000	100	15	6.2
	159389382	0.709	0.232	0.973	0.000	0.362	100	1.097	-0.059	-0.041	0.986	0.000	100	19	7.9
	159470302	0.833	0.146	0.980	0.000	0.215	100	1.116	-0.037	-0.084	0.987	0.000	100	30	13.3
	159713063	0.544	0.455	0.904	0.000	0.539	100	1.061	-0.069	0.033	0.952	0.000	100	15	6.5
	159733294	0.489	0.436	0.919	0.000	0.592	100	0.914	-0.056	0.090	0.960	0.000	100	16	5.6
	160744799	0.586	0.367	0.916	0.000	0.494	100	1.102	-0.075	-0.017	0.956	0.000	100	15	6.3
	160825720	0.626	0.278	0.972	0.000	0.454	100	0.995	-0.059	0.033	0.987	0.000	100	17	6.4
	160866180	0.547	0.494	0.867	0.000	0.534	100	1.192	-0.089	-0.009	0.929	0.000	100	13	6.6
	160886410	0.735	0.204	0.976	0.000	0.333	100	1.123	-0.062	-0.054	0.988	0.000	100	18	8.0
	160906640	0.443	0.388	0.893	0.001	0.636	100	0.963	-0.087	0.064	0.949	0.001	100	11	4.1
	160947100	0.478	0.460	0.813	0.002	0.601	100	1.164	-0.099	-0.050	0.905	0.001	100	12	5.2
	160967330	0.669	0.236	0.983	0.000	0.407	100	0.982	-0.050	0.028	0.992	0.000	100	20	7.2
	160987560	0.667	0.225	0.960	0.000	0.408	100	1.109	-0.070	-0.069	0.979	0.000	100	16	6.6
	161007791	0.741	0.196	0.940	0.000	0.322	100	1.347	-0.092	-0.232	0.970	0.000	100	15	7.7
	161270782	0.812	0.143	0.982	0.000	0.240	100	1.223	-0.066	-0.130	0.992	0.000	100	19	9.3
	161311242	0.564	0.311	0.952	0.000	0.518	100	1.042	-0.085	0.036	0.978	0.000	100	12	5.0
	161412393	0.458	-0.132	0.861	0.016	0.625	21	2.097	-0.418	-0.328	0.949	0.013	35	5	2.6
	161473083	0.614	0.420	0.945	0.000	0.467	100	1.080	-0.062	0.041	0.974	0.000	100	17	7.8
	161554003	0.559	0.318	0.928	0.000	0.521	100	1.088	-0.089	-0.011	0.964	0.001	100	12	5.1
	178713055	0.837	0.155	0.991	0.000	0.212	100	1.105	-0.043	-0.023	0.995	0.000	100	26	11.8
	184349034	0.643	0.238	0.942	0.002	0.432	100	1.379	-0.171	-0.021	0.972	0.005	100	8	4.4
	208027353	0.685	0.275	0.960	0.000	0.390	100	1.103	-0.058	-0.052	0.978	0.000	100	19	8.1
	246515023	0.776	0.214	0.955	0.000	0.282	100	1.120	-0.046	-0.066	0.970	0.000	100	25	11.0
	256789458	0.722	0.256	0.938	0.001	0.344	100	1.400	-0.136	-0.065	0.963	0.002	100	10	6.0
	275382046	0.740	0.184	0.936	0.007	0.317	100	1.690	-0.240	-0.084	0.965	0.020	100	7	4.6
	289996019	0.939	0.019	0.995	0.000	0.079	100	1.154	-0.038	-0.104	0.997	0.000	100	30	14.5
	295137534	0.770	0.209	0.969	0.000	0.292	100	1.239	-0.079	-0.083	0.984	0.000	100	16	8.1
	336497421	0.831	0.114	0.987	0.000	0.217	100	1.208	-0.063	-0.119	0.994	0.000	100	19	9.4
	368533040	0.396	0.535	0.776	0.006	0.679	96	1.138	-0.113	0.022	0.897	0.001	99	10	4.5
	370027359	0.465	0.430	0.883	0.001	0.615	100	1.004	-0.082	0.050	0.939	0.000	100	12	4.8
	370425937	0.579	0.375	0.947	0.000	0.502	100	1.018	-0.058	0.018	0.976	0.000	100	18	6.8
	375450439	0.518	0.446	0.916	0.000	0.565	100	0.993	-0.063	0.058	0.959	0.000	100	16	6.1
	404239096	0.633	0.299	0.959	0.000	0.445	100	1.132	-0.084	-0.011	0.982	0.000	100	14	6.1
	414519462	0.552	0.435	0.928	0.000	0.532	100	1.043	-0.065	0.035	0.968	0.000	100	16	6.6
	451588811	0.502	0.501	0.787	0.005	0.576	100	1.401	-0.143	-0.096	0.890	0.003	100	10	5.5
	465578759	0.600	0.390	0.948	0.000	0.481	100	1.026	-0.056	0.043	0.976	0.000	100	18	7.3
	492786515	0.498	0.354	0.929	0.000	0.583	100	0.928	-0.065	0.051	0.964	0.000	100	14	4.9
	508703490	0.646	0.339	0.934	0.000	0.431	100	1.167	-0.079	-0.029	0.963	0.000	100	15	7.0
	516889361	0.991	-0.014	0.999	0.000	0.011	100	1.158	-0.042	-0.062	1.000	0.000	100	27	13.6
	517810313	0.426	0.448	0.871	0.001	0.652	100	0.915	-0.074	0.100	0.936	0.000	99	12	4.4
	550534656	0.642	0.069	0.981	0.019	0.428	30	1.511	-0.213	-0.263	0.999	0.034	7	7	3.3
	553359145	0.525	0.451	0.905	0.000	0.557	100	1.032	-0.070	0.056	0.951	0.000	100	15	6.0
	604812005	0.699	0.246	0.972	0.000	0.373	100	1.122	-0.067	-0.035	0.988	0.000	100	17	7.4
	612472597	0.670	0.328	0.892	0.000	0.401	100	1.372	-0.093	-0.244	0.944	0.000	100	15	8.0
	638754422	0.504	0.467	0.900	0.000	0.578	100	0.999	-0.072	0.098	0.945	0.000	100	14	5.7
	643185023	0.692	0.296	0.957	0.000	0.381	100	1.213	-0.092	-0.004	0.977	0.000	100	13	6.7

	650853796	0.704	0.278	0.938	0.000	0.366	100	1.332	-0.100	-0.140	0.971	0.000	100	13	7.2
	658594300	0.846	0.126	0.987	0.000	0.200	100	1.225	-0.088	-0.007	0.993	0.000	100	14	7.3
	663835652	0.465	0.447	0.818	0.004	0.613	98	1.089	-0.100	0.035	0.901	0.002	99	11	4.7
	668248235	0.415	0.446	0.870	0.000	0.662	100	0.914	-0.076	0.094	0.932	0.001	100	12	4.3
	682102541	0.706	-0.226	0.967	0.033	0.367	18	0.206	0.125	-0.006	0.999	0.032	24	-2	NaN
	682449369	0.521	0.430	0.919	0.000	0.561	100	1.014	-0.068	0.046	0.963	0.000	100	15	5.9
	686765762	0.574	0.392	0.942	0.000	0.508	100	1.087	-0.078	0.030	0.975	0.000	100	14	6.1
	706846339	0.790	0.135	0.978	0.022	0.263	98	2.098	-0.389	-0.072	0.999	0.034	31	5	3.9
	737052003	0.619	0.336	0.888	0.000	0.457	100	1.250	-0.091	-0.134	0.937	0.000	100	14	6.6
	739574095	0.832	-0.076	0.940	0.020	0.190	80	2.133	-0.355	-0.381	0.990	0.020	48	6	3.7
	763395383	0.579	0.305	0.950	0.000	0.503	100	1.050	-0.075	-0.008	0.977	0.000	100	14	5.6
	763435843	0.806	0.137	0.972	0.000	0.246	100	1.245	-0.070	-0.155	0.986	0.000	100	18	8.9
	763456073	0.563	0.353	0.904	0.000	0.518	100	1.099	-0.078	-0.046	0.948	0.000	100	14	5.9
	763476303	0.657	0.324	0.893	0.000	0.416	100	1.368	-0.113	-0.148	0.938	0.000	100	12	6.6
	763516763	0.649	0.278	0.966	0.000	0.429	100	1.060	-0.062	-0.012	0.984	0.000	100	17	6.9
	763536994	0.519	0.391	0.883	0.000	0.561	100	1.085	-0.090	0.015	0.938	0.000	100	12	5.1
	763557224	0.482	0.489	0.770	0.007	0.596	96	1.352	-0.139	-0.085	0.883	0.003	97	10	5.2
	763597684	0.411	0.419	0.843	0.003	0.664	100	0.955	-0.086	0.056	0.923	0.001	99	11	4.0
	763638144	0.744	0.227	0.986	0.000	0.323	100	1.028	-0.039	0.006	0.993	0.000	100	26	10.3
	763698834	0.499	0.438	0.853	0.000	0.582	100	1.081	-0.080	-0.016	0.925	0.000	100	13	5.5
	763719065	0.652	0.323	0.961	0.000	0.426	100	1.084	-0.057	-0.029	0.983	0.000	100	19	8.0
	763759525	0.565	0.449	0.936	0.000	0.518	100	1.055	-0.065	0.050	0.971	0.000	100	16	6.9
	763820215	0.549	0.447	0.903	0.000	0.533	100	1.126	-0.083	0.018	0.953	0.000	100	13	6.2
	763840445	0.596	0.382	0.935	0.000	0.484	100	1.088	-0.065	-0.019	0.969	0.000	100	17	7.1
	763860675	0.549	0.447	0.903	0.000	0.533	100	1.126	-0.083	0.018	0.953	0.000	100	13	6.2
	763880905	0.427	0.441	0.761	0.010	0.648	79	1.335	-0.156	-0.081	0.878	0.006	92	9	4.2
	763901136	0.835	0.129	0.992	0.000	0.214	100	1.062	-0.030	-0.057	0.996	0.000	100	35	14.4
	763921366	0.582	0.417	0.868	0.000	0.496	100	1.279	-0.092	-0.151	0.931	0.000	100	14	6.9
	763982056	0.645	0.311	0.930	0.000	0.430	100	1.203	-0.085	-0.082	0.965	0.001	100	14	6.7
	764002286	0.633	0.344	0.915	0.000	0.444	100	1.236	-0.091	-0.081	0.955	0.000	100	14	6.7
	764042746	0.992	-0.047	0.988	0.000	0.003	100	1.086	-0.014	-0.117	0.990	0.000	100	80	35.1
	764062976	0.691	0.317	0.947	0.000	0.382	100	1.210	-0.072	-0.088	0.972	0.000	100	17	8.3
	764083206	0.454	0.439	0.882	0.000	0.626	100	1.006	-0.084	0.050	0.944	0.000	100	12	4.7
	764143897	0.473	0.465	0.889	0.000	0.608	100	0.981	-0.067	0.051	0.944	0.000	100	15	5.5
	764184357	0.454	0.439	0.882	0.000	0.626	100	1.006	-0.084	0.050	0.944	0.000	100	12	4.7
	764224817	0.633	0.327	0.958	0.000	0.446	100	1.090	-0.069	0.004	0.981	0.000	100	16	6.8
	764245047	0.455	0.511	0.825	0.002	0.624	100	1.121	-0.101	0.042	0.912	0.001	100	11	5.1
	764285508	0.608	0.234	0.943	0.000	0.468	100	1.278	-0.135	-0.083	0.979	0.000	100	10	4.6
	764305738	0.594	0.275	0.950	0.000	0.485	100	1.058	-0.070	-0.052	0.979	0.000	100	15	5.8
	764325968	0.593	0.329	0.959	0.000	0.489	100	1.012	-0.063	0.034	0.982	0.000	100	16	6.2
	764346198	0.518	0.428	0.912	0.000	0.564	100	1.012	-0.068	0.043	0.954	0.000	100	15	5.8
	764366428	0.627	0.356	0.936	0.000	0.453	100	1.130	-0.076	0.001	0.966	0.000	100	15	6.8
	764447348	0.724	0.268	0.937	0.000	0.342	100	1.297	-0.091	-0.113	0.962	0.000	100	14	7.6
	764467579	0.726	0.287	0.955	0.000	0.343	100	1.213	-0.070	-0.076	0.977	0.000	100	17	8.7
	764487809	0.562	0.382	0.938	0.000	0.519	100	1.048	-0.074	0.039	0.972	0.000	100	14	5.9
	764508039	0.984	-0.054	0.994	0.000	0.018	100	1.182	-0.031	-0.185	0.995	0.000	100	38	18.5
	764588959	0.643	0.338	0.943	0.000	0.435	100	1.143	-0.072	-0.033	0.970	0.000	100	16	7.2
	764649650	0.534	0.469	0.867	0.000	0.548	100	1.129	-0.079	-0.016	0.928	0.000	100	14	6.4
	764669880	0.534	0.398	0.916	0.000	0.549	100	1.046	-0.078	0.037	0.955	0.000	100	13	5.5
	764710340	0.782	-0.424	0.941	0.002	0.277	16	2.326	-0.416	-0.421	0.945	0.013	51	6	3.5
	764750800	0.490	0.434	0.864	0.001	0.589	100	1.102	-0.097	0.028	0.930	0.001	100	11	5.0
	764811490	0.555	0.450	0.921	0.000	0.528	100	1.065	-0.071	0.052	0.961	0.000	100	15	6.5
	764831721	0.607	0.263	0.933	0.000	0.470	100	1.217	-0.102	-0.116	0.972	0.000	100	12	5.4
	764872181	0.559	0.375	0.859	0.001	0.520	100	1.310	-0.120	-0.124	0.926	0.001	100	11	5.5
	764892411	0.493	0.348	0.922	0.000	0.587	100	0.969	-0.072	0.013	0.966	0.000	100	13	4.8

	764953101	0.567	0.319	0.938	0.000	0.516	100	1.110	-0.096	0.007	0.971	0.000	100	12	5.0
	765013792	0.566	0.418	0.859	0.000	0.514	100	1.246	-0.094	-0.113	0.929	0.000	100	13	6.4
	765034022	0.768	0.198	0.973	0.000	0.292	100	1.149	-0.053	-0.100	0.985	0.000	100	22	9.9
	765074482	0.705	0.209	0.964	0.000	0.366	100	1.157	-0.069	-0.110	0.982	0.000	100	17	7.4
	765094712	0.611	0.299	0.932	0.000	0.466	100	1.189	-0.092	-0.085	0.970	0.000	100	13	5.9
	765155402	0.580	0.325	0.946	0.000	0.503	100	1.051	-0.071	-0.007	0.975	0.000	100	15	5.9
	765195863	0.565	0.453	0.877	0.000	0.515	100	1.175	-0.081	-0.045	0.936	0.000	100	15	6.9
	765216093	0.585	0.356	0.961	0.000	0.497	100	0.981	-0.060	0.077	0.982	0.000	100	16	6.3
	765256553	0.547	0.393	0.880	0.000	0.534	100	1.195	-0.103	-0.038	0.939	0.000	100	12	5.4
	765276783	0.441	0.363	0.898	0.000	0.637	100	0.910	-0.071	0.032	0.949	0.000	100	13	4.2
	765317243	0.533	0.406	0.843	0.002	0.545	100	1.284	-0.119	-0.092	0.922	0.001	100	11	5.3
	765337473	0.495	0.474	0.860	0.000	0.586	100	1.018	-0.069	0.047	0.921	0.000	100	15	5.8
	765377934	0.550	0.240	0.939	0.000	0.530	100	1.132	-0.117	-0.035	0.973	0.001	100	10	4.1
	765560005	0.425	0.377	0.882	0.001	0.651	100	0.884	-0.073	0.072	0.936	0.000	100	12	4.0
	809635352	0.719	0.248	0.966	0.000	0.350	100	1.053	-0.044	-0.024	0.978	0.000	100	24	9.6
	863126187	0.559	0.371	0.935	0.000	0.525	100	1.101	-0.091	0.035	0.971	0.000	100	12	5.4
	892969023	0.602	0.358	0.901	0.000	0.477	100	1.225	-0.090	-0.106	0.949	0.000	100	14	6.5
	937495960	0.787	0.154	0.974	0.000	0.271	100	1.310	-0.093	-0.147	0.989	0.000	100	14	7.5
	953045535	0.648	0.359	0.929	0.000	0.430	100	1.175	-0.076	-0.034	0.959	0.000	100	15	7.4
	970836795	0.697	0.280	0.909	0.000	0.373	100	1.303	-0.084	-0.192	0.945	0.000	100	16	8.0
	**Mean**	0.612	0.315	0.920	0.001	0.460	98	1.151	-0.088	-0.038	0.960	0.001	97	15	6.8
	**Std. Err.**	0.010	0.012	0.004	0.000	0.011	1	0.018	0.005	0.007	0.002	0.000	1	1	0.3
q=3	132902142	0.809	0.109	0.970	0.000	0.243	100	1.180	-0.074	-0.066	0.982	0.001	100	16	7.5
	147406386	0.380	0.538	0.772	0.006	0.694	100	1.058	-0.101	0.051	0.895	0.002	94	10	4.4
	158013734	0.732	0.216	0.973	0.000	0.335	100	1.055	-0.045	-0.036	0.983	0.000	100	24	9.4
	158114885	0.533	0.471	0.868	0.000	0.550	100	1.130	-0.079	-0.016	0.929	0.000	100	14	6.4
	158155345	0.649	0.337	0.911	0.000	0.427	100	1.317	-0.101	-0.134	0.956	0.000	100	13	6.9
	158216035	0.699	0.275	0.950	0.000	0.372	100	1.232	-0.085	-0.079	0.973	0.000	100	15	7.3
	158236265	0.475	0.529	0.774	0.004	0.603	100	1.245	-0.111	-0.044	0.875	0.002	99	11	5.6
	158276726	0.785	0.181	0.985	0.000	0.275	100	1.036	-0.033	-0.024	0.991	0.000	100	31	12.2
	158398106	0.466	0.464	0.860	0.001	0.614	100	1.070	-0.091	0.038	0.928	0.001	100	12	5.0
	158418336	0.633	0.260	0.924	0.000	0.443	100	1.263	-0.100	-0.159	0.965	0.000	100	13	5.9
	158438567	0.890	0.080	0.989	0.000	0.144	100	1.148	-0.039	-0.102	0.992	0.000	100	29	13.9
	158458797	0.538	0.395	0.920	0.000	0.543	100	0.986	-0.062	0.046	0.955	0.000	100	16	6.0
	158479027	0.668	0.298	0.966	0.000	0.409	100	1.122	-0.072	-0.003	0.985	0.000	100	16	7.0
	158499257	0.405	0.541	0.767	0.012	0.671	86	1.408	-0.181	-0.012	0.913	0.003	97	8	4.4
	158721788	0.377	0.389	0.765	0.010	0.696	73	1.083	-0.125	0.025	0.872	0.006	89	9	3.6
	158742018	0.715	0.302	0.927	0.000	0.354	100	1.287	-0.076	-0.164	0.958	0.000	100	17	9.0
	158802708	0.523	0.433	0.868	0.000	0.558	100	1.201	-0.103	-0.045	0.938	0.000	100	12	5.5
	158822939	0.763	0.154	0.983	0.000	0.299	100	1.081	-0.051	-0.057	0.991	0.000	100	21	8.8
	158883629	0.651	0.381	0.911	0.000	0.426	100	1.230	-0.080	-0.071	0.948	0.000	100	15	7.8
	158924089	0.667	0.333	0.935	0.000	0.408	100	1.220	-0.084	-0.058	0.963	0.000	100	15	7.3
	158944319	0.506	0.448	0.782	0.004	0.572	99	1.291	-0.113	-0.137	0.886	0.001	98	11	5.6
	158964549	0.920	0.029	0.986	0.000	0.105	100	1.172	-0.038	-0.149	0.990	0.000	100	31	14.7
	158984779	0.504	0.496	0.755	0.009	0.574	80	1.632	-0.194	-0.174	0.878	0.005	92	8	5.3
	159005010	0.749	0.248	0.943	0.000	0.312	100	1.218	-0.062	-0.134	0.964	0.000	100	20	9.7
	159025240	0.565	0.355	0.811	0.001	0.510	100	1.341	-0.103	-0.278	0.896	0.001	100	13	6.2
	159085930	0.519	0.553	0.789	0.001	0.559	100	1.293	-0.103	-0.078	0.884	0.000	100	13	6.7
	159146620	0.541	0.369	0.933	0.000	0.541	100	1.045	-0.076	0.013	0.971	0.000	100	14	5.5
	159166850	0.393	0.422	0.811	0.005	0.681	98	0.905	-0.078	0.064	0.897	0.001	98	12	4.0
	159227541	0.529	0.489	0.838	0.001	0.550	100	1.232	-0.093	-0.084	0.919	0.000	100	13	6.5
	159247771	0.533	0.471	0.868	0.000	0.550	100	1.130	-0.079	-0.016	0.929	0.000	100	14	6.4
	159389382	0.717	0.253	0.965	0.000	0.353	100	1.160	-0.067	-0.059	0.982	0.000	100	17	8.1
	159470302	0.899	0.086	0.980	0.000	0.132	100	1.084	-0.025	-0.066	0.984	0.000	100	44	19.2
	159713063	0.545	0.496	0.862	0.000	0.537	100	1.170	-0.083	-0.013	0.926	0.000	100	14	6.8

	159733294	0.491	0.438	0.906	0.000	0.590	100	0.897	-0.054	0.108	0.946	0.000	100	17	5.7
	160744799	0.566	0.419	0.883	0.000	0.513	100	1.194	-0.091	-0.048	0.940	0.000	100	13	6.3
	160825720	0.676	0.255	0.974	0.000	0.400	100	1.036	-0.057	0.016	0.987	0.000	100	18	7.2
	160866180	0.514	0.558	0.805	0.001	0.566	100	1.287	-0.107	-0.046	0.896	0.001	100	12	6.5
	160886410	0.798	0.156	0.979	0.000	0.258	100	1.177	-0.060	-0.096	0.989	0.000	100	20	9.3
	160906640	0.404	0.439	0.833	0.004	0.671	99	0.975	-0.096	0.084	0.915	0.003	99	10	3.9
	160947100	0.506	0.448	0.782	0.004	0.572	99	1.291	-0.113	-0.137	0.886	0.001	98	11	5.6
	160967330	0.724	0.206	0.986	0.000	0.346	100	1.034	-0.049	0.000	0.994	0.000	100	21	8.3
	160987560	0.765	0.133	0.969	0.000	0.296	100	1.128	-0.058	-0.108	0.980	0.000	100	20	8.3
	161007791	0.764	0.196	0.931	0.000	0.294	100	1.421	-0.100	-0.267	0.963	0.000	100	14	8.1
	161270782	0.948	-0.009	0.987	0.000	0.065	100	1.158	-0.034	-0.149	0.989	0.000	100	35	16.4
	161311242	0.553	0.359	0.921	0.000	0.528	100	1.137	-0.103	0.023	0.960	0.000	100	11	5.0
	161412393	0.448	-0.130	0.857	0.017	0.634	20	2.206	-0.449	-0.329	0.946	0.013	33	5	2.7
	161473083	0.616	0.437	0.928	0.000	0.465	100	1.123	-0.067	0.023	0.962	0.000	100	17	7.9
	161554003	0.681	0.208	0.939	0.001	0.389	98	1.115	-0.073	-0.061	0.959	0.002	99	15	6.4
	178713055	0.927	0.065	0.997	0.000	0.099	100	1.086	-0.025	-0.041	0.998	0.000	100	43	19.2
	184349034	0.678	0.225	0.944	0.002	0.392	100	1.387	-0.164	-0.028	0.971	0.005	98	8	4.7
	208027353	0.726	0.267	0.959	0.000	0.343	100	1.136	-0.057	-0.053	0.974	0.000	100	20	9.2
	246515023	0.915	0.043	0.963	0.000	0.106	100	1.053	-0.018	-0.069	0.970	0.000	100	57	23.2
	256789458	0.743	0.247	0.935	0.001	0.319	100	1.452	-0.142	-0.089	0.961	0.002	99	10	6.3
	275382046	0.768	0.167	0.942	0.005	0.286	100	1.695	-0.234	-0.095	0.968	0.017	100	7	4.8
	289996019	0.993	-0.032	0.996	0.000	0.006	100	1.101	-0.019	-0.094	0.997	0.000	100	57	26.2
	295137534	0.813	0.170	0.975	0.000	0.240	100	1.220	-0.068	-0.083	0.985	0.000	100	18	9.1
	336497421	0.942	0.008	0.994	0.000	0.076	100	1.142	-0.034	-0.117	0.996	0.000	100	34	15.9
	368533040	0.402	0.516	0.755	0.008	0.674	83	1.211	-0.127	-0.005	0.871	0.003	99	10	4.5
	370027359	0.438	0.470	0.850	0.002	0.641	100	1.016	-0.088	0.062	0.921	0.001	100	12	4.6
	370425937	0.616	0.358	0.940	0.000	0.463	100	1.091	-0.063	-0.028	0.971	0.000	100	17	7.3
	375450439	0.564	0.421	0.911	0.000	0.518	100	1.037	-0.063	0.035	0.949	0.000	100	17	6.7
	404239096	0.675	0.282	0.959	0.000	0.400	100	1.173	-0.084	-0.028	0.980	0.000	100	14	6.7
	414519462	0.564	0.445	0.918	0.000	0.519	100	1.061	-0.066	0.040	0.960	0.000	100	16	6.9
	451588811	0.495	0.519	0.748	0.008	0.583	88	1.541	-0.169	-0.159	0.870	0.004	95	9	5.5
	465578759	0.678	0.301	0.956	0.000	0.396	100	1.007	-0.044	0.032	0.972	0.000	100	23	8.9
	492786515	0.535	0.333	0.931	0.000	0.547	100	0.946	-0.062	0.043	0.960	0.000	100	15	5.3
	508703490	0.663	0.342	0.913	0.000	0.411	100	1.252	-0.089	-0.074	0.947	0.000	100	14	7.3
	516889361	1.008	-0.029	0.999	0.000	-0.013	100	1.141	-0.033	-0.067	1.000	0.000	100	34	16.8
	517810313	0.447	0.456	0.842	0.002	0.631	99	1.007	-0.085	0.061	0.914	0.000	99	12	4.7
	550534656	0.703	0.087	0.979	0.021	0.364	40	1.279	-0.121	-0.232	1.000	0.028	11	11	4.5
	553359145	0.519	0.485	0.870	0.000	0.562	100	1.093	-0.079	0.036	0.929	0.000	100	14	6.1
	604812005	0.759	0.210	0.974	0.000	0.305	100	1.178	-0.067	-0.068	0.987	0.000	100	18	8.5
	612472597	0.617	0.416	0.841	0.000	0.458	100	1.463	-0.112	-0.273	0.921	0.000	100	13	7.5
	638754422	0.478	0.505	0.872	0.000	0.603	100	0.994	-0.075	0.121	0.927	0.000	100	13	5.5
	643185023	0.709	0.287	0.949	0.000	0.361	100	1.262	-0.098	-0.031	0.971	0.000	100	13	6.9
	650853796	0.694	0.306	0.914	0.000	0.375	100	1.392	-0.111	-0.158	0.955	0.000	100	13	7.2
	658594300	0.927	0.053	0.995	0.000	0.096	100	1.125	-0.046	-0.017	0.997	0.000	100	24	11.7
	663835652	0.468	0.451	0.779	0.007	0.609	92	1.172	-0.113	-0.003	0.874	0.004	95	10	4.8
	668248235	0.433	0.449	0.854	0.001	0.645	100	0.958	-0.080	0.078	0.918	0.001	100	12	4.5
	682102541	0.578	0.051	0.979	0.021	0.495	27	0.417	0.050	0.069	1.000	0.017	27	-8	NaN
	682449369	0.557	0.421	0.902	0.000	0.523	100	1.104	-0.076	-0.005	0.949	0.000	100	15	6.4
	686765762	0.581	0.417	0.916	0.000	0.500	100	1.175	-0.090	-0.003	0.959	0.000	100	13	6.3
	706846339	0.714	0.196	0.969	0.031	0.357	76	2.253	-0.458	-0.034	0.999	0.042	31	5	3.7
	737052003	0.581	0.409	0.829	0.001	0.495	100	1.368	-0.114	-0.176	0.909	0.001	100	12	6.4
	739574095	0.867	-0.095	0.940	0.020	0.147	80	2.085	-0.336	-0.384	0.993	0.014	42	6	3.8
	763395383	0.638	0.262	0.954	0.000	0.440	100	1.081	-0.071	-0.032	0.976	0.000	100	15	6.3
	763435843	0.959	-0.033	0.980	0.000	0.049	100	1.142	-0.029	-0.155	0.986	0.000	100	39	18.0
	763456073	0.593	0.344	0.879	0.000	0.485	100	1.197	-0.087	-0.106	0.931	0.000	100	14	6.2

	763476303	0.673	0.332	0.873	0.000	0.397	100	1.451	-0.124	-0.185	0.924	0.000	100	12	6.9
	763516763	0.700	0.246	0.968	0.000	0.372	100	1.125	-0.065	-0.054	0.985	0.000	100	17	7.7
	763536994	0.524	0.410	0.842	0.001	0.553	100	1.169	-0.103	-0.019	0.910	0.001	100	11	5.2
	763557224	0.487	0.486	0.754	0.008	0.591	89	1.434	-0.152	-0.123	0.870	0.003	95	9	5.3
	763597684	0.409	0.408	0.797	0.005	0.666	88	1.023	-0.100	0.029	0.889	0.003	95	10	4.0
	763638144	0.789	0.192	0.986	0.000	0.270	100	1.059	-0.037	-0.018	0.991	0.000	100	28	11.8
	763698834	0.513	0.446	0.816	0.001	0.566	100	1.182	-0.092	-0.078	0.905	0.000	99	13	5.8
	763719065	0.669	0.332	0.952	0.000	0.406	100	1.135	-0.062	-0.048	0.977	0.000	100	18	8.3
	763759525	0.563	0.478	0.915	0.000	0.520	100	1.102	-0.071	0.039	0.959	0.000	100	15	7.0
	763820215	0.551	0.460	0.889	0.000	0.530	100	1.159	-0.088	0.008	0.944	0.000	100	13	6.3
	763840445	0.591	0.399	0.916	0.000	0.489	100	1.090	-0.066	-0.008	0.954	0.000	100	16	7.1
	763860675	0.551	0.460	0.889	0.000	0.530	100	1.159	-0.088	0.008	0.944	0.000	100	13	6.3
	763880905	0.429	0.416	0.748	0.012	0.646	64	1.467	-0.180	-0.140	0.868	0.006	83	8	4.4
	763901136	0.907	0.060	0.994	0.000	0.123	100	1.087	-0.024	-0.086	0.996	0.000	100	46	19.6
	763921366	0.563	0.476	0.832	0.000	0.516	100	1.355	-0.105	-0.169	0.913	0.000	100	13	7.0
	763982056	0.823	0.127	0.951	0.001	0.222	100	1.179	-0.053	-0.125	0.968	0.001	99	22	10.5
	764002286	0.649	0.362	0.889	0.000	0.425	100	1.361	-0.108	-0.141	0.939	0.000	100	13	7.0
	764042746	1.007	-0.057	0.989	0.000	-0.017	100	1.074	-0.010	-0.107	0.991	0.000	100	110	48.1
	764062976	0.701	0.333	0.911	0.000	0.368	100	1.336	-0.088	-0.163	0.949	0.000	100	15	8.5
	764083206	0.445	0.494	0.833	0.002	0.634	99	1.091	-0.098	0.041	0.914	0.001	100	11	4.8
	764143897	0.476	0.463	0.878	0.000	0.604	100	0.971	-0.066	0.059	0.933	0.000	100	15	5.5
	764184357	0.445	0.494	0.833	0.002	0.634	99	1.091	-0.098	0.041	0.914	0.001	100	11	4.8
	764224817	0.700	0.270	0.964	0.000	0.371	100	1.099	-0.060	-0.011	0.979	0.000	100	18	8.0
	764245047	0.461	0.513	0.789	0.004	0.617	97	1.188	-0.111	0.006	0.884	0.002	99	11	5.1
	764285508	0.777	0.110	0.967	0.000	0.277	100	1.367	-0.119	-0.169	0.985	0.000	100	12	6.1
	764305738	0.614	0.294	0.927	0.000	0.464	100	1.179	-0.086	-0.105	0.966	0.000	100	14	6.1
	764325968	0.607	0.325	0.956	0.000	0.474	100	1.034	-0.065	0.024	0.979	0.000	100	16	6.4
	764346198	0.587	0.363	0.916	0.000	0.493	100	1.041	-0.063	0.009	0.948	0.000	100	17	6.6
	764366428	0.691	0.299	0.944	0.000	0.382	100	1.156	-0.070	-0.029	0.966	0.000	100	16	7.8
	764447348	0.715	0.296	0.915	0.000	0.351	100	1.389	-0.107	-0.152	0.950	0.000	100	13	7.5
	764467579	0.757	0.267	0.957	0.000	0.307	100	1.238	-0.070	-0.090	0.976	0.000	100	18	9.4
	764487809	0.505	0.464	0.886	0.000	0.576	100	1.111	-0.092	0.037	0.948	0.000	100	12	5.5
	764508039	1.026	-0.094	0.996	0.000	-0.040	100	1.153	-0.020	-0.178	0.997	0.000	100	57	27.9
	764588959	0.667	0.326	0.930	0.000	0.408	100	1.210	-0.078	-0.077	0.960	0.000	100	15	7.6
	764649650	0.518	0.523	0.802	0.001	0.561	100	1.248	-0.097	-0.072	0.890	0.001	100	13	6.5
	764669880	0.585	0.357	0.913	0.000	0.496	100	1.100	-0.078	-0.006	0.947	0.000	100	14	6.1
	764710340	0.748	-0.397	0.937	0.002	0.318	16	2.407	-0.442	-0.427	0.937	0.018	51	5	3.5
	764750800	0.498	0.442	0.818	0.002	0.579	100	1.166	-0.106	-0.003	0.895	0.003	100	11	5.1
	764811490	0.522	0.517	0.878	0.000	0.561	100	1.126	-0.083	0.046	0.938	0.000	100	13	6.3
	764831721	0.636	0.258	0.916	0.000	0.438	100	1.330	-0.116	-0.173	0.962	0.000	100	11	5.7
	764872181	0.598	0.362	0.850	0.001	0.479	100	1.406	-0.129	-0.175	0.919	0.001	100	11	5.9
	764892411	0.516	0.342	0.917	0.000	0.565	100	1.006	-0.074	-0.004	0.962	0.000	100	14	5.0
	764953101	0.578	0.334	0.915	0.000	0.503	100	1.228	-0.115	-0.040	0.960	0.000	100	11	5.1
	765013792	0.550	0.478	0.808	0.001	0.529	100	1.358	-0.112	-0.152	0.900	0.001	100	12	6.6
	765034022	0.813	0.170	0.968	0.000	0.238	100	1.210	-0.055	-0.140	0.980	0.000	100	22	10.9
	765074482	0.742	0.190	0.954	0.000	0.321	100	1.225	-0.073	-0.151	0.975	0.000	100	17	8.0
	765094712	0.672	0.266	0.932	0.000	0.399	100	1.282	-0.097	-0.139	0.967	0.000	100	13	6.6
	765155402	0.608	0.329	0.929	0.000	0.473	100	1.128	-0.079	-0.038	0.962	0.000	100	14	6.3
	765195863	0.570	0.478	0.847	0.000	0.509	100	1.260	-0.091	-0.084	0.918	0.000	100	14	7.1
	765216093	0.644	0.318	0.966	0.000	0.435	100	1.011	-0.056	0.058	0.983	0.000	100	18	7.2
	765256553	0.531	0.449	0.827	0.002	0.549	100	1.323	-0.126	-0.079	0.914	0.001	99	11	5.5
	765276783	0.451	0.358	0.870	0.001	0.627	100	0.933	-0.073	0.018	0.923	0.001	100	13	4.3
	765317243	0.530	0.433	0.811	0.004	0.548	100	1.358	-0.132	-0.117	0.903	0.001	100	10	5.4
	765337473	0.519	0.475	0.810	0.001	0.560	100	1.145	-0.083	-0.035	0.888	0.000	100	14	6.2
	765377934	0.604	0.229	0.933	0.001	0.472	100	1.246	-0.129	-0.075	0.969	0.001	100	10	4.5

	765560005	0.418	0.387	0.821	0.003	0.657	93	0.982	-0.091	0.028	0.899	0.002	96	11	4.0
	809635352	0.747	0.226	0.965	0.000	0.317	100	1.047	-0.040	-0.018	0.975	0.000	100	26	10.6
	863126187	0.568	0.393	0.901	0.000	0.514	100	1.223	-0.110	-0.013	0.950	0.000	100	11	5.5
	892969023	0.625	0.359	0.886	0.000	0.451	100	1.278	-0.094	-0.127	0.935	0.000	100	14	6.9
	937495960	0.765	0.214	0.949	0.000	0.297	100	1.403	-0.113	-0.153	0.973	0.000	100	12	7.2
	953045535	0.675	0.344	0.905	0.000	0.397	100	1.273	-0.086	-0.101	0.940	0.000	100	15	7.8
	970836795	0.738	0.244	0.892	0.000	0.323	100	1.375	-0.088	-0.253	0.929	0.000	100	16	8.6
	**Mean**	0.635	0.311	0.902	0.002	0.433	97	1.207	-0.093	-0.064	0.948	0.002	97	17	7.8
	**Std. Err.**	0.012	0.014	0.006	0.000	0.014	1	0.019	0.005	0.008	0.003	0.000	1	1	0.4
